# Statine-based peptidomimetic compounds as inhibitors for SARS-CoV-2 main protease (SARS-CoV‑2 Mpro)

**DOI:** 10.1038/s41598-024-59442-4

**Published:** 2024-04-18

**Authors:** Pedro Henrique R. de A. Azevedo, Priscila G. Camargo, Larissa E. C. Constant, Stephany da S. Costa, Celimar Sinézia Silva, Alice S. Rosa, Daniel D. C. Souza, Amanda R. Tucci, Vivian N. S. Ferreira, Thamara Kelcya F. Oliveira, Nathalia R. R. Borba, Carlos R. Rodrigues, Magaly G. Albuquerque, Luiza R. S. Dias, Rafael Garrett, Milene D. Miranda, Diego Allonso, Camilo Henrique da S. Lima, Estela Maris F. Muri

**Affiliations:** 1 Laboratório de Química Medicinal, Faculdade de Farmácia, Universidade Federal Fluminense, Niterói, RJ 24241-000 Brazil; 2 Faculdade de Farmácia, Universidade Federal do Rio de Janeiro, Rio de Janeiro, RJ 21941-853 Brazil; 3 Laboratório de Biotecnologia e Bioengenharia Tecidual, Instituto de Biofísica Carlos Chagas Filho, Universidade Federal do Rio de Janeiro, Rio de Janeiro, RJ 21941-853 Brazil; 4 Laboratório de Morfologia e Morfogênese Viral, Instituto Oswaldo Cruz, Fundação Oswaldo Cruz, Rio de Janeiro, RJ 21040-900 Brazil; 5 Programa de Pós-Graduação em Biologia Celular e Molecular, Instituto Oswaldo Cruz, Fundação Oswaldo Cruz, Rio de Janeiro, RJ 21040-900 Brazil; 6 Programa de Pós-Graduação em Química, Instituto de Química, Universidade Federal do Rio de Janeiro, Rio de Janeiro, RJ 21941-853 Brazil; 7 Departamento de Biotecnologia Farmacêutica, Faculdade de Farmácia, Universidade Federal do Rio de Janeiro, Rio de Janeiro, RJ 21941-853 Brazil

**Keywords:** Drug discovery, Medicinal chemistry, Pharmacology

## Abstract

COVID-19 is a multisystemic disease caused by the SARS-CoV-2 airborne virus, a member of the *Coronaviridae* family. It has a positive sense single-stranded RNA genome and encodes two non-structural proteins through viral cysteine-proteases processing. Blocking this step is crucial to control virus replication. In this work, we reported the synthesis of 23 statine-based peptidomimetics to determine their ability to inhibit the main protease (Mpro) activity of SARS-CoV-2. Among the 23 peptidomimetics, 15 compounds effectively inhibited Mpro activity by 50% or more, while three compounds (**7d**, **8e**, and **9g**) exhibited maximum inhibition above 70% and IC_50_ < 1 µM. Compounds **7d**, **8e,** and **9g** inhibited roughly 80% of SARS-CoV-2 replication and proved no cytotoxicity. Molecular docking simulations show putative hydrogen bond and hydrophobic interactions between specific amino acids and these inhibitors. Molecular dynamics simulations further confirmed the stability and persisting interactions in Mpro's subsites, exhibiting favorable free energy binding (ΔG_bind_) values. These findings suggest the statine-based peptidomimetics as potential therapeutic agents against SARS-CoV-2 by targeting Mpro.

## Introduction

Coronavirus disease-19 (COVID-19) emerged in late 2019 as a massive pandemic with catastrophic effects on people's lives, accounting for more than 770 million cases and over 7 million deaths worldwide^1^. It is caused by the severe acute respiratory syndrome coronavirus 2 (SARS-CoV-2), an enveloped airborne virus member of the *Coronaviridae* family (genus *Betacoronavirus*), which also contains other life-threatening viruses, such as SARS-CoV-1 and Middle East respiratory syndrome coronavirus (MERS-CoV)^2^. The genome of SARS-CoV-2 has a positive-sense single-strand RNA of 27–32 kb-long that encodes for two open reading frames (ORF1a and ORF1b), which are translated into large polyproteins (Pp1a and Pp1ab) further processed into 16 nonstructural proteins (nsps) by two virus-encoded cysteine proteases: the chymotrypsin-like protease (3CLpro), also called main protease (Mpro), responsible for polyprotein cleavage at 11 sites, generating 13 nsps, and the papain-like protease (PLpro), which cleaves the polyprotein at three other sites, generating 4 nsps^3^. Pp1a and Pp1ab processing is crucial on the virus replicative cycle and, consequently, for proper virus infection. Therefore, blockage of polyprotein processing is an outstanding strategy to control virus replication. Mpro is an excellent pharmacological target due to its relevance to the virus life cycle and the absence of a homologous enzyme in humans. Nowadays, there are several available vaccines against SARS-CoV-2 in the world, which exerted and still does an essential role for controlling severe cases and death. However, they failed to prevent mild and moderate infection, which is managed using antiviral or symptoms- relieving drugs. Despite the quick development of several vaccines, only a few antivirals targeting SARS-CoV-2 are available in the market, such as nirmatrelvir-ritonavir combination, molnupiravir, and remdesivir^4^, and most of them are not indicated for mild and moderate cases, which makes the development of new drugs urgent.

The scientific community has been trying to find active molecules against SARS-CoV-2 using strategies such as repurposing of existing drugs, bioinformatics, pharmacoinformatic approaches, and others^5^. One of the most important classes of molecules studied is the peptidomimetic compounds, including different analogs such as keto-glutamine, trifluoromethyl ketone, α,β-unsaturated esters, and α-keto amides^6–11^.

A class of unusual (non-canonical) amino acids^12^ known as γ-amino-β-hydroxy acids, such as statine (PubChem CID 123,915; (3*S*,4*S*)-4-amino-3-hydroxy-6-methylheptanoic acid; AHMHA) and the statine-like AHPPA^13^ (PubChem CID 11,769,728; (3*S*,4*S*)-4-amino-3-hydroxy-5-phenylpentanoic acid) are mainly present as amino acid residues in several examples of natural peptides (e.g., pepstatin-A, ahpatinin-Ac)^14,15^ and synthetic peptidomimetic inhibitors of aspartic proteases^16^ and there is evidence that its activity against viral infections occurs by impairing virus protease activity^17,18^. Despite that, and as far as we know, there are no statine-based synthetic compounds described as cysteine inhibitors of SARS-CoV-2 Mpro.

Our research group has focused on protease inhibitors, and we have reported a new class of statine-based peptidomimetics as selective inhibitors of the human kallikrein 5 (KLK5) serine-protease activity^18^.

In the current study we repositioned, synthesized, and investigated our statine-based peptidomimetics as cysteine protease inhibitors against SARS-CoV-2 Mpro, including antiviral activity in cell model assay, cytotoxicity assays, and in silico evaluation.

## Materials and methods

### Chemistry

#### General information

Reagents were purchased from Sigma-Aldrich Co. All solvents were purchased as reagent grade, dried using standard conditions, and stored over molecular sieves. Purification of products was carried out using silica gel flash chromatography (Whatman 60, 230–400 mesh). NMR analyses were performed on a Varian Unity Plus-300 spectrometer. Melting points were obtained on a Thomas Hoover capillary melting point apparatus and are uncorrected. All compounds are > 95% pure by high-resolution mass spectra (HRMS) that were performed on a Waters Micromass Q-Tof Micromass spectrometer equipped with a lock spray source. The IR spectra were obtained on a Perkin-Elmer spectrometer model Spectrum One in liquid film or KBr pellets. Optical rotation measurements were determined on a Perkin-Elmer 341 LC polarimeter.

#### General procedure for compounds 7a-h

To a 0 ºC cooled mixture of compound **1** (0.2 g; 0.647 mmol) and the appropriate methyl ester hydrochloride (1.15 mmol) in dry CH_2_Cl_2_ (10 mL) were added EDC.HCl (0.186 g; 0.970 mmol), HOBt (0.13 g; 0.970 mmol) and *N*-methylmorpholine (0.21 mL; 1.94 mmol). The mixture was stirred at room temperature for 24 h, and the volatiles were removed under reduced pressure. The resulting residue was dissolved in CH_2_Cl_2_ (50 mL) and successively washed with 5% H_3_PO_4_ (50 mL), 20% Na_2_CO_3_ (50 mL), water (40 mL), and brine (50 mL) and dried with Na_2_SO_4_ after which it was filtered and evaporated under reduced pressure. The products were purified by flash chromatography on silica gel using EtOAc/hexane as eluents.

#### General procedure for compounds 8a-g

The corresponding ester **7a-g** (5.0 mmol; 1 eq.) was solubilized in distilled dichloromethane (5.0 mL). Anhydrous pyridine (7.5 mmol; 0.6 mL; 1.5 eq.) and DMAP (5.0 mmol; 1 eq.) were added to the solution. The mixture was cooled to 0 °C and then acetic anhydride (7.5 mmol; 1.5 eq.) was added dropwise. The reaction was stirred at room temperature for 3 h until complete consumption of the starting material. The reaction was diluted with dichloromethane (50 mL) and extracted successively with water (50 mL) and brine (50 mL). The organic phase was dried over anhydrous sodium sulfate and then evaporated on a rotary evaporator. Purification by chromatographic column on silica gel afforded the products as solids.

#### General procedure for compounds 9a-g

The corresponding ester (**7a-h**) (5.0 mmol) was solubilized in a minimum volume of distilled dichloromethane. The resulting solution was cooled to 0 °C and then trifluoroacetic acid (7.50 mmol, 0.57 mL, 1.5 eq) was added dropwise. The reaction was stirred at room temperature for 3 h until complete consumption of the starting material and then completely evaporated in a rotary evaporator. The resulting product was purified by recrystallization from diethyl ether.

### Biological assays

#### In vitro inhibition of SARS-CoV-2 Mpro

Recombinant SARS-CoV-2 Mpro synthetic gene expressed in *E. coli* BL21(DE3)pLysS cells were used in a fluorescent resonance energy transfer (FRET) assay, using as substrate the DABCYL-AVLQ↓SGFRLL-EDANS peptide (Biomatik Corp., CA), and as positive control a synthetic dipeptide covalent inhibitor of SARS-CoV’s Mpro, **GC-376** (PubChem CID 71,481,119). The enzyme concentration was fixed at 1.5 μM, the substrate at 50 μM and the compounds (statine-based peptidomimetics and **GC-376**) ranged from 0.001 to 1000 μM. The enzyme and compounds were incubated in 5 mM NaCl, 20 mM Tris.HCl pH 8.0, 5 mM DTT for 15 min at 37ºC before starting with the substrate. The emission fluorescence of EDANS was monitored in the following parameters: λ_exc_ = 330 nm, λ_em_ = 490 nm, at 37 °C for 45 min. Fluorescence data (RFU) was converted into substrate cleavage-specific activity using fluorescent conversion factor (FEC) previously calculated based on the EDANS-DABCYL fluorophore pair. Maximum enzyme activity was considered in the situation with vehicle (DMSO), and the values were used to calculate the enzyme inhibition by the compounds. The concentration that inhibits 50% of the enzyme activity (i.e., the half-maximal inhibitory concentration, IC_50_) was calculated in the software GraphPad Prism 9.0.

#### Antiviral and cytotoxicity assays

We state that human participants are not involved in the study. We evaluated the compounds’ biological activity in a cell model using Calu-3 cells, a human submucosal gland cell line. Calu-3 cell models are widely used as a preclinical model for respiratory disease drug screening due to their bronchiolar epithelium characteristics and ability to replicate viruses with higher titles, including SARS-CoV-2^19–22^.

The cytotoxicity assay consisted of interaction between the compounds **7d**, **8e**, and **9g**, at different concentrations (200, 100, 50, 25, and 12.5 µM) with Calu-3 cells (kindly donated by the Farmanguinhos platform RPT11M) at a cell density of 1 × 10^4^ cells/well for 72 h. Afterward, the cells were submitted to viability evaluation by methylene blue assay. For this assay, cells were washed with PBS 1 × and stained with methylene blue solution (Hanks’ solution (HBSS), 1.25% glutaraldehyde, and 0.6% methylene blue) for 1 h. Then, the cells were rewashed and elution solution (50% ethanol, 49% PBS 1x, and 1% acetic acid) was added for 15 min. After that time, the absorbance was read at 660 nm in the spectrophotometer.

The antiviral effect was analyzed by Plaque Forming Units (PFU) assay. For that, Calu-3 cells (1 × 10^4^ cells/well) infected with SARS-CoV-2 B.1 lineage isolate (GenBankMT710714, SisGen AC58AE2) at MOI 0.01 during 1 h at 37 ºC and 5% CO_2_, were treated with the compounds **7d**, **8e**, and **9g**. The treatment was carried out with a semi-log curve of concentration (10, 3.16, 1, 0.316, and 0.1 µM) for 24 h. Then, the supernatants were harvested for virus titer determination. Vero E6 cells (African green monkey kidney, ATCC CRL-1586) in a 96-well plate in 1 × 10^4^ cells/well density were incubated with different dilutions (1:100–1:12,800) of supernatants for 1 h at 37 ºC and 5% CO_2_. After this period, carboxymethylcellulose medium (DMEM-Higher 10x, 2.4% carboxymethylcellulose, and 2% fetal bovine serum) was added to the well at a ratio 1:1, and the cells were cultured for 72 h. Afterward, cells were fixed with formalin 5% for 3 h and posteriorly stained with crystal violet 0.04% for 1 h. The viral titers were determined by PFU/mL.

All the compounds used in vitro assays were resuspended in 100% DMSO (dimethyl sulfoxide), aliquoted, and stored at − 20 °C to avoid compound degradation^23^. The DMSO final concentrations for each molecule’s test concentration were equal or lower than 1% (v/v) diluted in DMEM (Dulbecco’s Modified Eagle Medium) not affecting the growth of the cells^24,25^. According to WHO guidelines^26^, all virus manipulation was realized at a biosafety level 3 (BSL3) multiuser facility.

#### Statistical analysis

The graphs were created using the GraphPad Prism 9.0 software and represent the middle of the results for each experiment realized with a minimum of three technical replicates. We determined the EC_50_ and CC_50_ values by Nonlinear regression of Log(inhibitor) or inhibitor vs. Normalized response of best curve generated (R^2^ values ≥ 0.9).

### Molecular modeling studies

#### Protein and ligand structures preparation

The molecular docking simulations were performed with the crystallographic structure of the SARS-CoV-2 Mpro enzyme obtained in a covalent complex with an irreversible peptide-like inhibitor named **N3** (N-[(5-methylisoxazol-3-yl)carbonyl]alanyl-L-valyl-N ~ 1 ~ -((1*R*,2*Z*)-4-(benzyloxy)-4-oxo-1-{[(3*R*)-2-oxopyrrolidin-3-yl]methyl}but-2-enyl)-L-leucinamide), available in the Protein Data Bank as PDB ID: 6LU7 (resolution = 2.16 Å)^27^. The missing residues were added using the CHARMM-GUI platform (http://www.charmm-gui.org/)^28^, defining the protonation state in physiological pH 7.4, which was predicted by pdb2pqr server (https://server.poissonboltzmann.org/pdb2pqr) and removing water molecules. The three-dimensional structures of the statine-like derivatives **7d**, **8e**, and **9g** were drawn using ChemDraw v. 20.0^29^ considering their protonation state in physiological pH 7.4, and geometry optimization was performed using the MMFF94 force field available in the Spartan (v.10) software (Wavefunction, Inc. https://www.wavefun.com). Then the structures were converted to the pdbqt format using the Open Babel chemical toolbox^30^.

#### Molecular docking

The molecular docking simulations were performed using the AutoDock Vina 1.1.2 program^31^ and prepared in the AutoDockTools (ADT) (v.1.5.6)^32^ graphical interface according to the protocol and parameters previously described by our research group^33^, considering the physiological pH 7.4. The docking protocol was validated by redocking the **N3** inhibitor as a non-covalent ligand. The **N3** inhibitor was removed from the structure, and the binding orders were restored for the inhibitor interacting with Cys145 amino acid. The root-mean-square deviations (RMSD) calculations of the 10 pose results were carried out using the PyMOL (v. 3.5) software^34^, considering the best results RMSD < 2.0 Å. Ligand-Mpro complexes were analyzed for the main intermolecular interactions, such as hydrogen bond (H-bond) and hydrophobic interactions, with the PyMOL, and the images of the binding poses were composed with the Visual Molecular Dynamics (VMD) (v. 1.9.4) software^35^.

#### Molecular dynamics

Molecular dynamics simulations were carried out in triplicate with the GROMACS 2022 package^36^ using the CHARMM36 force field^37^ with the top-ranked pose of each ligand-Mpro complex obtained by molecular docking applying the protocol described previously by our research group^33^. The ionization states of the protein's residues were adjusted to pH 7.4 using the pdb2gmx Python script. Ligand–protein complex was included in a periodic triclinic box (box dimensions: 5.416 × 4.538 × 4.348 nm and box volume: 854.88 nm^3^), solvated with the TIP3P model of water, and neutralized with 8 atoms of Na^+^ ions to neutral ligands (**7d** and **8e**) and 7 Na^+^ atoms to charged ligand (**9g**).

RMSD, RMSF, hydrogen bonding, and cluster analysis were made using gmx rms, gmx rmsf, gmx hbond, and gmx cluster modules available in the GROMACS package. The ΔG_bind_ was calculated by the MM-PBSA method, applying g_MMPBSA module v.5.1.2^38^ considering the internal dielectric of the protein solute of 2. The energy contribution of residues was calculated with MmPbSaStat.py and MmPbSaDecomp.py scripts^38^. H-bonding frequencies were calculated with HbMap2Grace^39^ software. Figures of the interactions and trajectories analysis were composed with the VMD^35^ software.

## Results and discussion

### Chemistry

**Figure 1:**
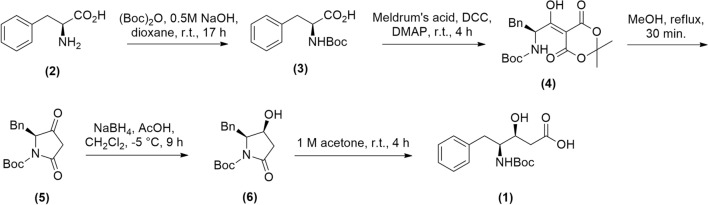
Synthesis of the β-hydroxy-γ-amino acid intermediate (**1**).

The statine-like core (AHPPA) contained in the final products was obtained by the stereoselective synthesis of the β-hydroxy-γ-amino acid **1** synthesized from *L*-phenylalanine **2** (Fig. 1)^40^. The protected phenylalanine amino acid **3** and the freshly prepared Meldrum’s acid were condensed to afford **4** and then it was refluxed in methanol affording the tetramic acid **5**. The β-hydroxypyrrolidinone **6** was obtained from diastereoselective reduction of **5** using NaBH_4_ and used as starting material to obtain the β-hydroxy-γ-amino acid intermediate (*N*-Boc-(3*S*,4*S*)-AHPPA) **1**^40–42^.

**Figure 2:**
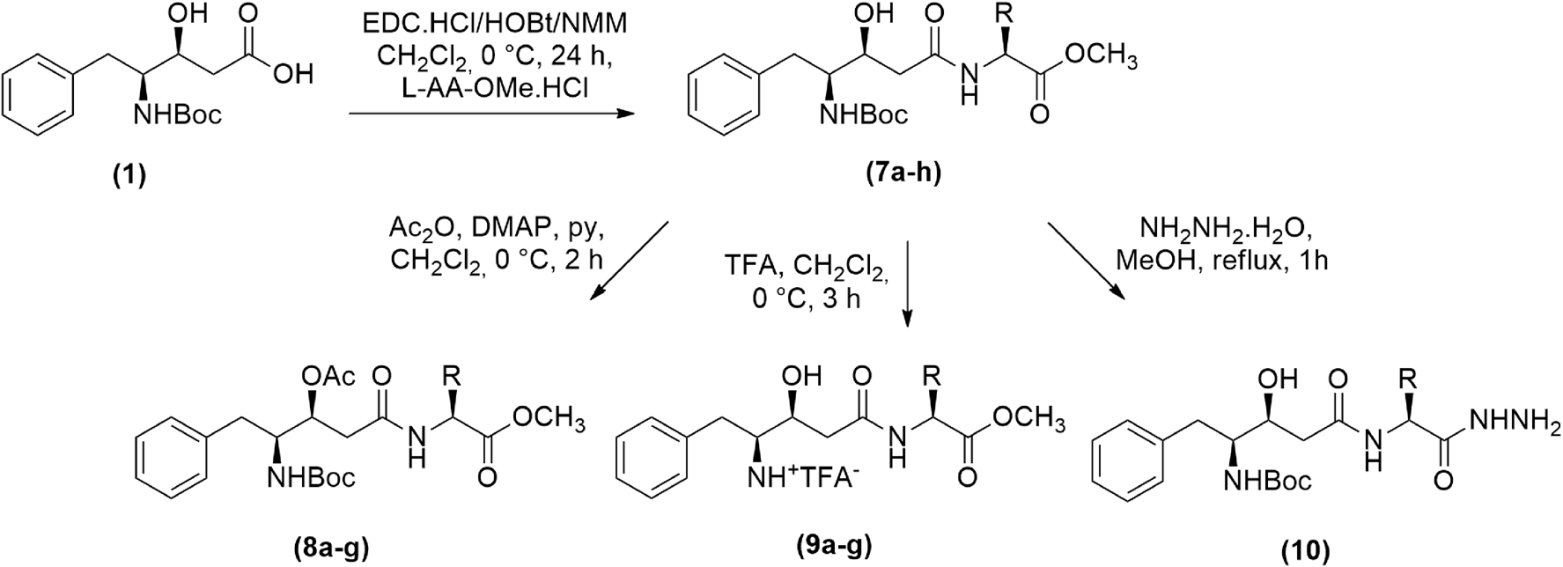
Synthesis of the statine-based peptidomimetics (**7a-h**, **8a-g**, **9a-g**, and **10**).

The ester series of peptidomimetics **7a-h** (Table 1) was obtained by coupling reaction of statine-like compound **1** and several amino methyl ester hydrochlorides from the following natural amino acids *L*-serine (*L*-Ser-OMe.HCl), *L*-tyrosine (*L*-Tyr-OMe.HCl), *L*-threonine (*L*-Thr-OMe.HCl), *L*-isoserine (*L*-Isoser-OMe.HCl), *L*-leucine (*L*-Leu-OMe.HCl), *L*-phenylalanine (*L*-Phe-OMe.HCl), *L*-proline (*L*-Pro-OMe.HCl) and *L*-valine (*L*-Val-OMe.HCl)^43^, by employing the classical *N*-(3-dimethylaminopropyl) *N*'-ethylcarbodiimide/1-hydroxy-benzotriazole/ *N*-methylmorpholine (EDC/HOBt/NMM) protocol (Fig. 2)^18,44^.

**Table 1:** General structure of the synthesized statine-based peptidomimetics (**7a-h**, **8a-g**, **9a-g** and **10**) and their respective substituents (R, R_1_, R_2_, and R_3_).


Compound	R	R_1_	R_2_	R_3_
7a	–CH_2_OH	–H	–NHBoc	–OCH_3_
7b	–CH_2_Ph-4-OH	–H	–NHBoc	–OCH_3_
7c	–CH(OH)CH_3_	–H	–NHBoc	–OCH_3_
7d	–CH_2_CH(OH)–	–H	–NHBoc	–OCH_3_
7e	–CH_2_CH(CH_3_)_2_	–H	–NHBoc	–OCH_3_
7f.	–CH_2_Ph	–H	–NHBoc	–OCH_3_
7g	–(CH_2_)_3_–	–H	–NHBoc	–OCH_3_
7 h	–CH(CH_3_)_2_	–H	–NHBoc	–OCH_3_
8a	–CH_2_OAc	–Ac	–NHBoc	–OCH_3_
8b	–CH_2_Ph-4-OAc	–Ac	–NHBoc	–OCH_3_
8c	–CH(OAc)CH_3_	–Ac	–NHBoc	–OCH_3_
8d	–CH_2_CH(CH_3_)_2_	–Ac	–NHBoc	–OCH_3_
8e	–CH_2_Ph	–Ac	–NHBoc	–OCH_3_
8f.	–(CH_2_)_3_–	–Ac	–NHBoc	–OCH_3_
8g	–CH(CH_3_)_2_	–Ac	–NHBoc	–OCH_3_
9a	–CH_2_OH	–H	–NH_3_^+^	–OCH_3_
9b	–CH_2_Ph-4-OH	–H	–NH_3_^+^	–OCH_3_
9c	–CH(OH)CH_3_	–H	–NH_3_^+^	–OCH_3_
9d	–CH_2_CH(CH_3_)_2_	–H	–NH_3_^+^	–OCH_3_
9e	–CH_2_Ph	–H	–NH_3_^+^	–OCH_3_
9f.	–(CH_2_)_3_–	–H	–NH_3_^+^	–OCH_3_
9g	–CH(CH_3_)_2_	–H	–NH_3_^+^	–OCH_3_
10	–CH_2_Ph	–H	–NHBoc	–NHNH_2_

The acetylation reactions were carried out using acetic anhydride and DMAP in a basic medium affording the final compounds **8a-g**^45^. The *N*-Boc deprotection of **7a-h** compounds was made using trifluoroacetic acid generating peptidomimetics **9a-g**^46^. Finally, the hydrazide compound **10** was obtained from the corresponding ester **7 h** (R = –CH_2_Ph) and hydrazine hydrate in methanol (Fig. 2).

### Biological assays

#### Inhibition of SARS-CoV-2 Mpro

The ability of compounds to inhibit the SARS-CoV-2 Mpro activity was assessed by an in vitro FRET-based assay. Table 2 shows % of maximum inhibition (efficacy) compared to negative control (vehicle, DMSO) and IC_50_ (potency) values. The most promising compounds were selected based on efficacy (high maximum inhibition values) and potency (low half maximum inhibition, IC_50_ values). As a positive control, we used the **GC-376** (PubChem CID 71,481,119) compound, a small synthetic dipeptide previously identified as a covalent inhibitor of SARS-CoV’s Mpro and used in several studies as gold standard for in vitro Mpro inhibition assay^47,48^. In our system, the maximum inhibition achieved by **GC-376** was 75%, presenting an IC_50_ = 0.541 µM (Table 2). Sixteen out of 19 tested compounds inhibited Mpro activity by 50% or more (Table 2) and maximum inhibition was achieved with compound **9g**, which inhibited the enzyme activity by 80% compared to negative control (vehicle).

**Table 2:** Inhibition of SARS-CoV-2 Mpro proteolytic activity by the statine-based peptidomimetics.

Compound	Maximum inhibition (%)	IC_50_ (µM)
7a	70	> 1000
7b	70	> 1000
7c	< 50	ND
7d	71	0.924
7e	< 50	ND
7f.	NT	ND
7g	NT	ND
7 h	< 50	ND
8a	50	1000
8b	65	> 1000
8c	60	2.449
8d	60	0.391
8e	70	0.606
8f.	NT	ND
8g	66	> 1000
9a	57	> 1000
9b	52	> 1000
9c	NT	ND
9d	58	777.8
9e	51	> 1000
9f.	68	> 1000
9g	80	0.617
10	61	> 1000
GC-376	75	0.541

*NT* not tested, *ND* not determined.

Only five compounds (**7d**, **8c**, **8d**, **8e**, and **9g**) were able to inhibit Mpro activity by 60% or more at low IC_50_ values (Table 2), while all the other compounds only exerted inhibitory effects (≥ 50%) at high IC_50_ values (IC_50_ > 770 μM). Therefore, we selected the three most promising compounds (**7d**, **8e**, and **9g**), which showed 70% or more inhibition than standard **GC-376** and had an IC_50_ of less than 1 µM (Table 2). These compounds were further evaluated for their ability to inhibit virus replication.

#### Inhibition of SARS-CoV-2 virus replication

**Figure 3:**
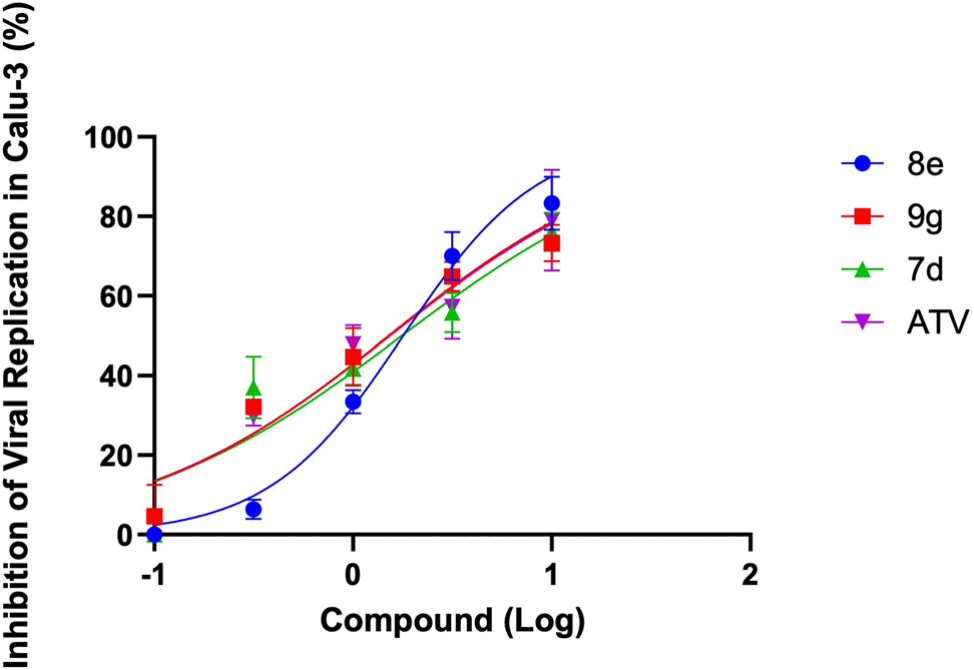
The dose-dependent curves of the statine-based peptidomimetics **7d**, **8e**, and **9g** (and atazanavir, ATV, as positive control) against SARS-CoV-2. Calu-3 cells infected with SARS-CoV-2 MOI 0.01 were treated with the compounds **7d**, **8e**, **9g**, and ATV at a semi-log curve of concentration (10, 3.16, 1, 0.316, and 0.1 µM) for 24 h at 37 ºC, 5% CO_2_ (n = 3).

The compounds **7d, 8e** and **9g** showed high efficacy, inhibiting around 80% of SARS-CoV-2 replication on Calu-3 cells at higher concentrations tested (Fig. 3). The EC_50_ values of these compounds (**7d**, EC_50_ = 1.76 μM; **8e**, EC_50_ = 1.79 μM; and **9g**, EC_50_ = 1.51 μM) are equivalent to atazanavir (ATV, EC_50_ = 1.53 μM), an azapeptide antiretroviral drug approved for treating HIV (human immunodeficiency virus) patients (Table 3). Atazanavir is a HIV-1 aspartic protease inhibitor that can inhibit the SARS-CoV-2 Mpro enzyme, hampering therefore, the nsps processing and, consequently, the virus replication^49,50^. In addition, the compounds also presented a potent effect when compared with molnupiravir (estimated EC_50_ of 1.97 µM), an antiviral drug authorized by Food Drug Administration (FDA) under an emergency use for COVID-19 treatment; and more potent than lopinavir/ritonavir combination (EC_50_ = 8.2 µM), proposed as a treatment for COVID-19 during 2020^51,52^.

**Table 3:** The EC_50_, CC_50_, and SI values of statine-based peptidomimetics (**7d**, **8e**, and **9g**) and atazanavir (ATV) as positive control in Calu-3 cells. EC_50_ – Compound concentration necessary to obtain 50% of its effective inhibitory activity; CC_50_—Compound concentration required to reduce 50% of cell viability; SI – Selective Index (CC_50_/EC_50_).

Compound	EC_50_ (µM)	CC_50_ (µM)	SI
_7d_	1.76 ± 0.3	> 200	> 113.6
_8e_	1.79 ± 0.1	> 200	> 111.7
_9g_	1.51 ± 0.2	> 200	> 132.4
_ATV_	1.53 ± 0.3	> 200	> 130.7

Furthermore, none of the compounds showed toxicity in Calu-3 cells, and their CC_50_ values exceeded the highest concentration tested in the experiment (Table 3). The absence of toxicity (CC_50_ > 200 µM) and the lower EC_50_ make the compounds biological effect specific to the virus with SI (selectivity index) values more than 100 (Table 3). Thus, the compounds **7d**, **8e**, and **9g** are promising bioactive molecules for inhibiting SARS-CoV-2 replication.

### In silico molecular docking and dynamics simulations

The potential binding mode and main intermolecular interactions of the statine-based derivatives **7d**, **8e**, and **9g** into the active site of the SARS-CoV-2 Mpro were evaluated through molecular docking following the protocol previously reported by our research group^33^. The docking protocol used in our study was validated by redocking, considering the **N3** inhibitor as a non-covalent inhibitor. The bond between **N3** and the enzyme was broken, and its double bond was restored. Our analysis considered 14 rotatable bonds, as the **N3** has 4 amide bonds. After executing the protocol, we calculated the RMSD value, which was 1.91 Å (Figure S2), in comparison to the structure available in the crystal. According to the molecular docking simulations with compounds **7d**, **8e**, and **9g**, it was observed that statine-based peptidomimetics could interact with the Mpro enzyme at the substrate binding site by hydrogen bond (H-bond) and hydrophobic interactions. Mpro has several subsites (“S”) for substrate binding^53^, which are identified by the Schechter & Berger (1967) nomenclature (e.g., S5, S4, S3, S2, S1, S1′, S2′, S3′, S4′, S5′), including **S5** (Pro168, Thr190, and Ala191), **S4** (Leu167, Phe185, Gln189, and Gln192), **S2** (His41, Met49, Tyr54, Met165, and Asp187), **S1** (Phe140, Leu141, Asn142, Ser144, His163, Glu166, and His172), and **S1’** (Thr24 and Thr25)^53^.

**Figure 4:**
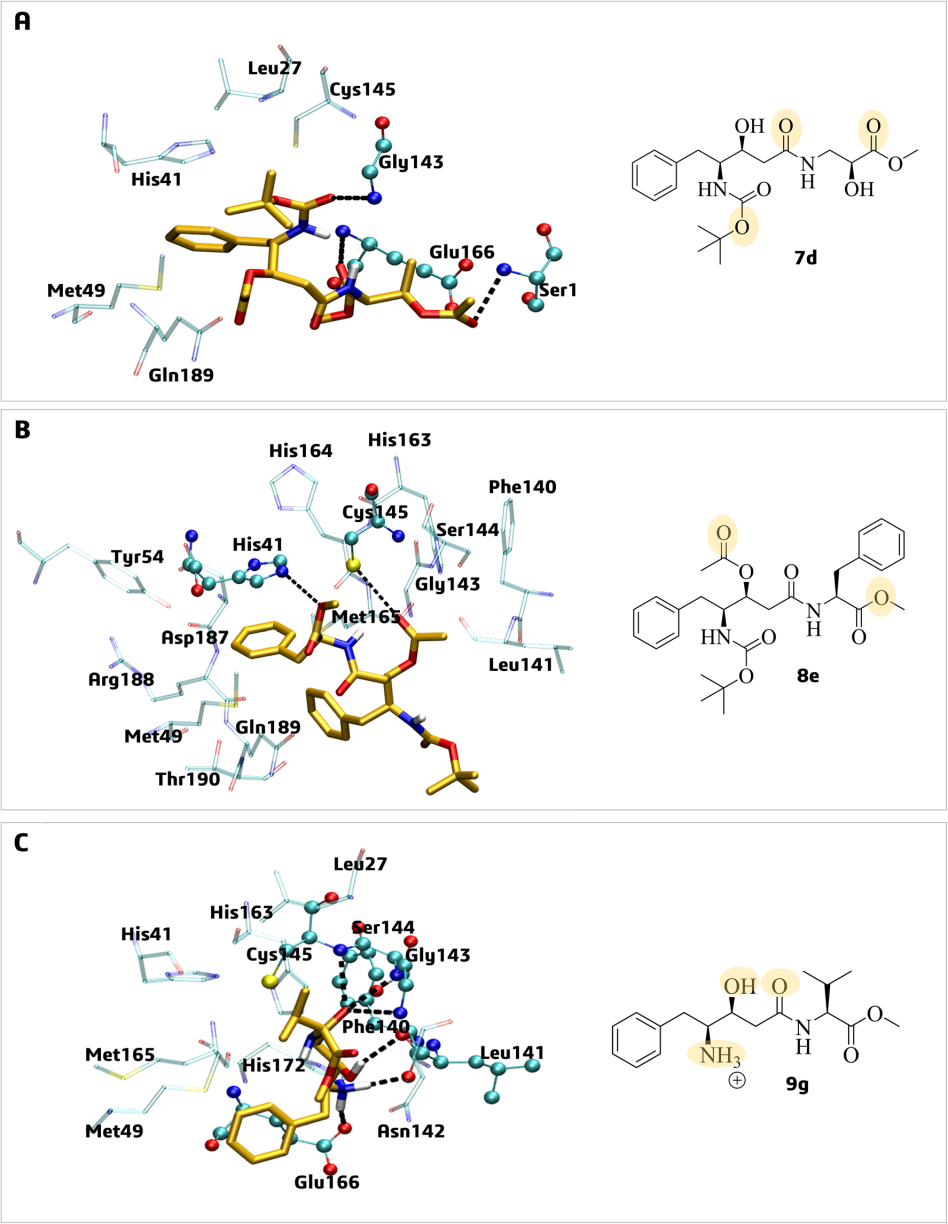
Best pose by molecular docking simulations of statine-like derivatives on the SARS-CoV-2 Mpro active site (PDB ID: 6LU7): (**A**) **7d**; (**B**) **8e**, and (**C**) **9g**. The residues involved in H-bond interactions (dashed black lines) with the ligands are in ball-and-line model (cyan color) and residues involved in hydrophobic interactions are in stick models (light green color). In 2D structures, the atoms (or groups) of the ligands involved in H-bond interactions are circled in yellow.

Specifically, the top-ranked docking poses of the statine-based derivatives **7d**, **8e**, and **9g** in general shows H-bond and hydrophobic interactions with residues of the catalytic dyad (Cys145 and His41)^27^ and the **S1** subsite (Leu141, Asn142, and Glu166) (Fig. 4a–c).

The pose of the statine-like **7d** shows putative H-bond interactions with Gly143 and catalytic Cys145 (these two residues constitute the oxyanion hole)^54^, and in addition hydrophobic interactions with amino acids residues from three subsites: **S1** (Asn142, Leu141, Phe140, Glu166, and His163), **S2** (His41 and Met165), and **S4** (Gln189) (Fig. 4a).

The pose of **8e** shows putative hydrophobic interactions with residues from all subsites of Mpro, particularly **S1** (Phe140, Leu141, and His163), **S2** (Met49, Tyr54, Met165, and Asp187), **S4** (Gln189), and **S5** (Thr190). It also presented H-bond interactions with catalytic dyad His41-Cys145 (Fig. 4b). Finally, **9g** pose shows putative H-bond interactions with four residues (Phe140, Gly143, Ser144, and Cys145) and hydrophobic interactions with residues of subsites **S1** (Leu141, Asn142, His163, and Glu166) and **S2** (His41, Met49, and Met165) (Fig. 4c).

It is worth mentioning that the Mpro (PDB ID: 6LU7) co-crystalized inhibitor, **N3**, is an irreversible peptide-like inhibitor^27^. According to its redocking pose, **N3** shows similar H-bonding interactions with residues of subsites **S1** (Phe140), **S2** (His41 and Glu166), and **S4** (Gln189) (Figure S1), as seen in the statine-like derivatives proposed as non-covalent inhibitors, that shared similar binding mode, at least with two subsites as this inhibitor.

The molecular dynamics simulations (MD) were carried out in triplicate, starting with the top-ranked poses of **7d**, **8e**, and **9g** with SARS-CoV-2 Mpro (PDB ID: 6LU7), were performed to evaluate the behavior of these ligand–protein complexes in an aqueous system, during 200 ns, using the GROMACS software^55^ with Charmm36 force field^37^.

In the first instance, the compounds were docked into the Mpro’s active site, and as mentioned before, they remained close to the Cys145-His41 catalytic dyad region. To confirm if this specific area would encourage favorable and persistent interactions, we conducted an RMSD (root-mean-square deviations) analysis of the ligands over a 200 ns simulation period.

**Figure 5:**
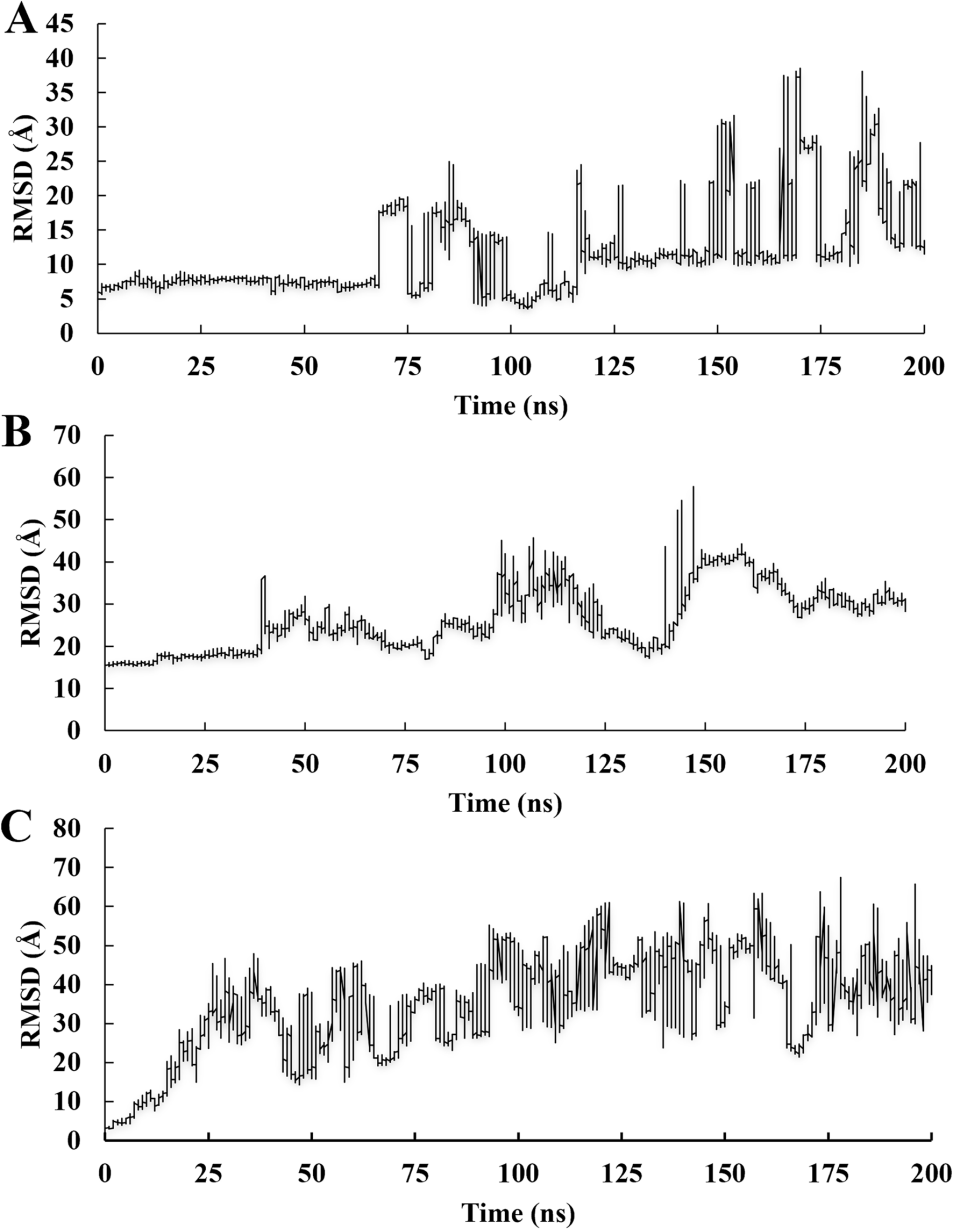
RMSD analysis of the **7d** (**A**), **8e** (**B**), and **9g** (**C**) relative to the Mpro-Cα atoms.

The RMSD analysis for compound **7d** showed a tendency to leave the active site after 70 ns of simulation, presenting an RMSD value of 11.6 ± 6.40 Å and a high standard deviation (Fig. 5a). Compound **8e** presented relative stability and persistence into the active site at the beginning of the simulation (0–40 ns); after that, left the active site presenting an RMSD value of 26.3 ± 7.34 Å (Fig. 5b). The derivative **9g** showed RMSD = 34.7 ± 12.8 Å, with low persistence in the active site (about 15 ns). Based on the 200 ns MDS analysis, it can be stated that inhibitor **7d** has the strongest interaction with the protein’s active site. While the other inhibitors may exhibit good inhibition values, they cannot remain in contact with this region for an extended period.

**Figure 6:**
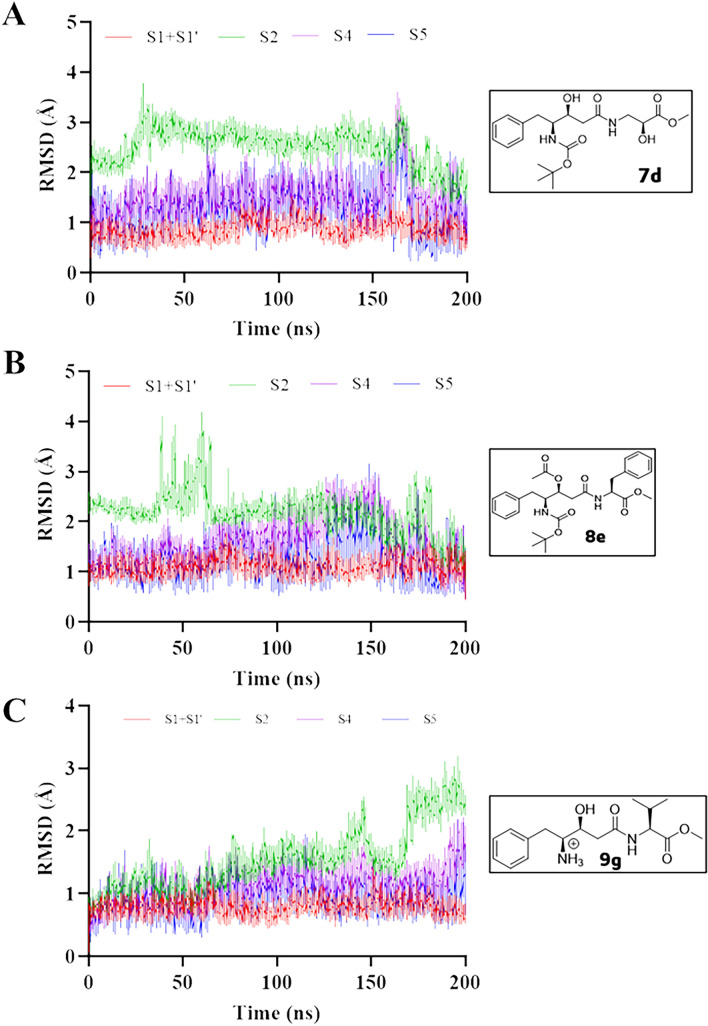
Cα-RMSD analysis per Mpro subsites **S1** + **S1’** (red line), **S2** (green line), **S4** (purple line), and **S5** (blue line) that are relative to the simulations by molecular dynamics of the ligand-Mpro complex. Ligands: **7d** (**A**), **8e** (**B**), and **9g** (**C**).

Since Mpro is reported to have several subsites for substrate binding^53^, we evaluated the RMSD profiles of the Cα atoms per the subsites. The RMSD obtained from Cα atoms of the **7d-Mpro** complex showed instability of Cα atoms mainly to subsites **S2**, **S4**, and **S5** with RMSD values of 2.52 ± 0.35 Å, 1.48 ± 0.36 Å, and 1.30 ± 0.40 Å, respectively, showing more significant variations for **S4** and **S5**, even with low mean values. Moreover, it presented stability to **S1** + **S1’** subsites (0.85 ± 0.16 Å) for all MDs (Fig. 6a). On the other hand, **8e-Mpro** complex for subsites **S1 + S1’** (1.09 ± 0.14 Å), **S4** (1.62 ± 0.42 Å), and **S5** (1.28 ± 0.38 Å) showed stability due to a minimal variation in standard deviation (sd) values < 1.0 Å^56^. At the same time, **S2** presented the high mean (2.16 ± 0.40 Å) (Fig. 6b). Although the mean and sd values observed are low, there appears to be some connection between the movements of the S2 and S4 subsites that affect the tendency for the ligand to escape from the binding site. Notably, subsites S2 and S4 contain mainly hydrophobic residues that can interact with the phenyl groups of the ligand in these regions.

Finally, **9g-Mpro** complex showed instability of Cα atoms to **S2** subsite from 150 ns of MD simulation presenting RMSD = 1.54 ± 0.50 Å, while presenting stability for other subsites with 0.78 ± 0.11 Å (**S1** + **S1’**), 1.13 ± 0.24 Å (**S4**), and 0.95 ± 0.21 Å (**S5**) for all period (Fig. 6c).

**Figure 7:**
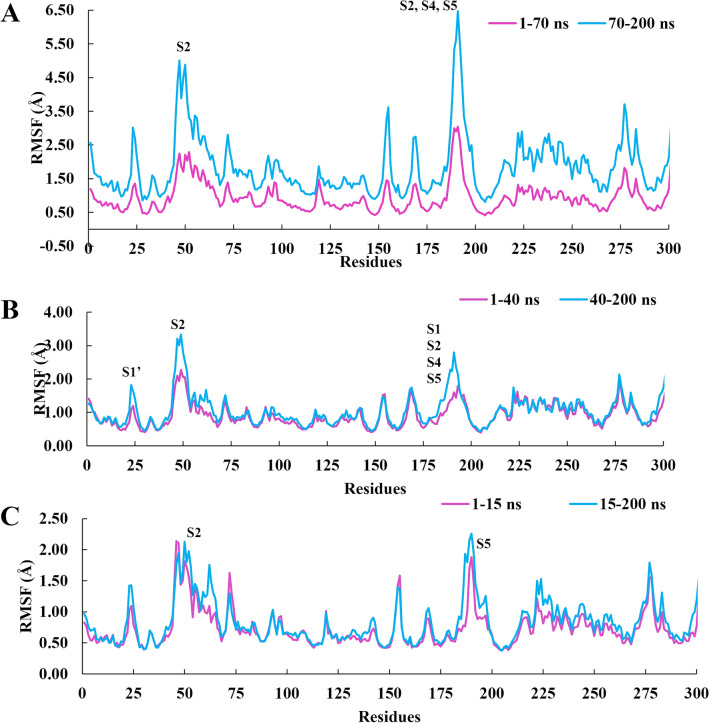
Cα-RMSF analysis at different times relative to the simulations by molecular dynamics of the ligand-Mpro complex. Ligands: **7d** (**A**), **8e** (**B**) and **9g** (**C**).

It is interesting to note that the root-mean-square-fluctuation (RMSF) calculation for **7d** indicates that, even when this ligand is inside and out of the binding site cavity, the residues have mobility greater than 2.0 Å for Cα atoms of Asp187(**S2**), Gln189(**S4**), and Thr190(**S5**) (Fig. 7a). Considering the movement **8e** during the 200 ns of simulation, we evaluated the difference in the RMSF of the Cα atoms in two time intervals: 1–40 ns and 40–200 ns (Fig. 7b). In general, the ligand induces a gain of 0.98 to 2.10 Å in the mobility of residues belonging to **S1**, **S2**, **S4**, and **S5** subsites, especially for: (i) Thr24(**S1’**) and Thre25(**S1’**) (RMSF = 0.98 Å); (ii) Met49(**S2**) (RMSF = 2.10 Å); (iii) Glu166(**S1**), His172(**S1**), Met165(**S2**), Leu167(**S4**), and Pro168(**S5**) (RMSF = 1.49 Å); (iv) Asp187(**S2**), Gln189(**S4**), Thr190(**S5**), and Ala191(**S5**) (RMSF = 1.58 Å). The active site of Mpro is formed by catalytic dyad Cys145 and His41 and other residues, such as Leu141, Asn142, Pro168, Thr190, and Ala191^57^, which the last two presented fluctuations in the RMSF results. In addition, Glu166(**S1**) plays a critical role in establishing the interaction point between the two monomers of the Mpro^58^.

Regarding the statine-like derivative **9g**, the RMSF values were analyzed considering two-time intervals (1–15 ns, 15–200 ns) that indicated fluctuations above 2 Å for residues belonging to subsites **S2** (His41, Tyr54, and Met49) and **S5** (Thr190) predominantly (Fig. 7c).

The analysis of intermolecular interactions via hydrogen bonding of ligand **7d** exhibits a hydrogen bonding interaction with the residue Glu166(**S1**), His163(**S1**), and Ser144, with a great lifetime observed between 18 and 30%. In addition to H-bond interactions with catalytic residues Cys145 and His41 with low persistence, 5.19 and 8.97 respectively (Table 4).

**Table 4:** Donor and acceptor atoms from ligands **7d**, **8e**, and **9g** involving interactions with respective amino acids and their lifetime (in percentage).

Ligand	Donor	Acceptor	Lifetime (%)
	Lig-N	Cys145-N	5.19
	Thr304-O	Lig-O	6.79
	Lig-O	Glu166-N	7.00
	Lig-O	Ser144-N	7.47
	Asn142-N	Lig-O	7.66
	His41-N	Lig-O	8.36
	Lig-N	His41-N	8.97
	Lig-N	Glu166-N	12.0
	Glu166-N	Lig-O	18.9
	Lig-N	Glu166-O	22.3
	Lig-O	His163-N	23.6
	Lig-N	Glu166-O	27.4
	Lig-O	Ser144-N	30.0
	Lig-O	Glu166-O	30.3
	Lig-O	Glu166-O	30.4
	Lig-N	Gln189-O	6.26
	Gln189-N	Lig-N	7.93
	Gln189-N	Lig-O	15.0
	Glu166-N	Lig-O	13.5
	Glu166-N	Lig-O	43.8
	Lig-N	Ser46-O	5.07
	Lig-O	Asp248-O	5.63
	Lig-N	Gln189-O	5.98
	Lig-N	Asp245-O	7.68
	Lig-O	Glu166-N	8.12
	Lig-N	Asp245-O	8.38
	Lig-N	Asp248-O	9.51
	Lig-O	Asp248-O	11.2
	Lig-N	Gln306-O	13.9
	Lig-N	Gln306-O	15.9

The analysis of the **8e-Mpro** complex revealed those with the longest duration involving the ligand and the following residues: Glu166(**S1**) (43.8%) and Gln189(**S4)** with short lifetime from 6.26 to 15% (Table 4). Furthermore, FDA-approved Paxlovid™ (nirmatrelvir + ritonavir) was the first oral antiviral for mild to moderate COVID-19 cases in adults on May 25, 2023^59^. Nirmatrelvir inhibits viral replication by bonding to Cys145 catalytic residue from Mpro and forming hydrogen bonds with catalytic His164, Glu166(**S1**), and Gln189(**S4**)^60^. This finding supported the acetylated statine-like derivative **8e**, which demonstrates the potential of binding with at least two subsites, which is important to maintain the ligand in the active site, especially for proteases as Mpro^53^, highlighting these statine-based peptidomimetics to act as promising inhibitors.

Finally, for **9g** the most considerable hydrogen bonds observed presented a short lifetime with Glu166(**S1**) (8%), Asp245 and 248 (5.63–11.2%), Gln306 (13–15%), and Ser46 (5%) (Table 4).

In contrast to the docking results, the molecular dynamics simulations revealed that statine-like derivatives, like **8e** and **7d**, with large and hydrophobic substituents, including phenyl and *N*-Boc groups, had a greater influence on the number of interactions with amino acids belonging to at least two subsites due to their increased hydrophobicity. On the other hand, **9g**, which has weaker bonds, had less impact on the interactions.

Finally, since the RMSD analysis of the statine-like ligands indicated two constant regions before and after leaving the center of the active site, we calculated the binding free energy (ΔG_bind_) for both periods of time (Table 5). Considering the period during which the ligands remained bound to the enzyme active site, it is more evident that statine-like derivative **9g** is promising for inhibition of Mpro since it presented the best ΔG_bind_ value of − 57.7 kcal/mol, while statine-based derivative **7d** showed a lower ΔG_bind_ value of − 37.8 kcal/mol and statine **8e** almost twice less energy value observed for **9g** with ΔG_bind_ = − 31.1 kcal/mol (Table 5).

**Table 5:** The binding free energy (ΔG_bind_) terms of the ligand-Mpro complexes calculated for **7d**, **8e**, and **9 g** with the MM-PBSA method (mean ± standard deviation energies; kcal/mol): van der Waals (ΔE_vdW_), electrostatic (ΔE_elect_), solvation (ΔE_solv_), and solvent accessible surface area (ΔE_sasa_).


Time (ns)	ΔE_vdw_	ΔE_elect_	ΔE_solv_	ΔE_sasa_	ΔG_bind_
1–70	− 35.4 ± 4.93	− 16.7 ± 12.6	18.8 ± 11.4	− 4.57 ± 0.41	− 37.8 ± 22.6
70–200	− 26.1 ± 22.8	− 8.01 ± 9.72	7.88 ± 5.29	− 3.13 ± 2.60	− 29.3 ± 28.4


Time (ns)	ΔE_vdw_	ΔE_elect_	ΔE_solv_	ΔE_sasa_	ΔG_bind_
1–48	− 35.6 ± 3.10	− 6.90 ± 2.00	15.9 ± 14.1	− 4.60 ± 0.20	− 31.1 ± 9.80
48–200	− 33.6 ± 1.60	− 5.60 ± 1.50	12.7 ± 11.0	− 4.20 ± 0.40	− 30.6 ± 10.5


Time (ns)	ΔE_vdw_	ΔE_elect_	ΔE_solv_	ΔE_sasa_	ΔG_bind_
1–15	− 20.2 ± 4.60	− 78.5 ± 22.2	44.5 ± 41.6	− 3.24 ± 0.51	− 57.7 ± 14.8
15–200	− 4.21 ± 7.27	− 60.7 ± 73.0	24.4 ± 28.4	− 0.96 ± 1.62	− 41.5 ± 55.2

Based on our previous discussions, the binding free energy values also indicate the favorable hydrophobicity contribution of these statine-based compounds, probably due the hydrophobic characteristic of several residues on the Mpro subsites, such as **S1** (Phe140, Leu141), **S2** (Met49, Tyr54, Met165), **S4** (Leu167, Phe185), and **S5** (Ala191) ^53^. As a result, the **8e** and **7d** derivatives with multiple hydrophobic substituents (i.e., two phenyl rings, an acetyl, and an *N*-Boc group) have stronger interactions with these pockets. Finally, the **9g** derivative has the most hydrophilic groups (i.e., a positive charged amine group and a free hydroxyl group) in the evaluated series, resulting in the highest energy cost of desolvation of the binding site (44.5 kcal/mol) when compared to the other compounds (~ 20 kcal/mol) (Table 5).

It was noted that ΔG_bind_ energy analysis of the ligands before moving out from the binding site was consistent, as previously discussed. Although derivative **9g** remained in the active site for less time than **8e** and **7d**, this interaction was sufficient to cause inhibition of the enzyme, which resulted in its best binding free energy value of − 57.7 kcal/mol observed (Table 5).

## Conclusions

Targeting the SARS-CoV-2 main protease (Mpro), 23 statine-based peptidomimetics were synthesized and tested for their ability to inhibit the Mpro activity. The three most effective compounds (**7d**, **8e**, and **9g**) could inhibit the Mpro enzyme activity in the sub-micromolar range. These compounds have been found to be non-cytotoxic and can suppress about 80% of the replication of the SARS-CoV-2 virus. In silico studies have also shown that these compounds are stable and have persistent interactions with the Mpro active site, indicating their potential as inhibitors. By blocking the activity of the main protease, which is essential for viral replication, these compounds have the potential to inhibit virus replication with low micromolar EC_50_. Finally, we found new hit compounds that could lead to promising drug candidates against the COVID-19 disease.

### Supplementary Information

Supplementary Information.

## Data Availability

The authors confirm all data generated and analyzed during this study are available in the article and in the supplementary information.
