# Supplementary material for: Statine-based peptidomimetic compounds as inhibitors for SARS-CoV-2 main protease (SARS-CoV‑2 Mpro)
Source: Sci Rep. 2024 Apr 18;14:8991. doi: 10.1038/s41598-024-59442-4 (PMC11026380; doi:10.1038/s41598-024-59442-4)
Supplement: Supplementary file 1 — Supplementary Information. [file 41598_2024_59442_MOESM1_ESM.docx]

**Statine-based Peptidomimetic Compounds as Inhibitors for SARS-CoV-2 Main Protease (SARS-CoV‑2 Mpro)**

Pedro Henrique R. de A. Azevedo^1^, Priscila G. Camargo^2^, Larissa E. C. Constant^3^, Stephany da S. Costa^3^, Celimar Sinézia Silva^3^, Alice S. Rosa^4,5^, Daniel D. C. Souza^4,5^, Amanda R. Tucci^4,5^, Vivian N. S. Ferreira^4^, Thamara Kelcya F. Oliveira^4,5^, Nathalia R. R. Borba^4^, Carlos R. Rodrigues^2^, Magaly G. Albuquerque^6^, Luiza R. S. Dias^1^, Rafael Garrett^6^, Milene D. Miranda^4,5^, Diego Allonso^3,7^, Camilo Henrique da S. Lima^6*^, Estela Maris F. Muri^1*^

*^1^Laboratório de Química Medicinal, Faculdade de Farmácia, Universidade Federal Fluminense, Niterói, RJ, 24241-000, Brazil*

*^b^Universidade Federal do Rio de Janeiro, Faculdade de Farmácia, Rio de Janeiro, RJ, 21941-853, Brazil*

*^3^Laboratório de Biotecnologia e Bioengenharia Tecidual, Instituto de Biofísica Carlos Chagas Filho, Universidade Federal do Rio de Janeiro, Rio de Janeiro, RJ, 21941-853, Brazil*

*^4^Laboratório de Morfologia e Morfogênese Viral, Instituto Oswaldo Cruz, Fundação Oswaldo Cruz, Rio de Janeiro, RJ, 21040-900, Brazil*

*^5^Programa de pós-graduação em Biologia Celular e Molecular, Instituto Oswaldo Cruz, Fundação Oswaldo Cruz, Rio de Janeiro, RJ, 21040-900, Brazil*

*^6^Universidade Federal do Rio de Janeiro, Instituto de Química, Programa de Pós-Graduação em Química, Rio de Janeiro, RJ, 21941-853, Brazil*

*^7^Departamento de Biotecnologia Farmacêutica, Faculdade de Farmácia, Universidade Federal do Rio de Janeiro, Rio de Janeiro, RJ, 21941-853, Brazil*

**Supplementary information**

**Table of contents**

[**Characterization and yields of the synthetic peptidomimetic compounds 7a-h** 3](#_Toc160150911)

[**Characterization and yields of the synthetic peptidomimetic compounds 8a-g** 5](#_Toc160150912)

[**Characterization and yields of the synthetic peptidomimetic compounds 9a-g** 8](#_Toc160150913)

[**Figure S1.** N3 hydrogen bonds in the active site of Mpro. 11](#_Toc160150914)

[**Figure S2.** Best pose of redocking result for ligand N3 (in blue) compared to original one (in green). 11](#_Toc160150915)

[**Spectra of compound 7a**. 12](#_Toc160150916)

[**Spectra of compound 7b**. 16](#_Toc160150917)

[**Spectra of compound 7c**. 20](#_Toc160150918)

[**Spectra of compound 7d**. 24](#_Toc160150919)

[**Spectra of compound 7e**. 29](#_Toc160150920)

[**Spectra of compound 7f**. 33](#_Toc160150921)

[**Spectra of compound 7g**. 37](#_Toc160150922)

[**Spectra of compound 7h**. 41](#_Toc160150923)

[**Spectra of compound 8a**. 45](#_Toc160150924)

[**Spectra of compound 8b**. 49](#_Toc160150925)

[**Spectra of compound 8c**. 53](#_Toc160150926)

[**Spectra of compound 8e**. 61](#_Toc160150927)

[**Spectra of compound 8f**. 65](#_Toc160150928)

[**Spectra of compound 8g**. 69](#_Toc160150929)

[**Spectra of compound 9a**. 73](#_Toc160150930)

[**Spectra of compound 9b**. 76](#_Toc160150931)

[**Spectra of compound 9c**. 80](#_Toc160150932)

[**Spectra of compound 9d**. 84](#_Toc160150933)

[**Spectra of compound 9e**. 88](#_Toc160150934)

[**Spectra of compound 9f**. 92](#_Toc160150935)

[**Spectra of compound 9g**. 96](#_Toc160150936)

[**Spectra of compound 10**. 100](#_Toc160150937)

# **Characterization and yields of the synthetic peptidomimetic compounds 7a-h**

*(S)-methyl-2-((3S,4S)-4-((tert-butoxycarbonyl)amino)-3-hydroxy-5- phenylpentanamido)-3-hydroxypropanoate (****7a****).* Yield: 60%; white solid; mp 48-50 ºC.

IR (KBr, ν cm^-1^): 3377, 3063, 3029, 2978, 1753, 1683, 1660, 1513, 1440, 1366, 1391, 1245, 1175; ^1^H NMR (500 MHz, MeOD): *δ* 7.25 (4H, t, *J* = 3.5 Hz), 7.19-7.15 (1H, m), 4.53 (1H, q, *J* = 9.0, 4.4 Hz), 4.26 (2H, d, *J* = 4.4 Hz), 4.04-4.02 (1H, m), 3.88 (1H, dd, *J* = 11.3, 4.8 Hz), 3.59 (2H, dd, *J* = 11.3, 4.1 Hz), 3.73 (3H, s), 2.90 (1H, dd, *J* = 13.6, 5.9 Hz), 2.75 (1H, dd, *J* = 13.6, 9.2 Hz), 1.35 (9H, s); ^13^C NMR (125.6 MHz, MeOD): *δ* 174.1 (C=O), 172.3 (C=O), 158.2 (C=O), 140.1 (C), 130.4 (CH Ar), 129.3 (CH Ar), 127.2 (CH Ar), 85.5 (CH), 80.1 (C), 70.4 (CH), 62.8 (CH), 60.7 (CH), 52.8 (OCH_3_), 41.5 (CH_2_), 38.7 (CH_2_), 28.7 (CH_3_); ESI-HRMS *m/z* [M + H]: Calcd for C_20_H_31_N_2_O_7_: 411.2053 (+H); found: 411.21239; [α]**_D_^25^** -32.2 (*c* 0.45; CH_2_Cl_2_).

*(S)-methyl-2-((3S,4S)-4-((tert-butoxycarbonyl)amino)-3-hydroxy-5-phenylpentanamido)-3-(4-hydroxyphenyl)propanoate (****7b****).* Yield: 65%; orange solid; mp 60-62 ºC. IR (KBr, ν cm^-1^): 3302, 2929, 1741, 1681, 1649, 1515, 1442, 1365, 1246, 1164; ^1^H NMR (500 MHz, MeOD): *δ* 7.25 (4H, d, *J* = 4.4 Hz), 7.19-7.14 (1H, m), 7.02 (2H, d, *J* = 8.4 Hz), 6.69 (2H, d, *J* = 8.5 Hz), 4.57 (1H, dd, *J* = 8.2, 5.8 Hz), 3.97-3.92 (1H, m), 3.66 (3H, s), 3.02 (1H, dd, *J* = 13.9, 5.7 Hz), 2.92-2.84 (2H, m), 2.78-2.70 (2H, m), 2.33 (2H, d, *J* = 6.6 Hz), 1.35 (9H, s); ^13^C NMR (125.6 MHz, MeOD): *δ* 173.9 (C=O), 173.7 (C=O), 158.4 (C=O), 157.4 (C), 140.1 (C), 131.2 (CH Ar), 130.2 (CH Ar), 129.3 (CH Ar), 128.6 (C), 127.2 (CH Ar), 116.2 (CH Ar), 80.2 (C), 70.5 (CH), 57.2 (CH), 55.7 (CH), 52.6 (OCH_3_), 41.6 (CH_2_), 38.8 (CH_2_), 37.6 (CH_2_), 28.7 (CH_3_); ESI-HRMS *m/z* [M + H]: Calcd for C_26_H_35_N_2_O_7_: 487.2366 (+H), found: 487.24375; [α]**_D_^25^** -27.3 (*c* 0.5; CH_2_Cl_2_).

*(2S,3S)-methyl-2-((3S,4S)-4-((tert-butoxycarbonyl)amino)-3-hydroxy-5-phenylpentanamido)-3-hydroxybutanoate (****7c****).* Yield: 55%; yellow solid; mp 124-125 ºC.

IR (KBr, ν cm^-1^): 3390, 3187, 3026, 2982, 1759, 1683, 1637, 1543, 1496, 1366, 1275;  ^1^H NMR (500 MHz, MeOD): *δ* 7.25 (4H, t, *J* = 3.0 Hz), 7.17-7.15 (1H, m), 4.46 (1H, d, *J* = 3.0 Hz), 4.27 (1H, dd, *J* = 6.4, 3.1 Hz), 4.05-4.02 (1H, m), 3.82-3.79 (1H, m), 3.73 (3H, s), 2.91 (1H, dd, *J* = 13.6, 5.8 Hz), 2.74 (1H, dd, *J* = 13.6, 9.1 Hz), 2.49-2.40 (2H, m), 1.35 (9H, s), 1.18 (3H, d, *J* = 6.4 Hz); ^13^C NMR (125.6 MHz, MeOD): *δ* 174.5 (C=O), 172.6 (C=O), 158.2 (C=O), 140.1 (C), 130.1 (CH Ar), 129.3 (CH Ar), 127.2 (CH Ar), 80.1 (C), 70.4 (CH), 68.4 (CH), 59.3 (CH), 57.6 (OCH_3_), 52.8 (CH_2_), 41.5 (CH_2_), 38.6 (CH), 28.7 (CH_3_), 20.3 (CH_3_); ESI-HRMS *m/z* [M + H]: Calcd for C_21_H_33_N_2_O_7_: 425.2210 (+H); Found: 425.22799; [α]**_D_^25^** -47.6 (*c* 0.45; CH_2_Cl_2_).

*(S)-methyl-3-((3S,4S)-4-((tert-butoxycarbonyl)amino)-3-hydroxy-5-phenylpentanamido)-2-hydroxypropanoate (****7d****).* Yield: 50%; white solid; mp 65-66 ºC.

IR (KBr, ν cm^-1^): 3367, 3063, 3029, 2981, 1736, 1679, 1519, 1443, 1366, 1391, 1272; ^1^H NMR (500 MHz, MeOD): *δ* 7.24 (4H, d, *J* = 4.3 Hz), 7.16 (1H, q, *J* = 8.6, 4.3 Hz), 4.56 (1H, br s), 4.26 (1H, q, *J* = 6.1, 4.8 Hz), 4.01-3.98 (1H, m), 3.75 (1H, d, *J* = 8.5 Hz), 3.72 (3H, s), 3.57 (1H, dd, *J* = 13.7, 4.6 Hz), 3.39 (1H, dd, *J* = 13.6, 6.4 Hz), 2.89 (1H, dd, *J* = 13.6, 6.0 Hz), 2.74 (1H, dd, *J* = 13.6, 9.1 Hz), 2.39-2.30 (2H, m), 1.34 (9H, s); ^13^C NMR (125.6 MHz, MeOD): *δ* 174.5 (C=O), 171.5 (C=O), 158.2 (C=O), 140.1 (C), 130.4 (CH Ar), 129.3 (CH Ar), 127.2 (CH Ar), 80.2 (C), 70.7 (CH), 70.4 (CH), 57.4 (CH), 52.7 (OCH_3_), 43.9 (CH_2_), 41.6 (CH_2_), 38.9 (CH_2_), 28.7 (CH_3_); ESI-HRMS *m/z* [M + H]: Calcd for C_20_H_31_N_2_O_7_: 411.2053 (+H); Found: 411.21243; [α]**_D_^25^** -38.8 (*c* 0.45; CH_2_Cl_2_).

*(S)-methyl-2-((3S,4S)-4-((tert-butoxycarbonyl)amino)-3-hydroxy-5-phenylpentanamido)-4-methylpentanoate (****7e****).* Yield: 55%; white solid; mp 129-130 ºC.

IR (KBr, ν cm^-1^): 3563, 3311, 2954, 1753, 1694, 1658, 1530, 1442, 1402, 1363, 1271; ^1^H NMR (500 MHz, CD_3_COCD_3_): *δ* 7.29-7.24 (4H, m), 7.17 (1H, t, *J* = 7.0 Hz), 5.69 (1H, d, *J* = 9.2 Hz), 3.97-3.94 (1H, m), 3.91-3.86 (1H, m), 3.97-3.94 (1H, m), 3.91-3.86 (1H, m), 3.65 (3H, s), 2.92 (1H, dd, *J* = 13.7, 6.6 Hz), 2.86 (1H, d, *J* = 8.5 Hz), 1.75-1.70 (1H, m), 1.58-1.55 (2H, m), 1.35 (9H, s), 0.90 (6H, dd, *J* = 10.1, 6.6 Hz); ^13^C NMR (125.6 MHz, CD_3_COCD_3_): *δ* 174.8 (C=O), 173.6 (C=O), 158.0 (C=O), 141.0 (C), 131.1 (CH Ar), 129.9 (CH Ar), 127.7 (CH Ar), 79.9 (C), 70.8 (CH), 57.4 (CH), 53.2 (CH), 52.5 (OCH_3_), 42.2 (CH_2_), 41.9 (CH_2_), 39.6 (CH_2_), 29.5 (CH_3_), 26.4 (CH), 24.1 (CH_3_), 22.8 (CH_3_); ESI-HRMS m/z [M + H]: Calcd for C_23_H_37_N_2_O_6_: 437.2573 (+H), found: 437.26445; [α]**_D_^25^** -34.6 (*c* 1.0; CH_2_Cl_2_).

*(S)-methyl-2-((3S,4S)-4-((tert-butoxycarbonyl)amino)-3-hydroxy-5-phenylpentanamido)-3-phenylpropanoate (****7f****).* Yield: 80%; white solid; mp 79-80 ºC (lit. 78-79 ºC). IR (KBr, ν cm^-1^): 3389, 3287, 3062, 2975, 1749, 1682, 1665, 1646, 1541, 1496, 1366, 1292, 1164; ^1^H NMR (500 MHz, CDCl_3_): *δ* 7.30-7.09 (10H, m), 6.50 (1H, br s), 4.90 (1H, br s), 4.80-4.79 (1H, m), 3.92 (1H, m), 3.77-3.72 (1H, m), 3.70 (3H, s), 3.16-3.12 (1H, m), 3.02 (1H, dd, *J* = 13.9, 6.6 Hz), 2.88 (2H, d, *J* = 7.4 Hz), 2.46-2.40 (1H, m), 2.19 (1H, d, *J* = 15 Hz), 1.40 (9H, s); ^13^C NMR (125.6 MHz, CDCl_3_): *δ* 172.2 (C=O), 171.7 (C=O), 156.2 (C=O), 138.1 (C), 135.7 (C), 129.3 (CH Ar), 129.1 (CH Ar), 128.5 (CH Ar), 128.4 (CH Ar), 127.1 (CH Ar), 126.3 (CH Ar), 79.4 (C), 68.0 (CH), 55.3 (CH), 53.2 (CH), 52.3 (OCH_3_), 40.0 (CH_2_), 38.4 (CH_2_), 37.6 (CH_2_), 28.3 (CH_3_), 27.9 (CH_3_); HRMS-FAB: *m/z* [M + 1] Calcd for C_26_H_34_N_2_O_6_: 471.2490 (+H); found: 471.2492; [α]_D_^20^ +25.2 (*c* 1.0; CH_2_Cl_2_).

*(S)-Methyl-1-((3’S,4’S)-4’-(tert-butoxycarbonylamino)-3’-hydroxy-5’-phenylpentanoyl) pyrrolidine-2-carboxylate (****7g****).* Yield: 70%; white solid; mp 118-119 ºC (lit. 119-120ºC). IR (KBr, ν cm^-1^): 3381, 3263, 2967, 2861, 1747, 1697, 1632, 1511, 1464, 1436, 1365, 1244, 1170. ^1^H NMR (500 MHz, CDCl_3_): *δ* 7.29-7.18 (5H, m), 4.45-4.42 (1H, m), 4.03 (1H, d, *J* = 9.6 Hz), 3.75-3.66 (1H, m), 3.71 (3H, s), 3.57-3.52 (1H, m), 3.46-3.42 (1H, m), 2.91 (2H, d, *J* = 7.7 Hz), 2.54-2.48 (1H, m), 2.35 (1H, d, *J* = 16.6 Hz), 2.21-2.13 (1H, m), 2.07-2.02 (1H, m), 2.00-1.96 (2H, m), 1.40 (9H, s); ^13^C NMR (125.6 MHz, CDCl_3_): *δ* 172.3 (C=O), 171.9 (C=O), 155.9 (C=O), 138.3 (C), 129.4 (CH Ar), 128.3 (CH Ar), 126.2 (CH Ar), 79.1 (C), 66.8 (CH), 58.5 (CH), 55.6 (CH), 52.2 (OCH_3_), 47.0 (CH_2_), 38.7 (CH_2_), 37.8 (CH_2_), 29.2 (CH_2_), 28.3 (CH_3_), 24.5 (CH_2_); HRMS-FAB: m/z [M + 1] Calcd for C_22_H_32_N_2_O_6_: 421.23331 (+H); found: 421.2331; [α]_D_^20^ -62.8 (*c* 1.0; CH_2_Cl_2_).

*(S)-Methyl-2-((3’S,4’S)-4’-(tert-butoxycarbonylamino)-3’-hydroxy-5’-phenylpentanamido)-3-methylbutanoate (****7h****).* Yield: 70%; yellowish solid; mp 47-48 ºC.

IR (KBr, ν cm^-1^): 3288, 3062, 2966, 1743, 1685, 1648, 1536, 1453, 1391, 1365, 1250; ^1^H NMR (500 MHz, CDCl_3_): *δ* 7.29-7.18 (5H, m), 6.58 (1H, br s), 4.92 (1H, br s), 4.50-4.47 (1H, m), 3.99-3.96 (1H, d, *J* = 7.6 Hz), 3.81-3.73 (1H, m), 3.72 (3H, s), 2.90 (2H, d, *J* = 7.4 Hz), 2.58-2.53 (1H, m), 2.28 (1H, d, *J* = 13.3 Hz), 2.14 (1H, dd, *J* = 12.0, 6.8 Hz), 1.40 (9H, s), 0.93 (3H, d, *J* = 7.0 Hz), 0.91 (3H, d, *J* = 7.0 Hz); ^13^C NMR (125.6 MHz, CDCl_3_): *δ* 172.5 (C=O), 172.1 (C=O), 156.2 (C=O), 138.1 (C), 129.3 (CH Ar), 128.3 (CH Ar), 126.3 (CH Ar), 79.4 (C), 68.1 (CH), 57.1 (CH), 55.4 (CH), 52.1 (OCH_3_), 40.0 (CH_2_), 38.5 (CH_2_), 30.9 (CH), 28.3 (CH_3_), 18.9 (CH_3_), 17.8 (CH_3_); HRMS-FAB: m/z [M + 1] Calcd for C_22_H_34_N_2_O_6_: 423.24896 (+H); found: 423.2485; [α]_D_^20^ -13.0 (*c* 1.0; CH_2_Cl_2_).

# **Characterization and yields of the synthetic peptidomimetic compounds 8a-g**

*(S)-methyl-3-acetoxy-2-((3S,4S)-3-acetoxy-4-((tert-butoxycarbonyl)amino)-5-phenylpentanamido)propanoate (****8a****).* Yield: 85 %; white solid; mp 97-98 ºC. IR (KBr, ν cm^-1^): 3342, 2980, 1739, 1687, 1652, 1437, 1367, 1316, 1166. ^1^H NMR (500 MHz, CDCl_3_): *δ* 7.26-7.24 (2H, m), 7.21-7.17 (3H, m), 5.29-5.24 (1H, m), 4.80 (1H, q, *J* = 7.6, 3.6 Hz), 4.68 (1H, d *J* = 9.8 Hz), 4.44 (1H, dd, *J* = 11.4, 3.8 Hz), 4.32 (1H, dd, *J* = 11.4, 3.5 Hz), 3.75 (3H, s), 2.84 (1H, dd, *J* = 14.1, 5.9 Hz), 2.66 (1H, dd, *J* = 14.1, 8.9 Hz), 2.54 (2H, d, *J* = 6.7 Hz), 2.08 (3H, s), 2.03 (3H, s), 1.35 (9H, s). ^13^C NMR (125.6 MHz, CDCl_3_): *δ* 170.4 (C=O), 169.7 (C=O), 169.05 (C=O), 169.0 (C=O), 156.1 (C=O), 137.1 (C), 129.0 (CH Ar), 128.4 (CH Ar), 126.5 (CH Ar), 79.8 (C), 72.0 (Ch), 63.6 (CH_2_), 53.4 (CH), 52.7 (CH), 51.7 (OCH_3_), 39.2 (CH_2_), 38.9 (CH_2_), 28.2 (CH_3_), 20.9 (CH_3_), 20.6 (CH_3_). ESI-HRMS *m/z* [M + H]: Calcd for C_24_H_35_N_2_O_9_: 495.2264 (+H), Found: 495.23362. [α]**_D_^25^** -46.3 (*c* 0.50; CH_2_Cl_2_).

*(S)-methyl-2-((3S,4S)-3-acetoxy-4-((tert-butoxycarbonyl)amino)-5-phenylpentanamido)-3-(4-acetoxyphenyl)propanoate (****8b****).* Yield: 65 %; yellow solid; mp 88-89 ºC. IR (KBr, ν cm^-1^): 3350, 2931, 1739, 1687, 1651, 1435, 1367, 1308, 1194; ^1^H NMR (500 MHz, CDCl_3_): *δ* 7.27 (3H, t, *J* = 7.5 Hz), 7.21-7.16 (4H, m), 7.09 (1H, d, *J* = 6.3 Hz), 6.98 (2H, d, *J* = 8.3 Hz), 4.73 (1H, q, *J* = 13.0, 7.5 Hz), 4.66 (1H, d, *J* = 4.6 Hz), 4.33 (1H, q, *J* = 15.2, 8.5 Hz), 3.70 (3H, s), 3.14 (1H, dd, *J* = 14.0, 5.1 Hz), 3.00 (1H, dd, *J* = 14.0, 8.1 Hz), 2.84 (1H, dd, *J* = 14.1, 5.8 Hz), 2.65 (1H, dd, *J* = 14.0, 8.9 Hz), 2.49-2.41 (2H, m), 2.27 (3H, s), 2.05 (3H, s), 1.36 (9H, s). ^13^C NMR (125.6 MHz, CDCl_3_): *δ* 172.1 (C=O), 169.9 (C=O), 169.5 (C=O), 169.1 (C=O), 156.4 (C=O), 149.8 (C), 137.3 (C), 134.1 (CH), 130.3 (CH Ar), 129.3 (CH Ar), 128.6 (CH Ar), 126.7 (CH Ar), 121.7 (CH Ar), 80.1 (C), 72.4 (CH), 54.0 (CH), 53.4 (CH), 52.5 (OCH_3_), 39.3 (CH_2_), 39.2 (CH_2_), 37.0 (CH_2_), 29.8 (CH_3_), 21.3 (CH_3_), 21.1 (CH_3_); ESI-HRMS *m/z* [M + H]: Calcd for C_30_H_39_N_2_O_9_: 571.2577 (+H), Found: 571.26508. [α]**_D_^25^** -23,312 (*c* 0.50; CH_2_Cl_2_).

*(2S,3S)-methyl-3-acetoxy-2-((3S,4S)-3-acetoxy-4-((tert-butoxycarbonyl)amino)-5-phenylpentanamido)butanoate (****8c****).* Yield: 75 %; white solid; mp 54-55 ºC. IR (KBr, ν cm^-1^): 3289, 2933, 1732, 1693, 1675, 1495, 1454, 1364, 1196; ^1^H NMR (500 MHz, CDCl_3_): *δ* 7.26 (2H, t, *J* = 5.5), 7.19 (3H, d, *J* = 7.0 Hz), 5.45-5.38 (1H, m), 5.27 (1H, t, *J* = 6.5 Hz), 4.70 (1H, dd, *J* = 9.0, 2.0 Hz), 4.36-4.29 (1H, m), 3.71 (3H, s), 2.85 (1H, dd, *J* = 14.1, 6.0 Hz), 2.68 (1H, dd, *J* = 14.0, 8.5 Hz), 2.58 (2H, q, *J* = 14.0, 6.5 Hz), 2.08 (3H, s), 2.00 (3H, s), 1.36 (9H, s), 1.22 (3H, d, *J* = 6.4 Hz). ^13^C NMR (125.6 MHz, CDCl_3_): *δ* 170.1 (C=O), 169.7 (C=O), 169.5 (C=O), 156.1 (C=O), 137.1 (C), 129.0 (CH Ar), 128.4 (CH Ar), 126.5 (CH Ar), 79.8 (C), 72.1 (CH), 69.9 (CH), 55.7 (CH), 53.4 (CH), 52.6 (OCH_3_), 39.3 (CH_2_), 38.8 (CH_2_), 28.1 (CH_3_), 20.9 (CH_3_), 20.8 (CH_3_) 17.0 (CH_3_); ESI-HRMS *m/z* [M + H]: Calcd for C_25_H_37_N_2_O_9_: 509.2421 (+H), Found: 509.24917; [α]**_D_^25^** -38.9 (*c* 0.40; CH_2_Cl_2_).

*(S)-methyl-2-((3S,4S)-3-acetoxy-4-((tert-butoxycarbonyl)amino)-5-phenylpentanamido)-4-methylpentanoate (****8d****).* Yield: 90 %; white solid; mp 94-95 ºC. IR (KBr, ν cm^-1^): 3308, 2954, 1740, 1668, 1529, 1436, 1364, 1236, 1166; ^1^H NMR (500 MHz, CDCl_3_): *δ* 7.26 (2H, t, *J* = 2.5 Hz), 7.21-7.19 (3H, m), 5.24 (1H, t, *J* = 6.5 Hz), 4.49 (1H, q, *J* = 14.0, 7.5 Hz), 4.38 (1H, q, *J* = 15.0, 8.5 Hz), 3.70 (3H, s), 2.85 (1H, dd, *J* = 14.1, 5.7 Hz), 2.66 (1H, dd, *J* = 14.1, 8.8 Hz), 2.57-2.44 (2H, m), 2.08 (3H, s), 1.64 (2H, s), 1.30 (1H, d *J* = 5.7 Hz), 1.36 (9H, s), 0.92 (6H, t, *J* = 5.0 Hz); ^13^C NMR (125.6 MHz, CDCl_3_): *δ* 174.3 (C=O), 172.6 (C=O), 170.8 (C=O), 155.0 (C=O), 141.0 (C), 131.1 (CH Ar), 129.9 (CH Ar), 127.7 (CH Ar), 79.5 (C), 70.8 (CH), 57.4 (CH), 53.2 (CH), 52.5 (OCH_3_), 42.1 (CH_2_), 41.9 (CH_2_), 39.8 (CH_2_), 29.5 (CH_3_), 26.4 (CH), 24.2 (CH_3_), 22.6 (CH_3_); ESI-HRMS *m/z* [M + H]: Calcd for C_25_H_39_N_2_O_7_: 479.2679 (+H), Found: 479.27497; [α]**_D_^25^** +55.6 (*c* 0.35; CH_2_Cl_2_).

*(S)-methyl-2-((3S,4S)-3-acetoxy-4-((tert-butoxycarbonyl)amino)-5-phenylpentanamido)-3-phenylpropanoate (****8e****).* Yield: 80 %; yellow solid; mp 89-91 ºC. IR (KBr, ν cm^-1^): 3063, 3030, 2931, 1740, 1660, 1524, 1454, 1438, 1366, 1164; ^1^H NMR (500 MHz, CDCl_3_): *δ* 7.30-7.14 (10H, m), 7.01 (1H, br s), 5.22 (1H, t, *J* = 5.9 Hz), 4.76 (1H, q, *J* = 12.9, 7.6 Hz), 4.66 (1H, d, *J* = 10.1 Hz), 3.70 (3H, s), 3.15 (1H, dd, *J* = 13.9, 5.3 Hz), 3.01 (1H, dd, *J* = 13.9, 7.8 Hz), 2.84 (1H, dd, *J* = 14.1, 5.9 Hz), 2.66 (1H, dd, *J* = 14.1, 8.7 Hz), 2.46-2.43 (2H, m), 2.04 (3H, s), 1.36 (6H, s), 1.25 (3H, s); ^13^C NMR (125.6 MHz, CDCl_3_): *δ* 172.3 (C=O), 169.9 (C=O), 169.0 (C=O), 156.4 (C=O), 137.3 (C), 136.5 (C), 129.4 (CH Ar), 129.3 (CH Ar), 128.6 (CH Ar), 127.1 (CH Ar), 126.7 (CH Ar), 80.1 (C), 72.3 (CH_2_), 54.0 (CH), 53.4 (CH), 52.4 (OCH_3_), 39.3 (CH_2_), 39.2 (CH_2_), 37.7 (CH_2_), 28.4 (CH_3_), 21.1 (CH_3_); ESI-HRMS *m/z* [M + H]: Calcd for C_28_H_37_N_2_O_7_: 513.2523 (+H); Found: 513.25936; [α]**_D_^25^** -42.5 (*c* 0.50; CH_2_Cl_2_).

*(S)-methyl-1-((3S,4S)-3-acetoxy-4-((tert-butoxycarbonyl)amino)-5-phenylpentanoyl)pyrrolidine-2-carboxylate (****8f****).* Yield: 60 %; white solid; mp 90-92 ºC. IR (KBr, ν cm^-1^): 3256, 2973, 1735, 1704, 1629, 1533, 1385, 1241, 1166; ^1^H NMR (500 MHz, CDCl_3_): *δ* 7.27-7.24 (2H, m), 7.20-7.18 (3H, m), 4.75 (1H, d, *J* = 9,6 Hz), 4.46-4.44 (1H, m), 4.13-4.08 (1H, m), 3.70 (3H, s), 3.57 (2H, t, *J* = 6.0 Hz), 3.48- 3.43 (2H, m), 2.87-2.82 (2H, m), 2.64 (2H, t, *J* = 5.0 Hz), 2.04 (3H, t, *J* = 6.7 Hz), 1.98-1.95 (2H, m), 1.34 (9H, d *J* = 7.0 Hz); ^13^C NMR (125.6 MHz, CDCl_3_): *δ* 172.6 (C=O), 170.0 (C=O), 168.2 (C=O), 155.6 (C=O), 137.6 (C), 129.4 (CH Ar), 128.5 (CH Ar), 126.6 (CH Ar), 79.5 (C), 72.3 (CH), 58.8 (CH), 54.4 (CH), 52.3 (OCH_3_), 47.2 (CH_2_), 39.0 (CH_2_), 37.0 (CH_2_), 29.3 (CH_2_), 28.4 (CH_3_), 24.9 (CH_2_), 21.2 (CH_3_); ESI-HRMS *m/z* [M + H]: Calcd for C_24_H_35_N_2_O_7_: 463.2366 (+H); Found: 463.24362; [α]**_D_^25^** -86.6 (*c* 0.25; CH_2_Cl_2_).

*(S)-methyl-2-((3S,4S)-3-acetoxy-4-((tert-butoxycarbonyl)amino)-5-phenylpentanamido)-3-methylbutanoate (****8g****).* Yield: 70 %; white solid; mp 78-80 ºC. IR (KBr, ν cm^-1^): 3310, 2966, 2931, 1736, 1663, 1534, 1454, 1366, 1306, 1167; ^1^H NMR (500 MHz, CDCl_3_): *δ* 7.28-7.25 (2H, m), 7.20-7.18 (3H, m), 7.07 (1H, br s), 5.24 (1H, t, *J* = 6.0 Hz), 4.68 (1H, d, *J* = 9.8 Hz), 4.46 (1H, q, *J* = 8.3, 5.0 Hz), 4.30 (1H, q, *J* = 15.5, 7.5 Hz), 3.71 (3H, s), 2.84 (1H, dd, *J* = 14.1, 6.0 Hz), 2.68 (1H, dd, *J* = 14.1, 8.6 Hz), 2.55 (1H, dd, *J* = 14.0, 7.5 Hz), 2.48 (1H, dd, *J* = 13.7, 5.7 Hz), 2.20-2.14 (1H, m), 2.08 (3H, s), 1.36 (6H, s), 1.25 (3H, s), 0.91 (6H, d, *J* = 6.7 Hz); ^13^C NMR (125.6 MHz, CDCl_3_): *δ* 172.5 (C=O), 169.9 (C=O), 169.2 (C=O), 156.3 (C=O), 137.3 (C), 129.2 (CH Ar), 128.6 (CH Ar), 126.7 (CH Ar), 80.1 (C), 72.4 (CH), 57.7 (CH), 53.6 (CH), 52.2 (OCH_3_), 39.6 (CH_2_), 39.1 (CH_2_), 30.7 (CH), 28.4 (CH_3_), 21.1 (CH_3_), 17.9 (CH_3_); ESI-HRMS *m/z* [M + H]: Calcd for C_24_H_37_N_2_O_7_: 465.2523 (+H); Found: 465.25944; [α]**_D_^25^** -27.372 (*c* 0.50; CH_2_Cl_2_).

# **Characterization and yields of the synthetic peptidomimetic compounds 9a-g**

*(2S,3S)-3-hydroxy-5-(((S)-3-hydroxy-1-methoxy-1-oxopropan-2-yl)amino)-5-oxo-1-phenylpentan-2-aminium 2,2,2-trifluoroacetate (****9a****).* Yield: 95%; yellow solid; mp 120-121 ºC. IR (KBr, ν cm^-1^): 3265, 3066, 2955, 1738, 1668, 1438, 1289, 1181; ^1^H NMR (500 MHz, MeOD): *δ* 7.36 (2H, t, *J* = 7.4 Hz), 7.31-7.27 (3H, m), 4.53 (1H, t, *J* = 4.3 Hz), 4.26 (2H, d, *J* = 4.4 Hz) 4.06-4.03 (1H, m), 3.90 (1H, dd, *J* = 11.2, 4.8 Hz), 3.78 (1H, dd, *J* = 11.2, 3.9 Hz), 3.74 (3H, s), 3.57-3.53 (1H, m), 3.09 (1H, dd, *J* = 14.0, 7.4 Hz), 2.90 (1H, dd, *J* = 14.0, 7.4 Hz); ^13^C NMR (125.6 MHz, MeOD): *δ* 172.9 (C=O), 172.3 (C=O), 162.6 (C=O), 137.0 (C), 130.4 (CH Ar), 130.0 (CH Ar), 128.4 (CH Ar), 67.3 (CH), 62.3 (CH_2_), 57.6 (CH), 56.2 (CH), 52.9 (OCH_3_), 41.4 (CH_2_), 37.3 (CH_2_); ESI-HRMS *m/z* [M + H]: Calcd for C_16_H_24_N_2_O_5_: 311.1529 (+H); Found: 311.15987; [α]**_D_^25^** +5.0 (*c* 0.50; CH_3_OH).

*(2S,3S)-3-hydroxy-5-(((S)-3-(4-hydroxyphenyl)-1-methoxy-1-oxopropan-2-yl)amino)-5-oxo-1-phenylpentan-2-aminium 2,2,2-trifluoroacetate (****9b****).* Yield: 90%; yellow solid; mp 98-99 ºC. IR (KBr, ν cm^-1^): 3259, 1732, 1670, 1515, 1439, 1373, 1182; ^1^H NMR (500 MHz, MeOD): *δ* 7.35 (2H, t, *J* = 7.3 Hz), 7.30-7.26 (3H, m), 6.98 (2H, d, *J* = 8.5 Hz), 6.69 (2H, d, *J* = 8.5 Hz), 4.60 (1H, dd, *J* = 8.2, 5.8 Hz), 3.98-3.95 (1H, m), 3.67 (3H, s), 3.49-3.45 (1H, m), 3.06-2.99 (2H, m), 2.89-2.85 (2H, m), 2.55 (1H, dd, *J* = 15, 5.75 Hz), 2.47 (1H, dd, *J* = 15, 6.5 Hz); ^13^C NMR (125.6 MHz, MeOD): *δ* 173.6 (C=O), 172.6 (C=O), 157.4 (C), 136.9 (C), 131.2 (CH Ar), 130.4 (CH Ar), 130.0 (CH Ar), 128.5 (CH Ar), 128.4 (C), 116.3 (CH Ar), 67.3 (CH), 57.6 (CH), 55.5 (CH), 52.7 (OCH_3_), 41.2 (CH_2_), 37.5 (CH_2_), 37.3 (CH_2_); ESI-HRMS *m/z* [M + H]: Calcd for C_21_H_27_N_2_O_5_: 387.1842 (+H); Found: 387.19118; [α]**_D_^25^** +6.3 (*c* 0.50; CH_3_OH).

*(2S,3S)-3-hydroxy-5-(((2S,3R)-3-hydroxy-1-methoxy-1-oxobutan-2-yl)amino)-5-oxo-1-phenylpentan-2-aminium 2,2,2-trifluoroacetate (****9c****).* IR (KBr, ν cm^-1^): 3276, 3066, 2900, 1737, 1669, 1530; 1437, 1289, 1200; 1182; ^1^H NMR (500 MHz, MeOD): *δ* 7.36-7.29 (5H, m), 4.81 (1H, br s), 4.45 (1H, d, *J* = 3.0 Hz), 4.30 (1H, dd, *J* = 6.4, 3.0 Hz), 4.08-4.03 (1H, m), 3.74 (3H, s), 3.59-3.53 (1H, m), 3.10 (1H, dd, *J* = 14.0, 7.4 Hz), 2.90 (1H, dd, *J* = 14.0, 7.4 Hz), 2.64-2.61 (2H, m), 1.16 (3H, d, *J* = 6.4 Hz); ^13^C NMR (125.6 MHz, MeOD): *δ* 172.9 (C=O), 172.5 (C=O), 166.2 (C=O), 136.6 (C), 130.2 (CH Ar), 129.9 (CH Ar), 128.3 (CH Ar), 67.9 (CH), 67.1 (CH), 59.1 (CH), 57.4 (CH), 52.9 (OCH_3_), 41.2 (CH_2_), 37.1 (CH_2_), 20.3 (CH_3_); ESI-HRMS *m/z* [M + H]: Calcd for C_16_H_25_N_2_O_5_: 325.1758 (+H); Found: 325.17542.

*(2S,3S)-3-hydroxy-5-(((S)-1-methoxy-4-methyl-1-oxopentan-2-yl)amino)-5-oxo-1-phenylpentan-2-aminium 2,2,2-trifluoroacetate (****9d****).* Yield: 90%; white solid; mp 147-148 ºC. IR (KBr, ν cm^-1^): 3341, 3210, 2953, 2929, 1727, 1659, 1496, 1474, 1455, 1269, 1182; ^1^H NMR (500 MHz, MeOD): *δ* 7.35 (2H, t, *J* = 6.7 Hz), 7.20 (3H, d, *J* = 7.4 Hz), 4.43 (1H, t, *J* = 7.5 Hz), 4.03-4.00 (1H, m), 3.69 (3H, s), 3.51 (1H, sext, *J* = 7.4, 3.7 Hz), 3.08 (1H, dd, *J* = 14.0, 7.6 Hz), 2.90 (1H, dd, *J* = 14.0, 7.3 Hz) 2.58-2.53 (2H, oct, *J* = 14.8, 5.9 Hz), 1.64 (1H, q, *J* = 13.2, 6.5 Hz), 1.60-1.56 (2H, m) 0.92 (6H, dd, *J* = 19.1, 6.2 Hz); ^13^C NMR (125.6 MHz, MeOD): *δ* 174.4 (C=O), 172.9 (C=O), 138.6 (C), 129.3 (CH Ar), 128.3 (CH Ar), 126.3 (CH Ar), 79.4 (CH), 59.1 (CH), 57.1 (CH), 51.9 (OCH_3_), 40.6 (CH_2_), 40.0 (CH_2_), 30.9 (CH_2_), 24.8 (CH), 28.3 (CH_3_); ESI-HRMS *m/z* [M + H]: Calcd for C_18_H_29_N_2_O_4_: 337.2122 (+H); Found: 337.21179; [α]**_D_^25^** +3.0 (*c* 0.30; CH_3_OH).

*(2S,3S)-3-hydroxy-5-(((S)-1-methoxy-1-oxo-3-phenylpropan-2-yl)amino)-5-oxo-1-phenylpentan-2-aminium 2,2,2-trifluoroacetate (****9e****).* Yield: 90%; white solid; mp 140-142 ºC. IR (KBr, ν cm^-1^): 3325, 3208, 3065, 2945, 1729, 1655, 1445, 1436, 1393, 1317, 1267, 1206, 1178; ^1^H NMR (500 MHz, DMSO-*d*_6_): *δ* 8.39 (1H, d, *J* = 8,6 Hz), 7.83 (3H, br s), 7.33 (2H, t, *J* = 15, 7.5 Hz), 7.27-7.24 (5H, m), 7.21-7.17 (3H, m), 5.72 (1H, br s), 4.48-4.43 (1H, m), 3.84-3.80 (1H, m), 3.57 (3H, s), 2.99 (1H, dd, *J* = 13.9, 5.8 Hz), 2.91-2.86 (2H, m), 2.82 (2H, dd, *J* = 13.9, 6.1 Hz), 2.40-2.38 (2H, m); ^3^C NMR (125.6 MHz, DMSO-*d*_6_): *δ* 171.5 (C=O), 169.7 (C=O), 157.6 (C=O), 136.7 (C), 136.2 (C), 129.0 (CH Ar), 128.7 (CH Ar), 128.2 (CH Ar), 127.9 (CH Ar), 126.5 (CH Ar), 126.3 (CH Ar), 65.0 (CH), 55.2 (CH), 51.5 (CH), 51.5 (OCH_3_), 36.4 (CH_2_), 35.3 (CH_2_), 34.4 (CH_2_); ESI-HRMS *m/z* [M + H]: Calcd for C_21_H_27_N_2_O_4_: 371.1893 (+H); Found: 371.19616; [α]**_D_^25^** +4.5 (*c* 0.5; CH_3_OH).

*(2S,3S)-3-hydroxy-5-((S)-2-(methoxycarbonyl)pyrrolidin-1-yl)-5-oxo-1-phenylpentan-2-aminium 2,2,2-trifluoroacetate (****9f****).* Yield: 95%; white solid; mp 138-140 ºC. IR (KBr, ν cm^-1^): 3382, 2651, 1750, 1687, 1454, 1380, 1327, 1203, 1176; ^1^H NMR (500 MHz, DMSO-*d*_6_): *δ* 7.84 (3H, br s), 7.34 (2H, t, *J* = 14.7, 7.3 Hz), 7.28-7.23 (3H, m), 4.26 (1H, q, *J* = 8.7, 4.3 Hz), 3.95-3.93 (1H, m), 3.67 (1H, br s), 3.59 (3H, s), 3.49 (2H, t, *J* = 13.5, 6.7 Hz), 2.92 (1H, dd, *J* = 13.7, 8.6 Hz), 2.86 (2H, dd, 13.7, 6.0 Hz), 2.66 (1H, dd, *J* = 15.9, 8.1 Hz), 2.17-2.10 (1H, m), 1.89 (2H, q, *J* = 13.8, 6.8 Hz), 1.85-1.78 (2H, m); ^13^C NMR (125.6 MHz, DMSO-*d*_6_): *δ* 172.2 (C=O), 168.7 (C=O), 157.8 (C=O), 136.3 (C), 129.2 (CH Ar), 128.3 (CH Ar), 126.6 (CH Ar), 64.5 (CH), 58.0 (CH), 55.4 (CH), 51.5 (OCH_3_), 46.6. (CH_2_), 38.2 (CH_2_), 35.5 (CH_2_), 28.6 (CH_2_), 24.1 (CH_2_); ESI-HRMS *m/z* [M + H]: Calcd for C_17_H_25_N_2_O_4_: 321.1736 (+H); Found: 321.18059; [α]**_D_^25^** -20.1 (*c* 0.47; CH_3_OH).

*(2S,3S)-3-hydroxy-5-(((S)-1-methoxy-3-methyl-1-oxobutan-2-yl)amino)-5-oxo-1-phenylpentan-2-aminium 2,2,2-trifluoroacetate (****9g****).* Yield: 90%; yellow solid; mp 105-106 ºC. IR (KBr, ν cm^-1^): 3434, 3342, 3030, 2921, 1746, 1672, 1429, 1376, 1262, 1183; ^1^H NMR (500 MHz, DMSO-*d*_6_): *δ* 7.37-7.35 (2H, m), 7.30-7.28 (3H, m), 4.32 (1H, d, *J* = 6.0 Hz) 4.04-4.00 (1H, m), 3.92-3.88 (1H, m), 3.70 (3H, s), 3.53-3.49 (1H, m), 3.09-3.05 (1H, m), 2.90 (1H, dd, *J* = 14.0, 7.2 Hz), 2.80 (1H, dd, *J* = 13.6, 7.4 Hz), 2.59 (1H, oct, *J* = 15.0, 5.8 Hz), 0.91 (6H, d, *J* = 6.8 Hz); ^13^C NMR (125.6 MHz, DMSO-*d*_6_): *δ* 160.1 (C=O), 157.7 (C=O), 138.5 (C=O), 136.2 (C), 129.0 (CH Ar), 128.2 (CH Ar), 126.5 (CH Ar), 115.8 (CF_3_), 65.1 (CH), 57.2 (CH), 55.2 (CH), 51.3 (OCH_3_), 40.5 (CH_2_), 35.2 (CH_2_), 30.2 (CH), 29.5 (CH_3_); ESI-HRMS *m/z* [M + H]: Calcd for C_17_H_27_N_2_O_4_: 323.1893 (+H); Found: 323.19619; [α]**_D_^25^** -15.6 (*c* 0.43; CH_3_OH).

*tert-butyl-((2S,3S)-5-(((S)-1-hydrazinyl-1-oxo-3-phenylpropan-2-yl)amino)-3-hydroxy-5-oxo-1-phenylpentan-2-yl)carbamate (****10****).* The corresponding ester (**7f**) (0.2 mg; 0.228 mmol) was solubilized in methanol (5 mL). To this solution was added hydrazine monohydrate (0.5 mL; 0.345 mmol) and the resulting suspension was refluxed for 1 h. The mixture was evaporated in a rotary evaporator and diethyl ether was added and evaporated 3 times. The resulting product was purified by recrystallization from diethyl ether. Yield: 96 %; white solid; mp 192-193 ºC. IR (KBr, ν cm^-1^): 3308, 2967, 2935, 1680, 1641, 1597, 1529, 1444, 1330, 1273, 1247, 1170; ^1^H NMR (500 MHz, DMSO-*d*_6_): *δ* 8.98 (1H, br s), 7.87 (1H, br s), 7.26-7.15 (10H, m), 6.24 (1H, br s), 4.48-4.44 (1H, m), 3.82 (1H, br s), 3.66 (1H, br s), 2.96 (1H, dd, *J* = 13.7, 5.3 Hz), 2.82-2.77 (2H, m), 2.65-2.60 (1H, m), 2.22-2.18 (2H, m), 1.31 (9H, s); ^13^C NMR (125.6 MHz, DMSO-*d*_6_): *δ* 170.4 (C=O), 169.9 (C=O), 155.2 (C=O), 139.0 (C), 137.5 (C), 128.7 (CH Ar), 127.7 (CH Ar), 127.6 (CH Ar), 125.9 (CH Ar), 125.4 (CH Ar), 77.5 (C), 68.3 (CH), 55.5 (CH), 52.5 (CH), 37.5 (CH_2_), 36.4 (CH_2_), 33.0 (CH_2_), 27.9 (CH_3_); ESI-HRMS *m/z* [M + H]: Calcd for C_25_H_35_N_4_O_5_: 471.2529 (+H), Found: 471.25976.


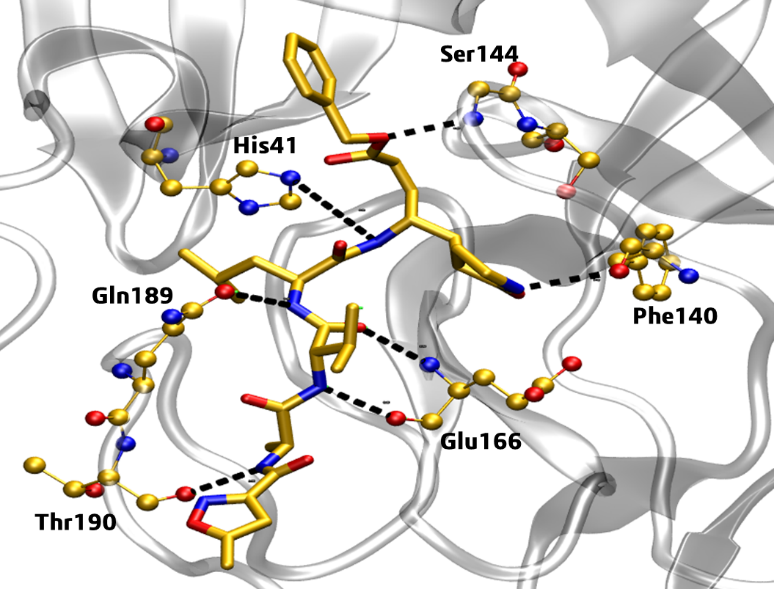


# **Figure S1.** N3 hydrogen bonds in the active site of Mpro.


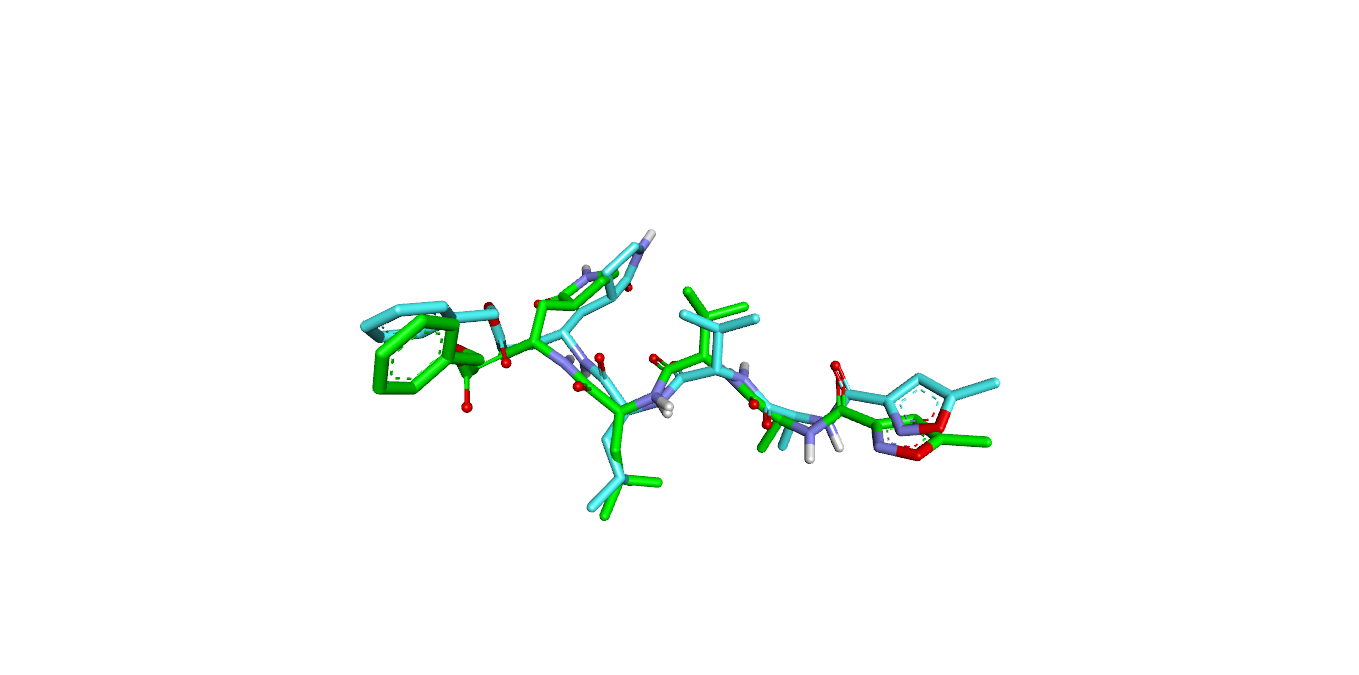


# **Figure S2.** Best pose of redocking result for ligand N3 (in blue) compared to original one (in green).

# **Spectra of compound 7a**.

IR (KBr)


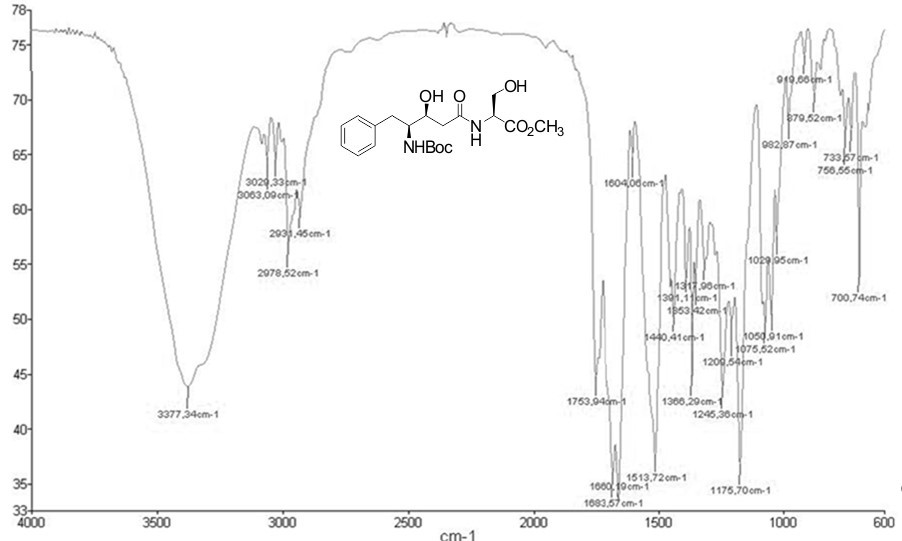


(7

a

)

^1^H NMR (500 MHz, MeOD)


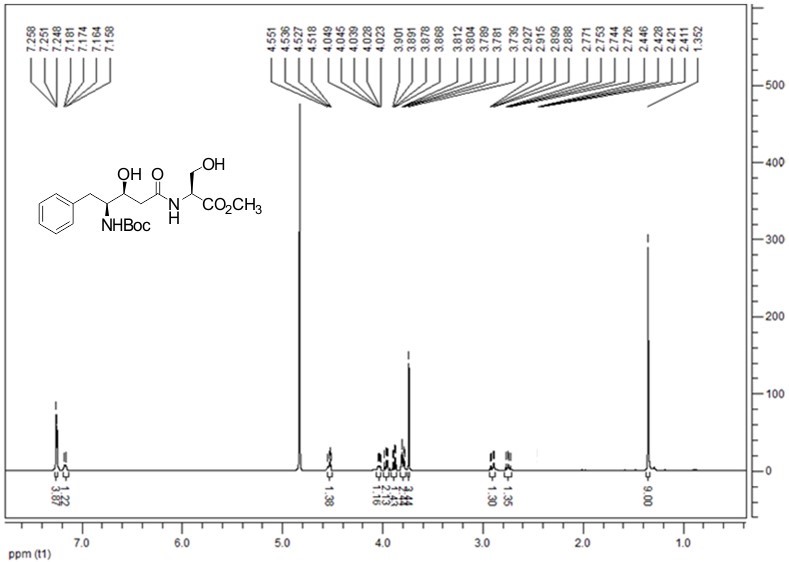


(7

a

)

APT (125.6 MHz, MeOD)


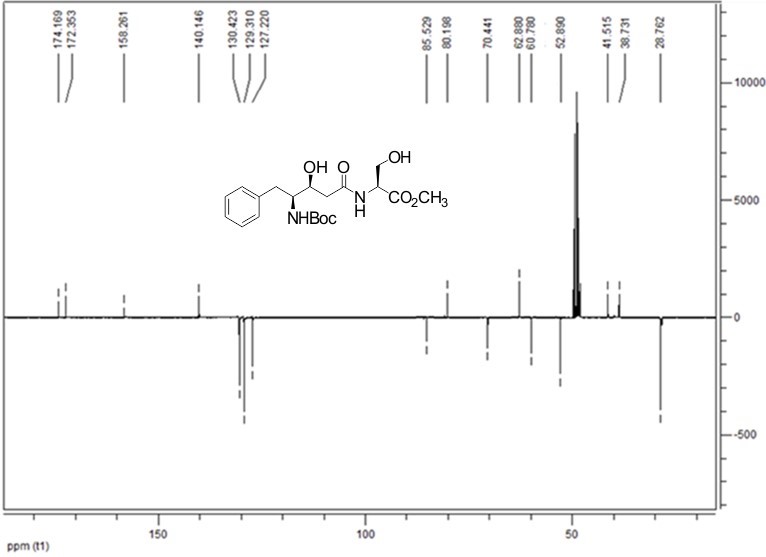


(7

a

)

ESI-HRMS


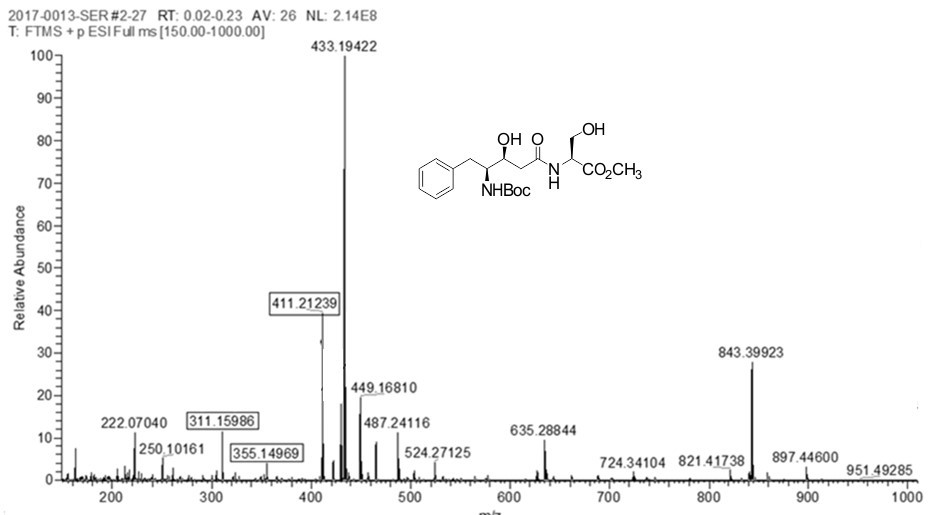


(7

a

)

# **Spectra of compound 7b**.

IR (KBr)


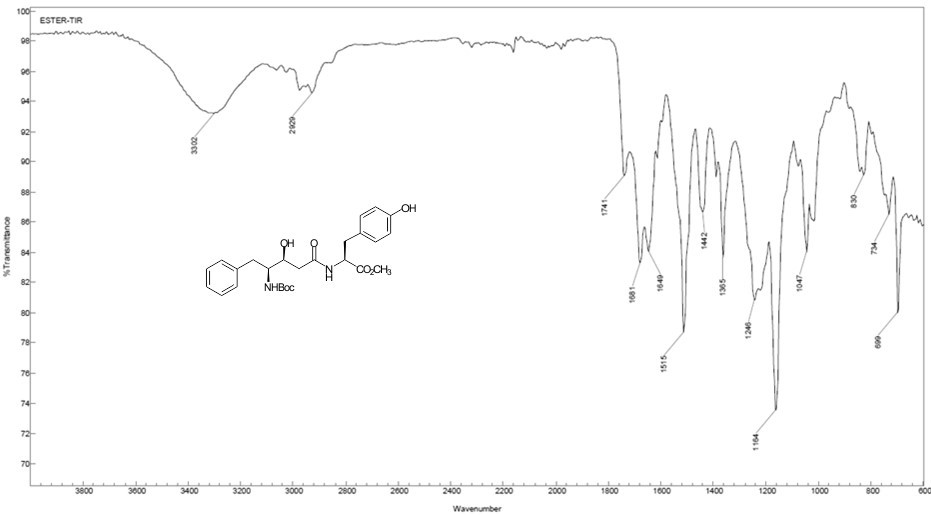


(7

b

)

^1^H NMR (500 MHz, MeOD)


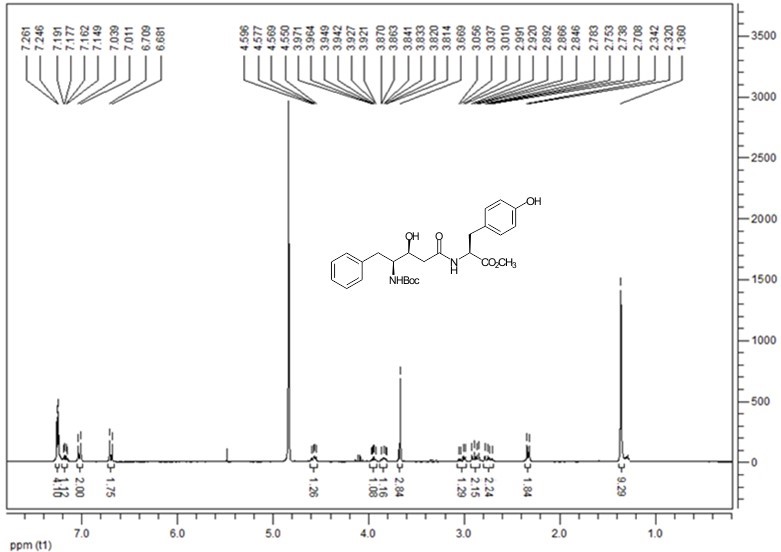


(7

b

)

APT (125.6 MHz, MeOD)


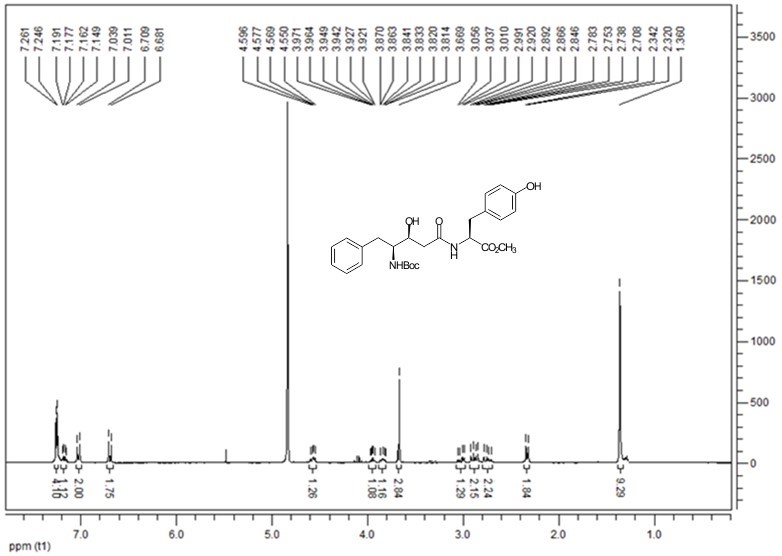


(7

b

)

HRMS-FAB


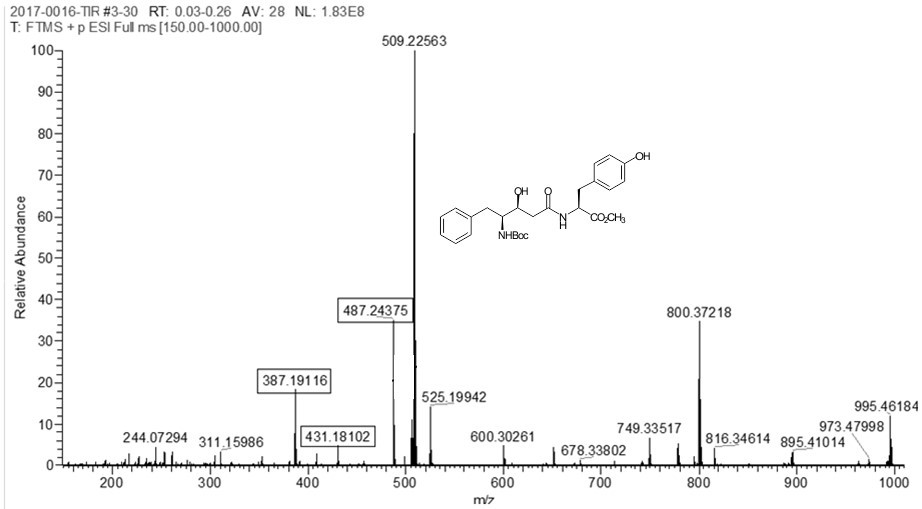


(7

b

)

# **Spectra of compound 7c**.

IR (KBr)


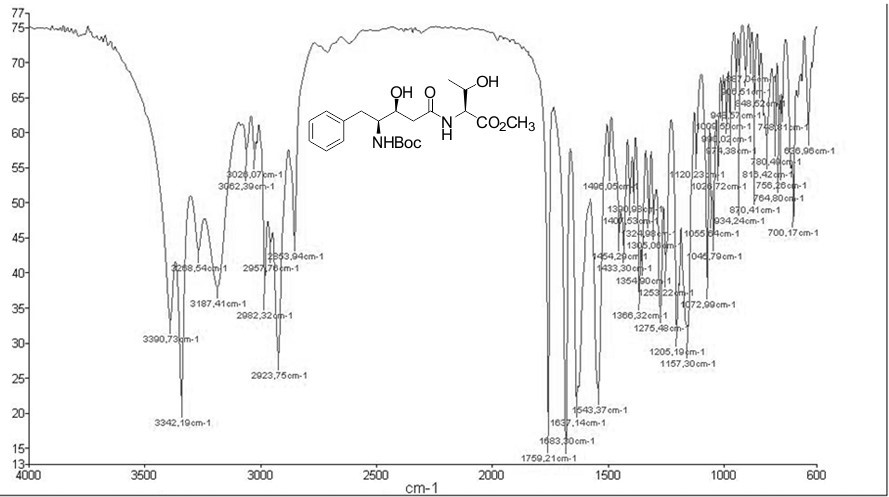


(7

c

)

^1^H NMR (500 MHz, MeOD)


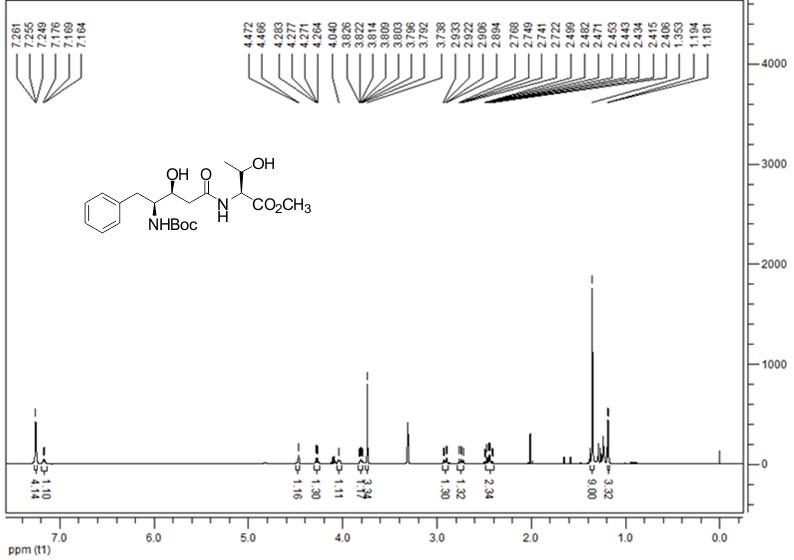


(7

c

)

APT (125.6 MHz, MeOD)


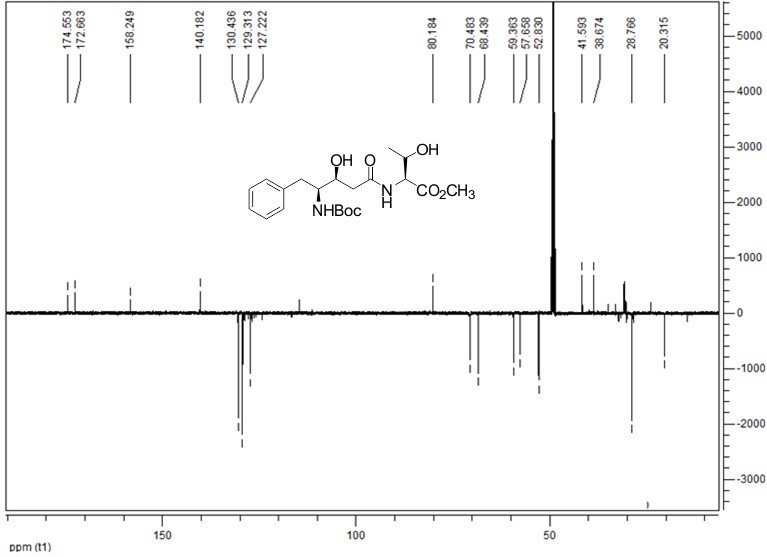


(7

c

)

ESI-HRMS


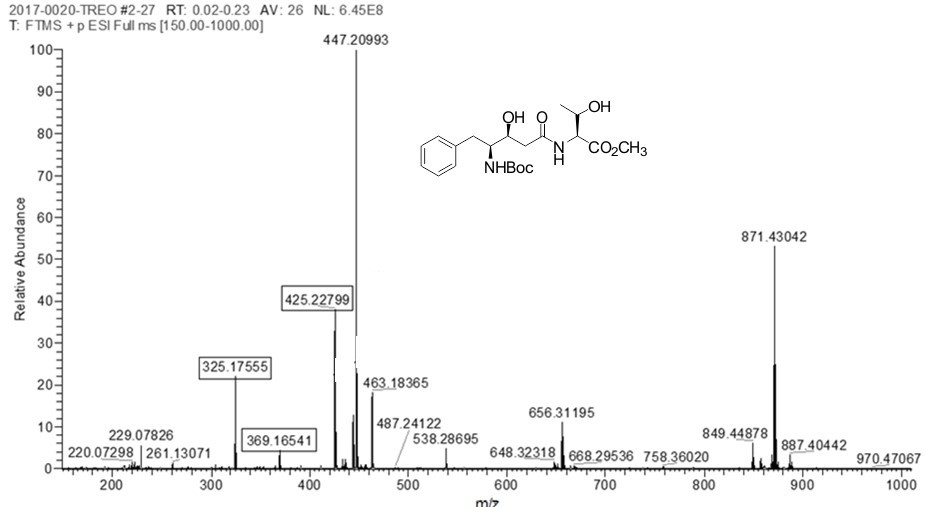


(7

c

)

# **Spectra of compound 7d**.

IR (KBr)


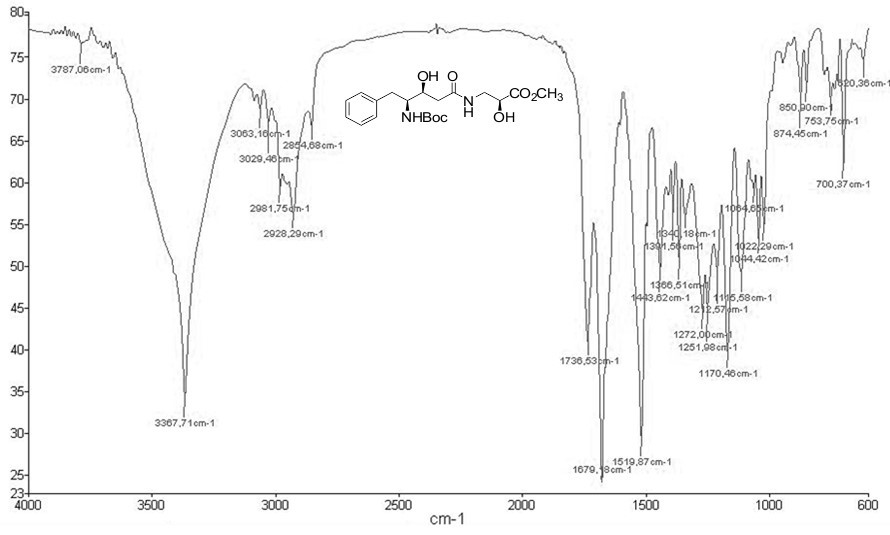


(7

d

)

^1^H NMR (500 MHz, MeOD)


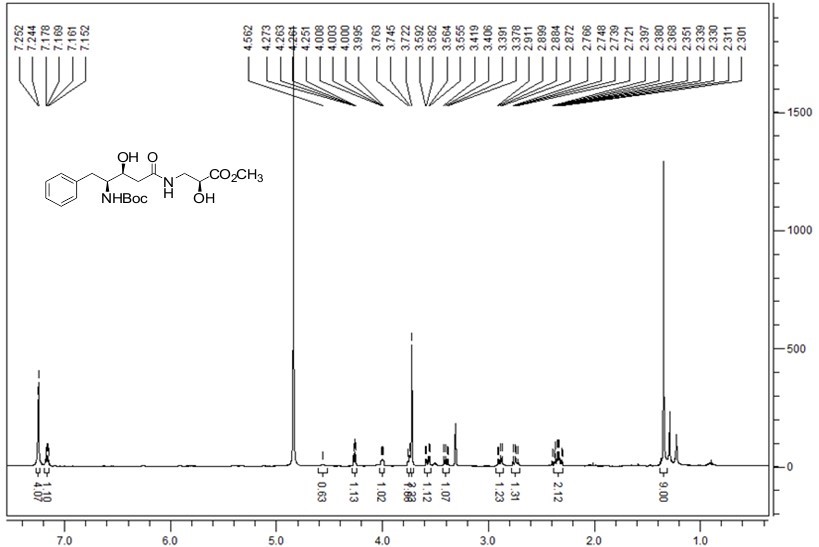


(7

d

)

APT (125.6 MHz, MeOD)


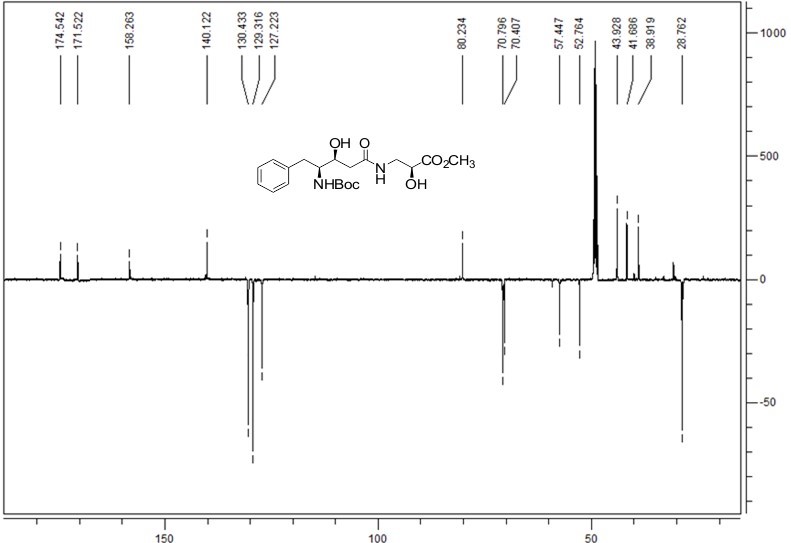


7

d

)

ESI-HRMS


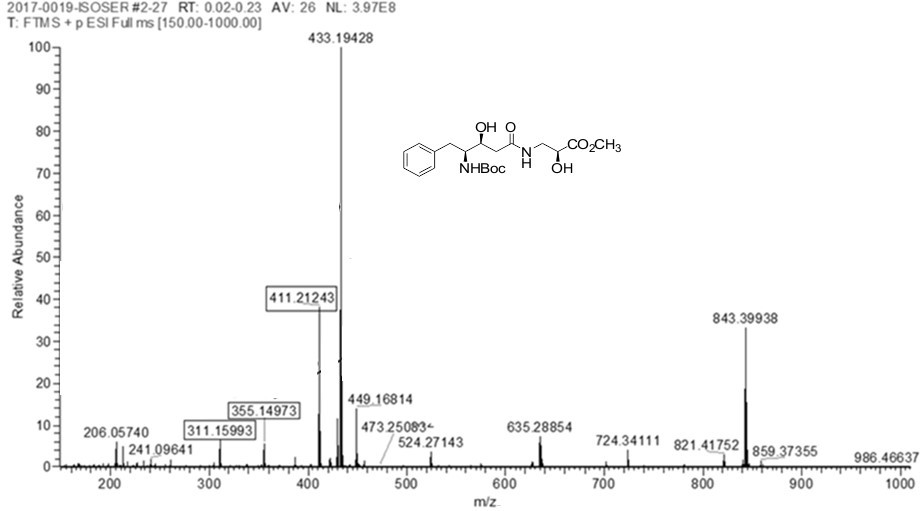


(7

d

)

# **Spectra of compound 7e**.

IR (KBr)


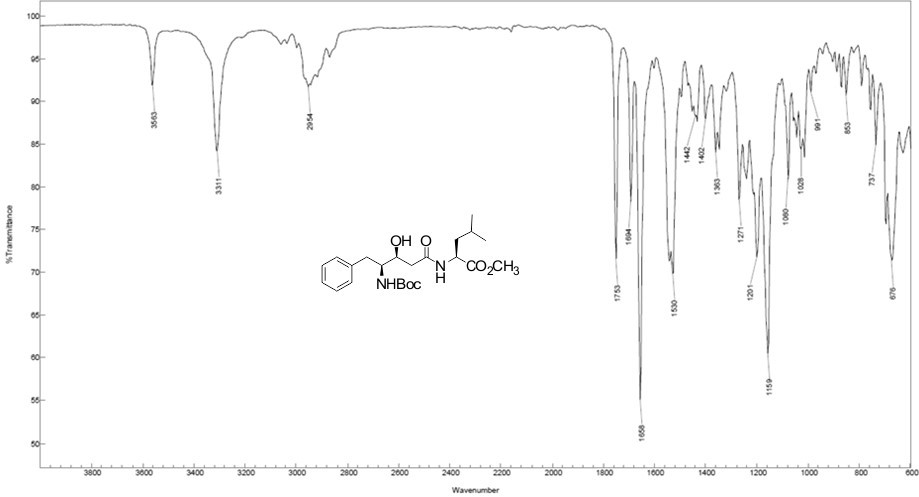


(7

e

)

(7

d

)

^1^H NMR (500 MHz, CD_3_COCD_3_)


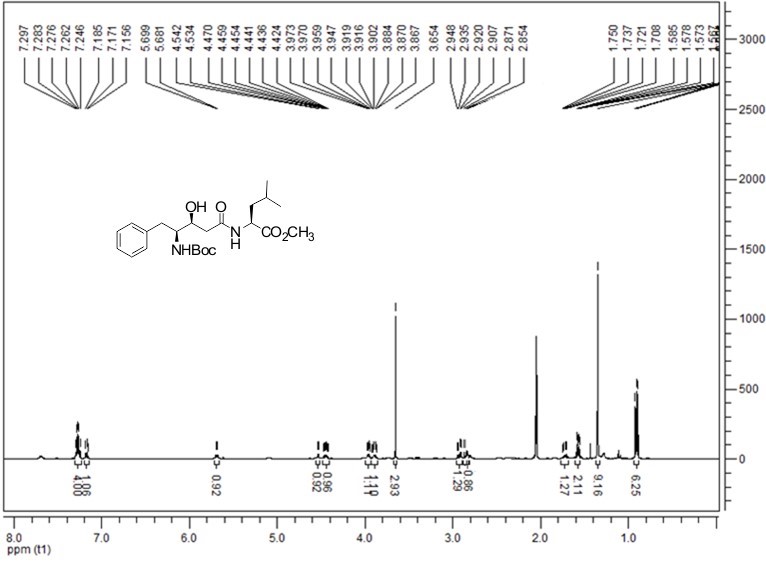


(7

e

)

APT (125.6 MHz, CD_3_COCD_3_)


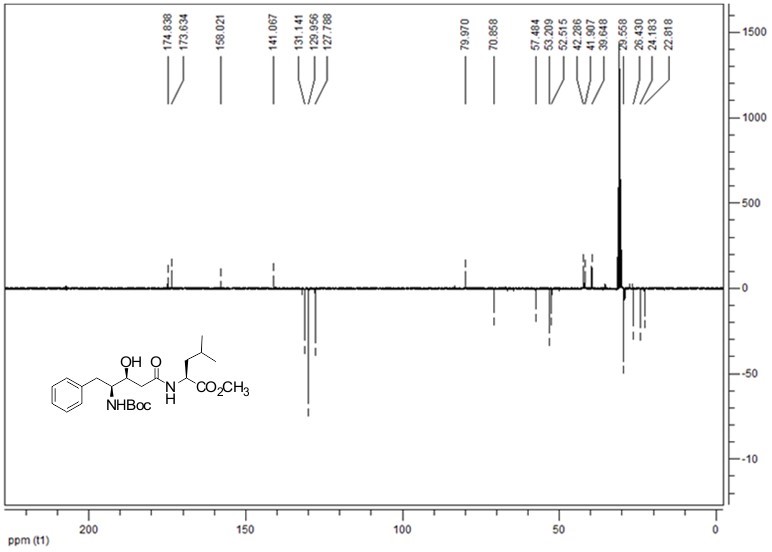


(7

e

)

HRMS-FAB


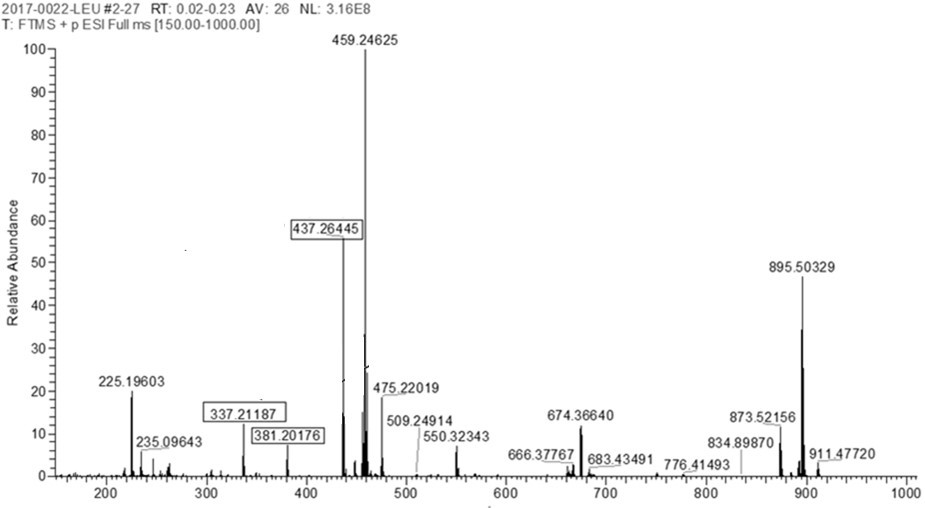


(7

e

)

# **Spectra of compound 7f**.

IR (KBr)


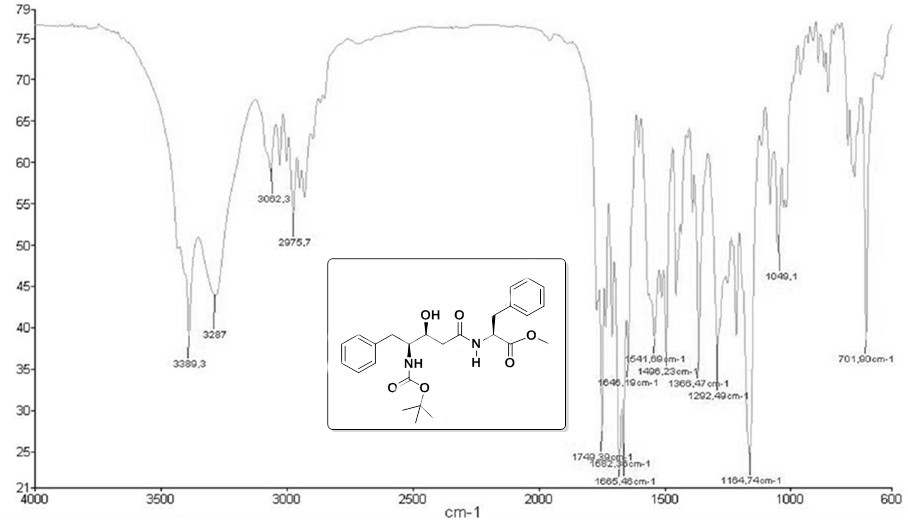


(7

f

)

(7

f

)

^1^H NMR (500 MHz, CDCl_3_)


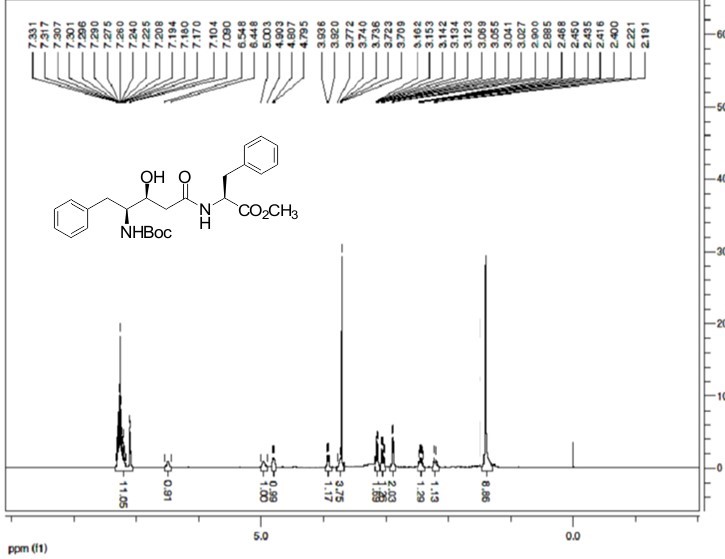


(7

f

)

APT (125.6 MHz, CDCl_3_)


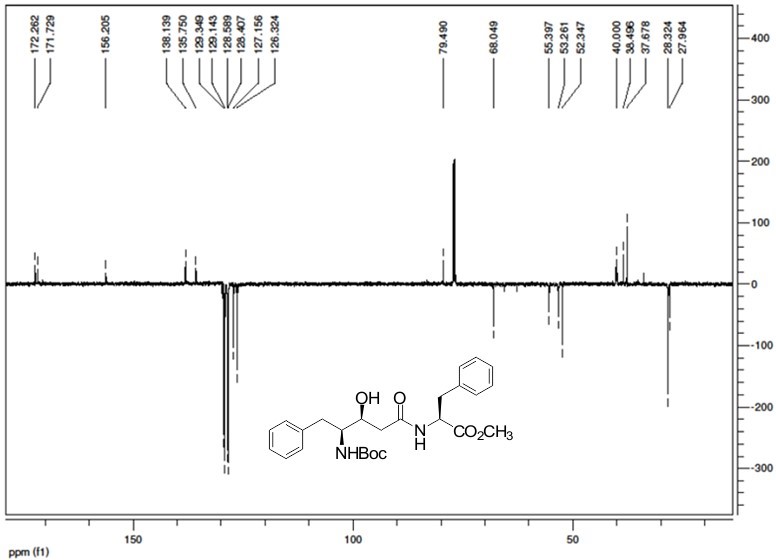


(7

f

)

HRMS-FAB


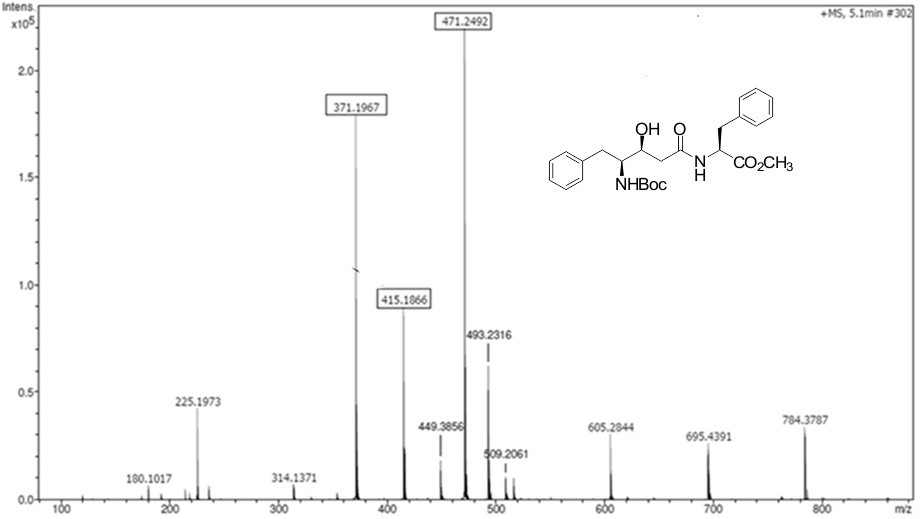


(7

f

)

# **Spectra of compound 7g**.

IR(KBr)


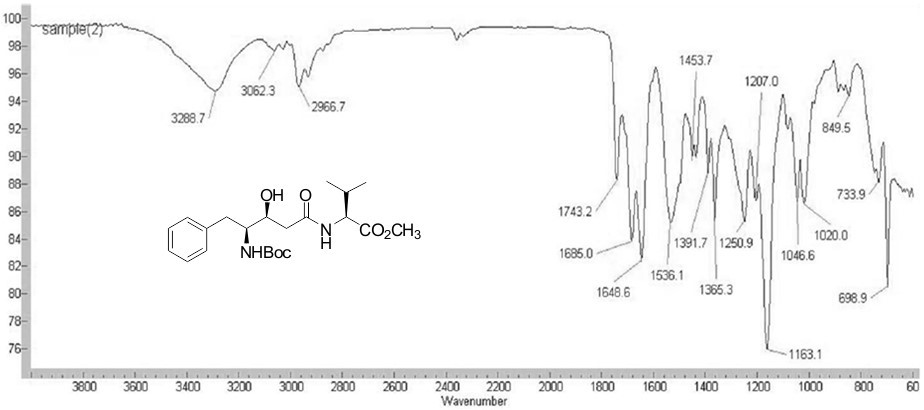


(7

g

)

^1^H NMR (500 MHz, CDCl_3_)


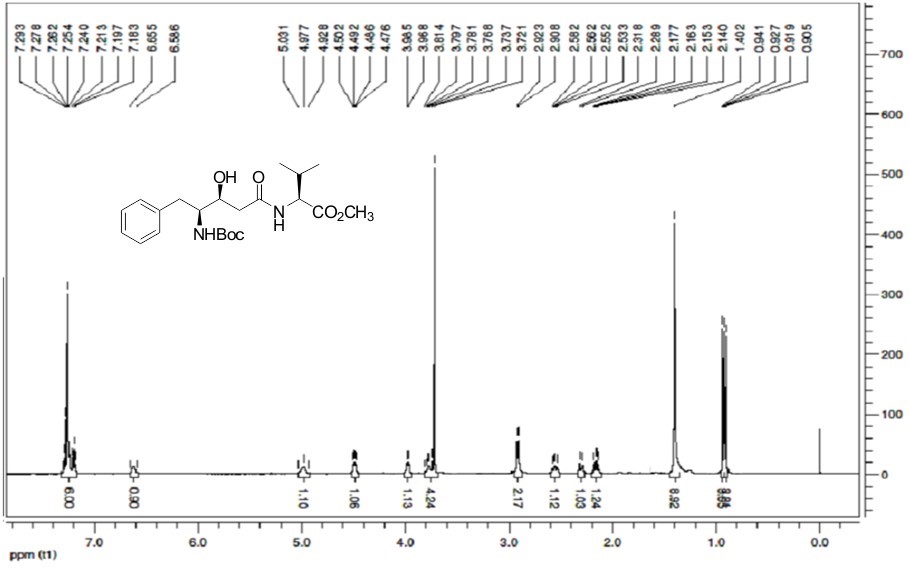


(7

g

)

APT (125.6 MHz, CDCl_3_)


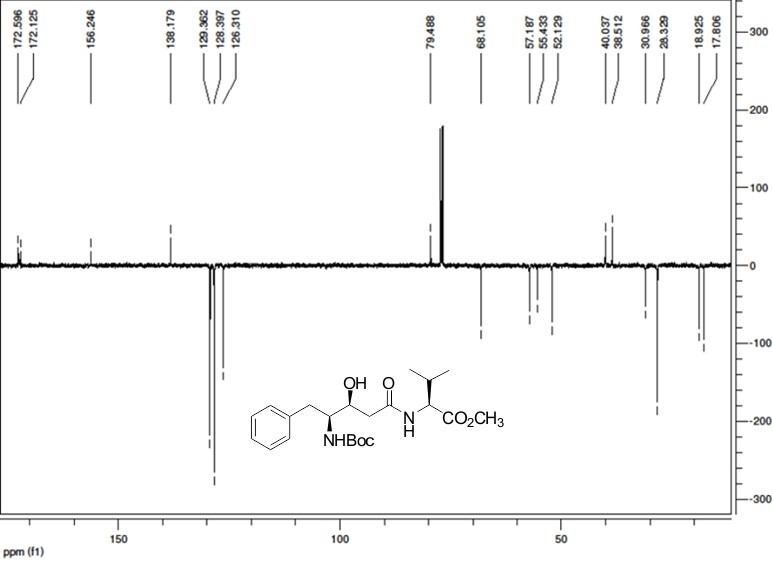


(7

g

)

HRMS-FAB


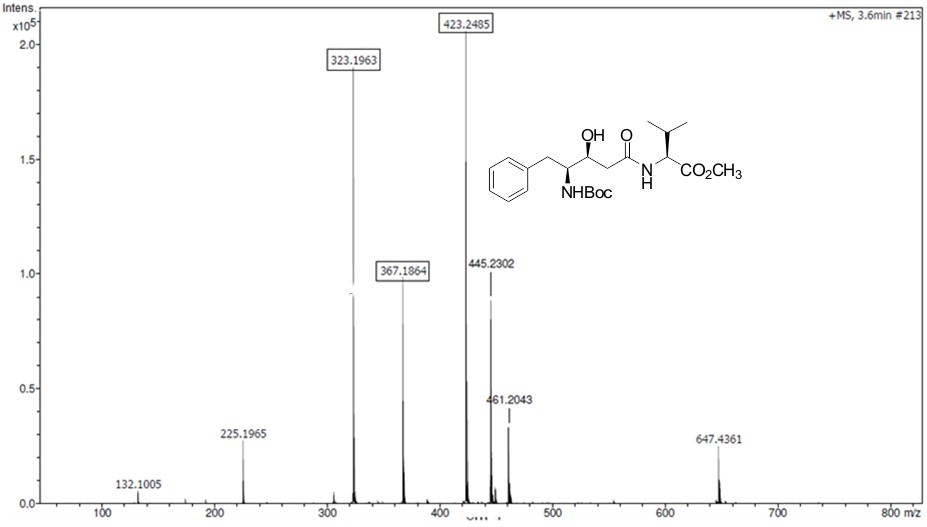


(7

g

)

# **Spectra of compound 7h**.

IR(KBr)


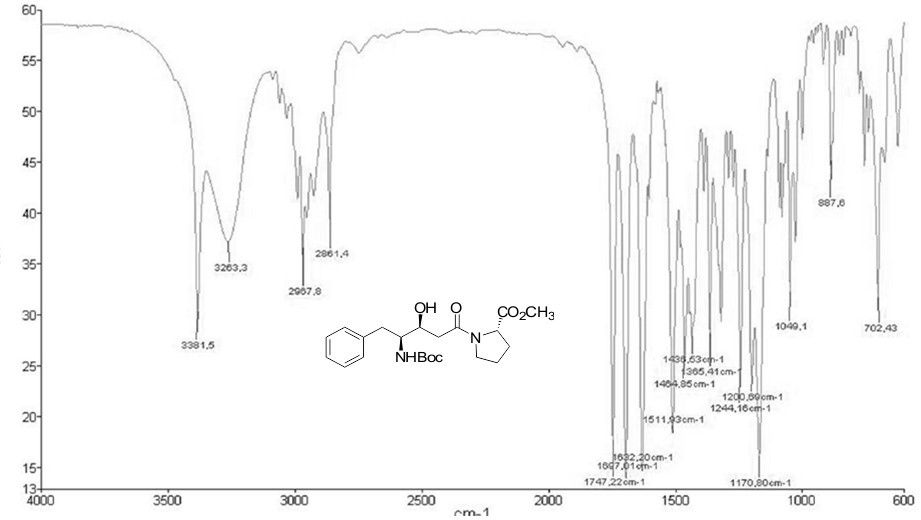


(7

h

)

^1^H NMR (500 MHz, CDCl_3_)


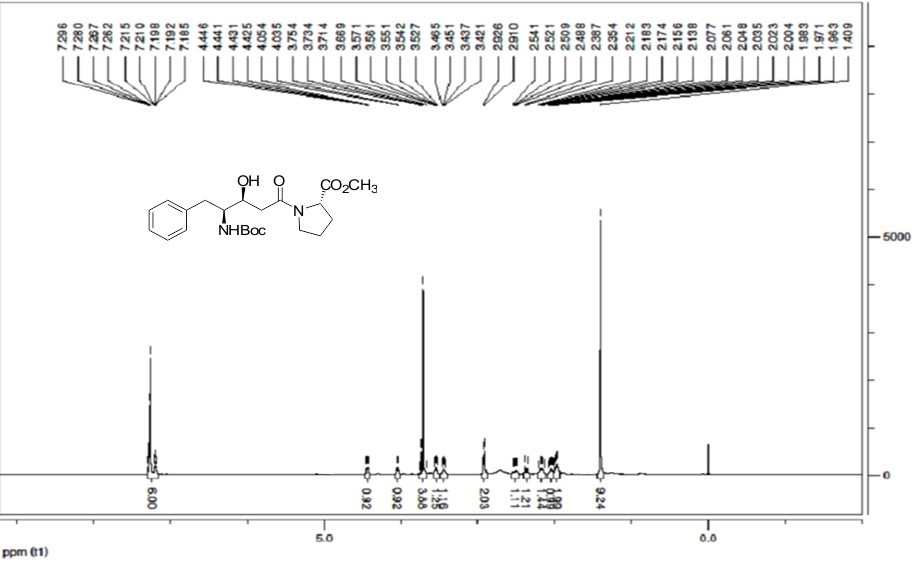


(7

h

)

APT (125.6 MHz, CDCl_3_)


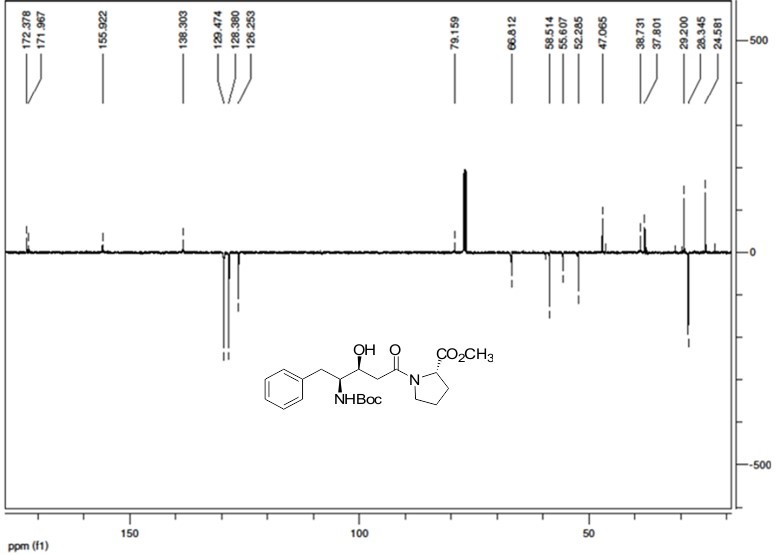


(7

h

)

HRMS-FAB


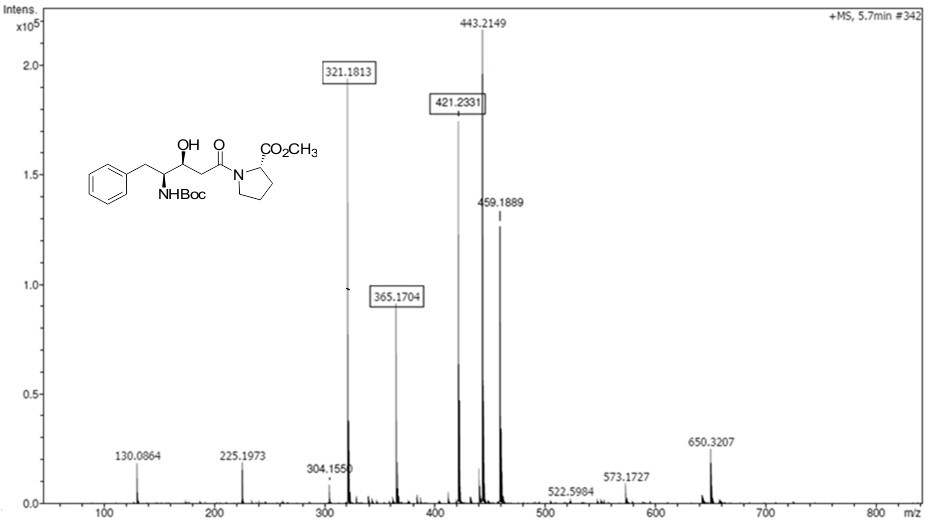


(7

h

)

# **Spectra of compound 8a**.

IR (KBr)


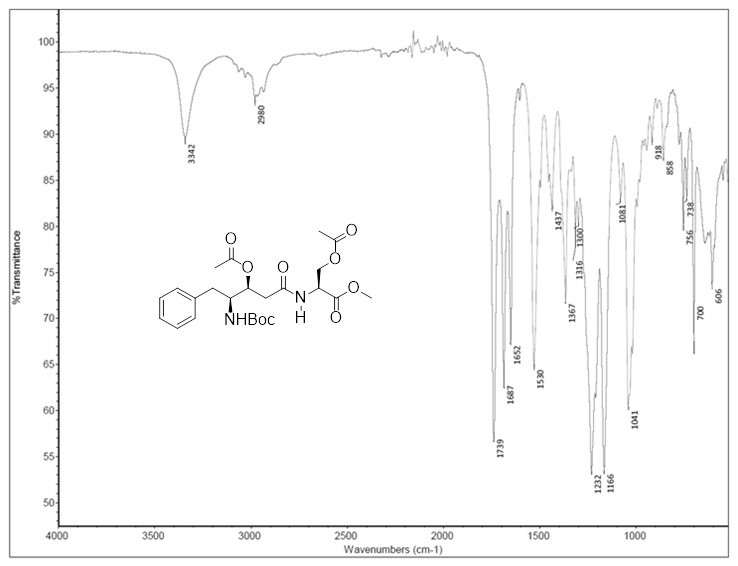


(8

a

)

^1^H NMR (500 MHz, CDCl_3_)


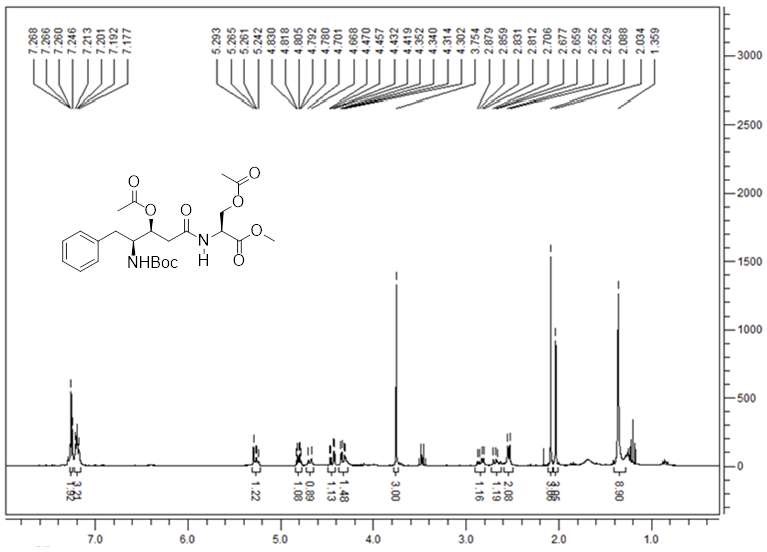


(8

a

)

APT (125.6 MHz, CDCl_3_)


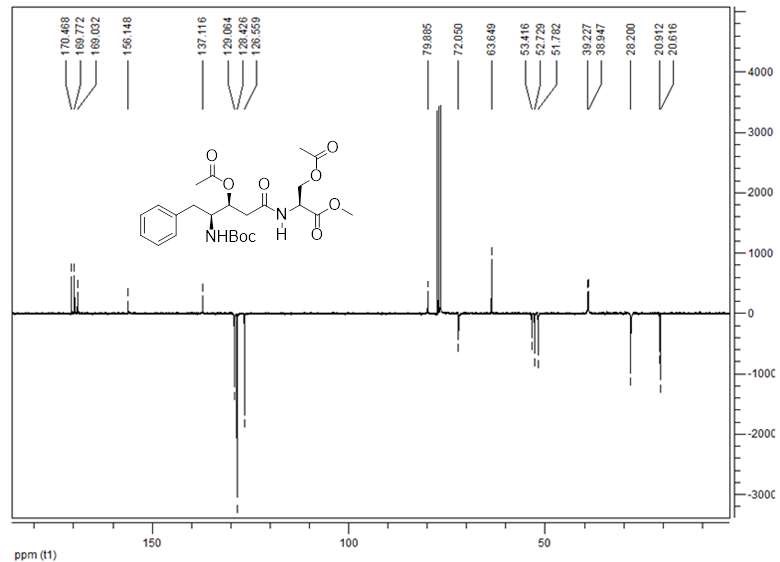


(8

a

)

ESI-HRMS


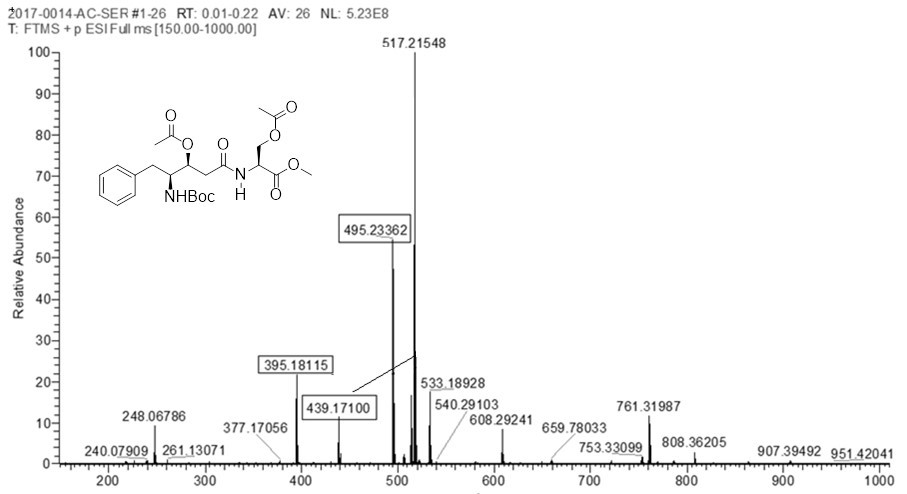


(8

a

)

# **Spectra of compound 8b**.

IR (KBr)


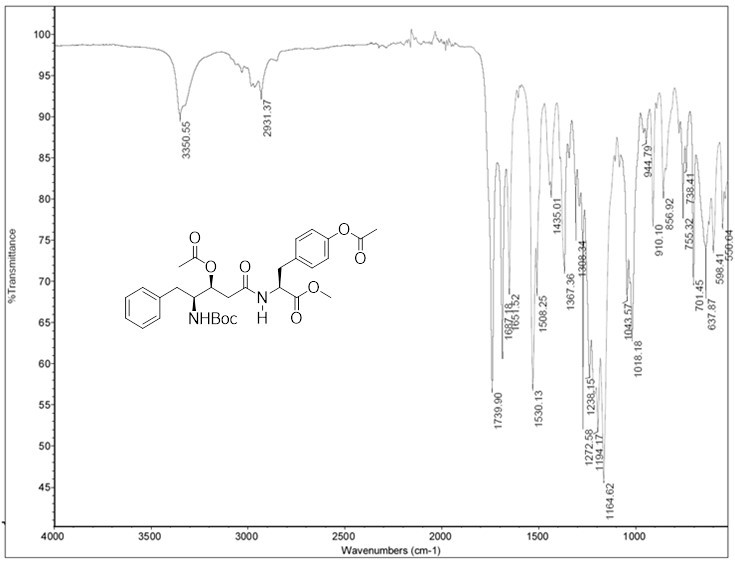


(8

b

)

^1^H NMR (500 MHz, CDCl_3_)


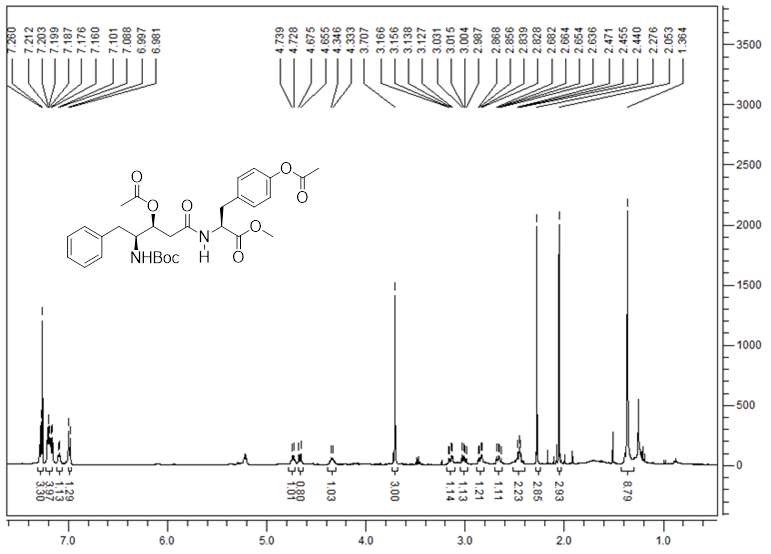


(8

b

)

APT (125.6 MHz, CDCl_3_)


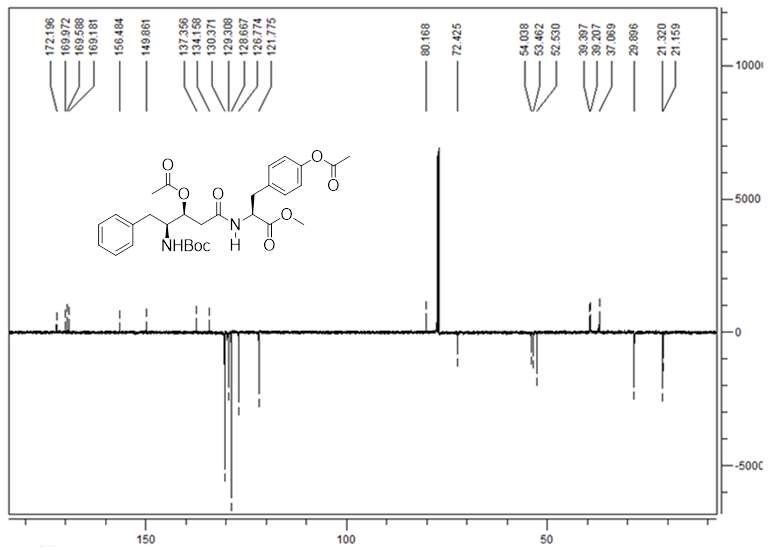


(8

b

)

ESI-HRMS


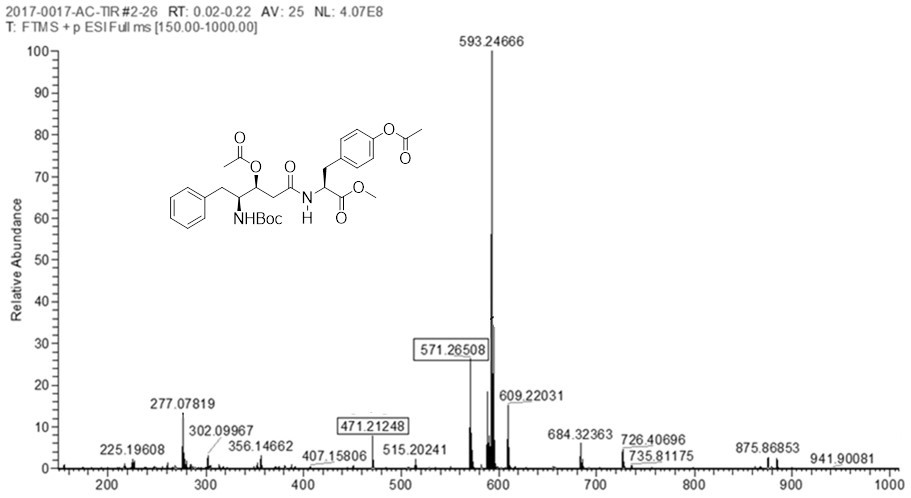


(8

b

)

# **Spectra of compound 8c**.

IR (KBr)


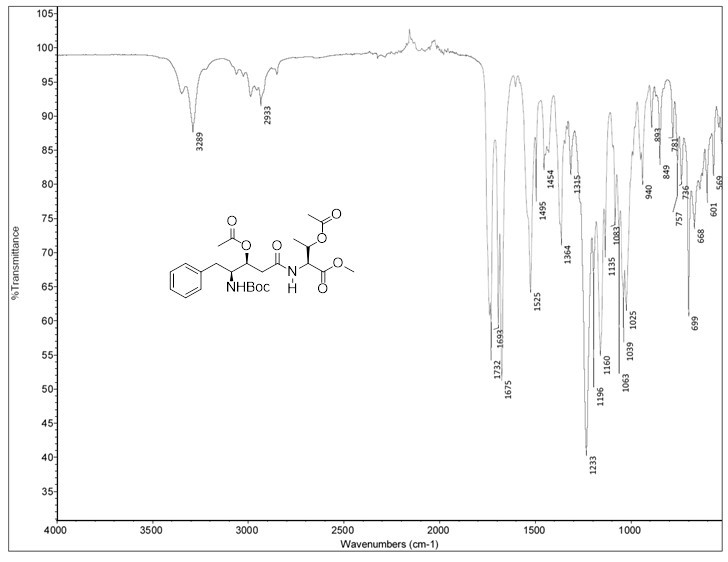


(8

c

)

^1^H NMR (500 MHz, CDCl_3_)


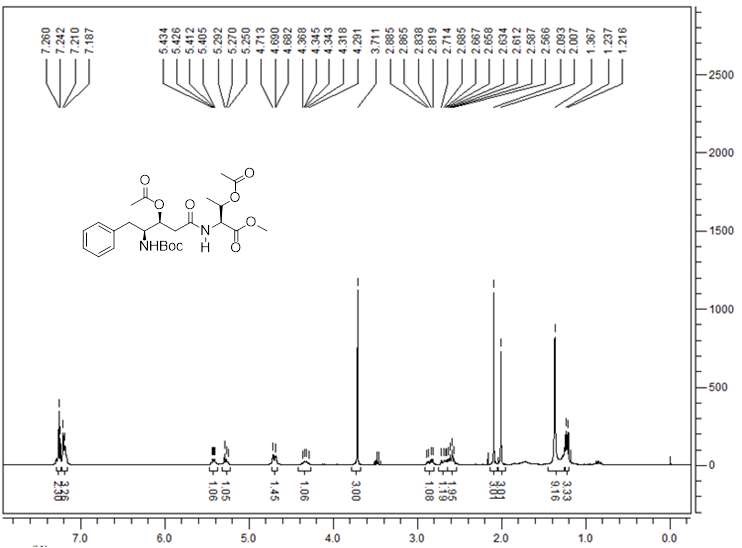


(8

c

)

APT (125.6 MHz, CDCl_3_)


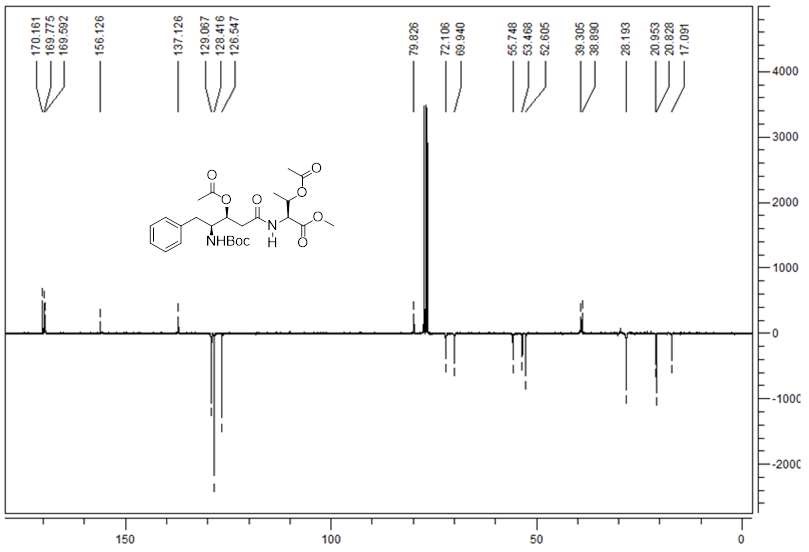


(8

c

)

ESI-HRMS


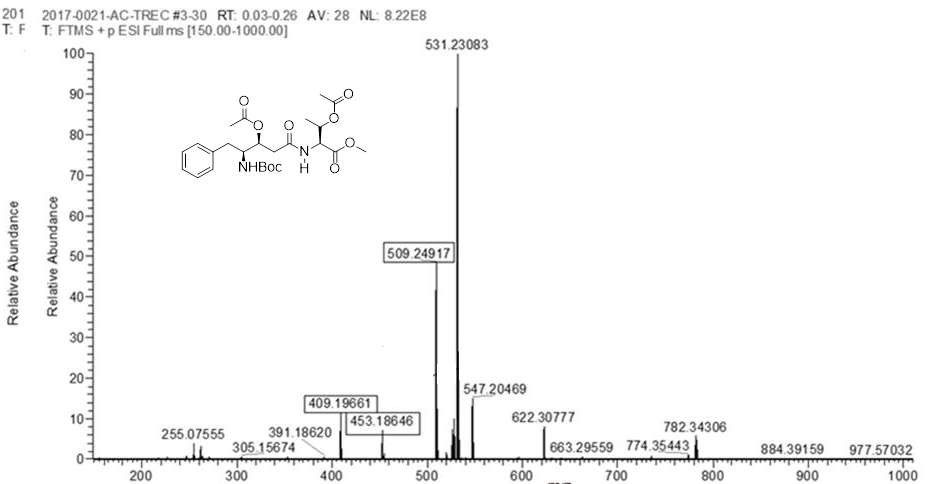


(8

c

)

**Spectra of compound 8d**.

IR (KBr)


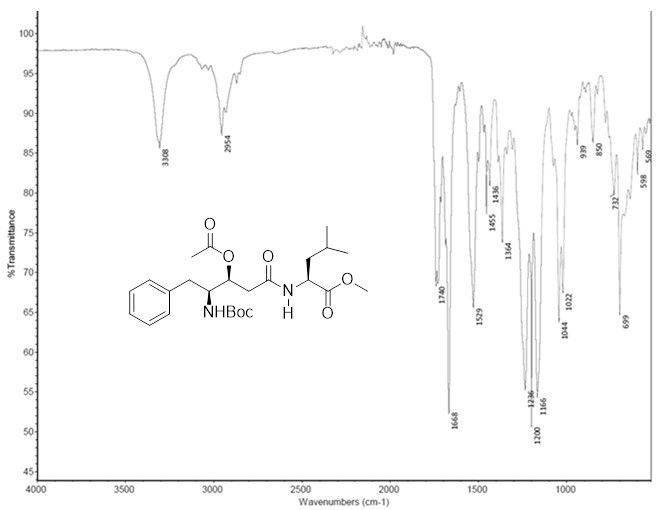


(8

d

)

^1^H NMR (500 MHz, CDCl_3_)


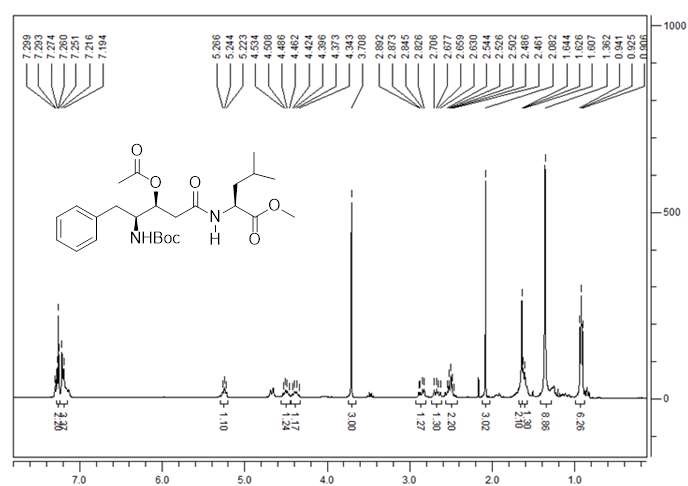


(8

d

)

APT (125.6 MHz, CDCl_3_)


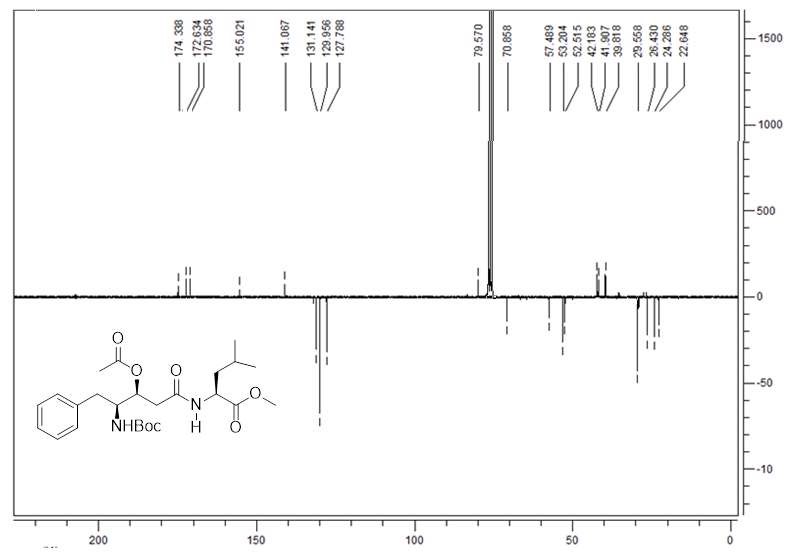


(8

d

)

ESI-HRMS


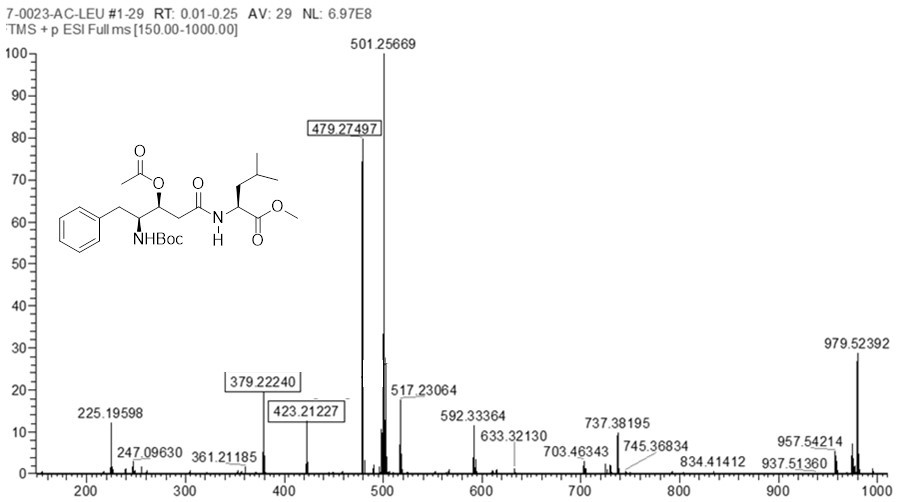


(8

d

)

# **Spectra of compound 8e**.

IR (KBr)


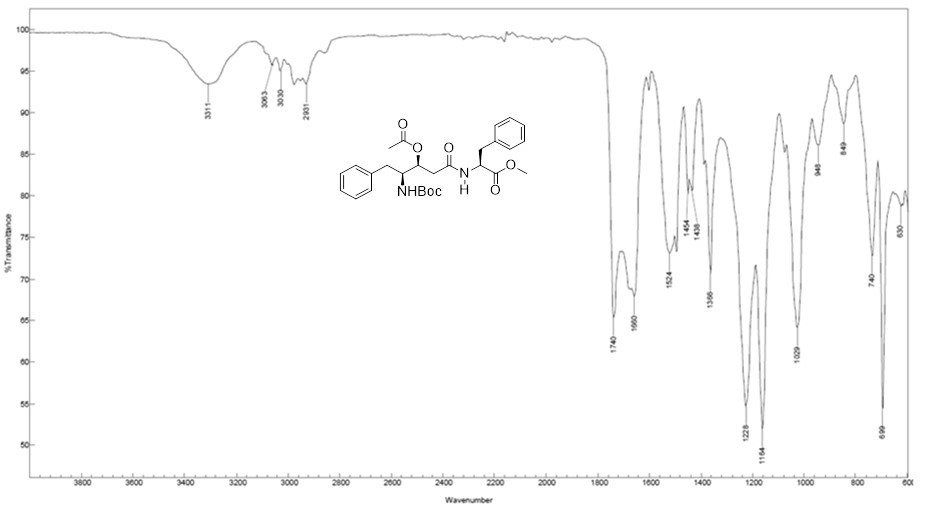


(8

e

)

^1^H NMR (500 MHz, CDCl_3_)


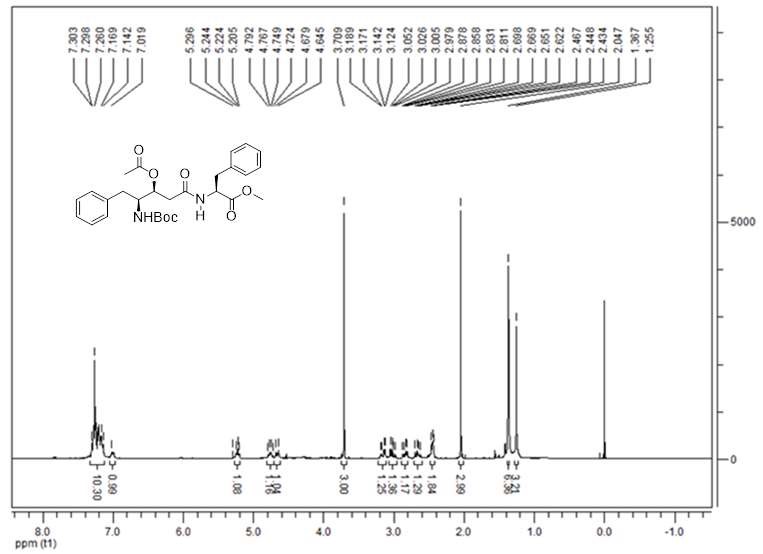


(8

e

)

APT (125.6 MHz, CDCl_3_)


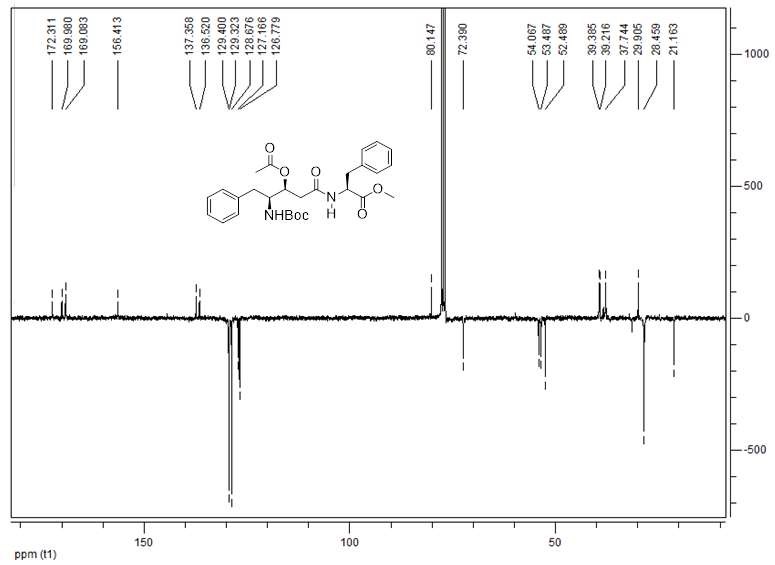


(8

e

)

ESI-HRMS


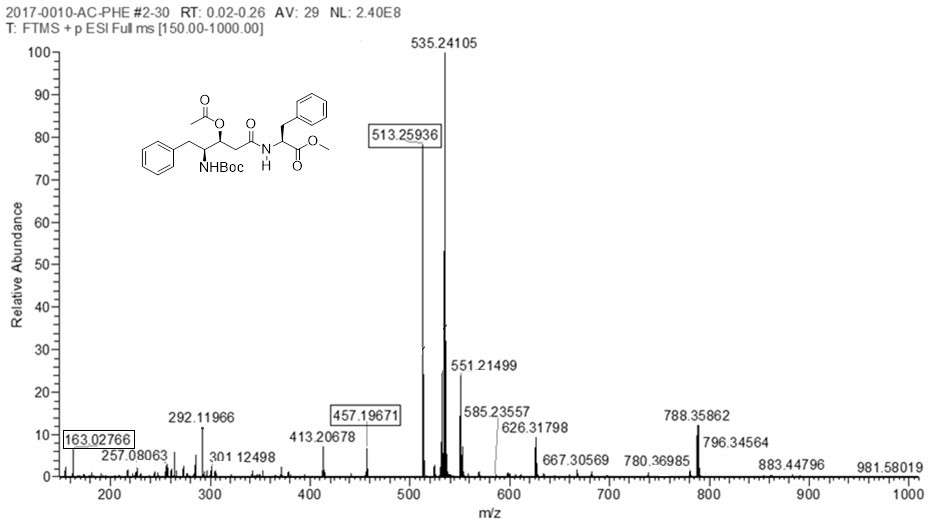


(8

e

)

# **Spectra of compound 8f**.

IR (KBr)


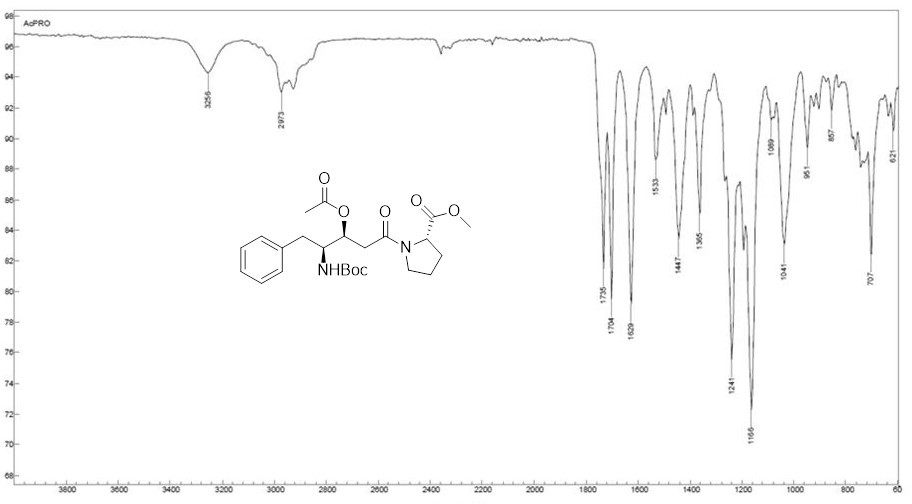


(8

f

)

^1^H NMR (500 MHz, CDCl_3_)


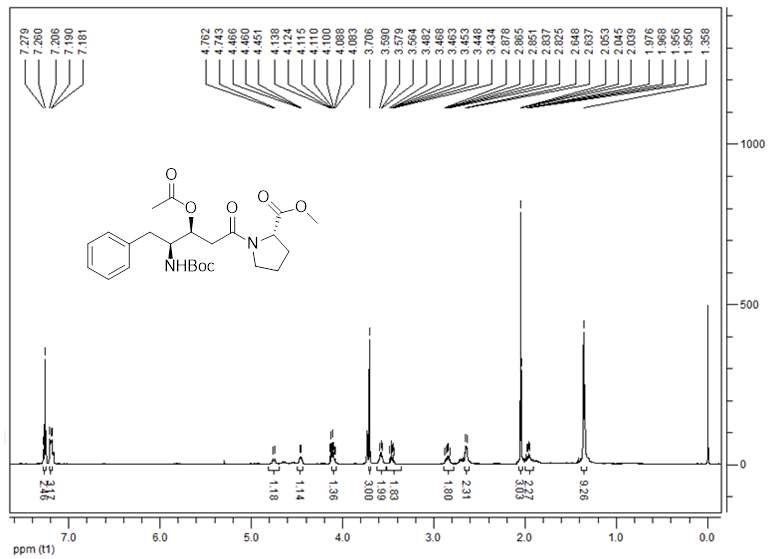


(8

f

)

APT (125.6 MHz, CDCl_3_)


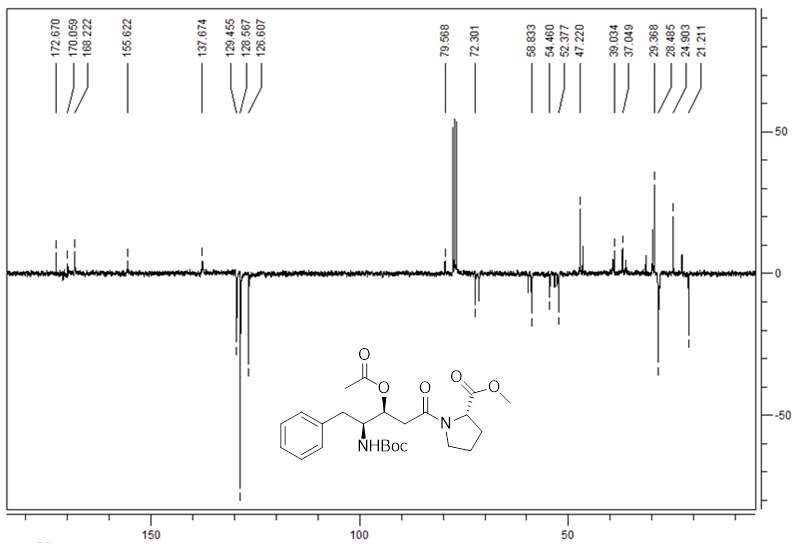


(8

f

)

ESI-HRMS


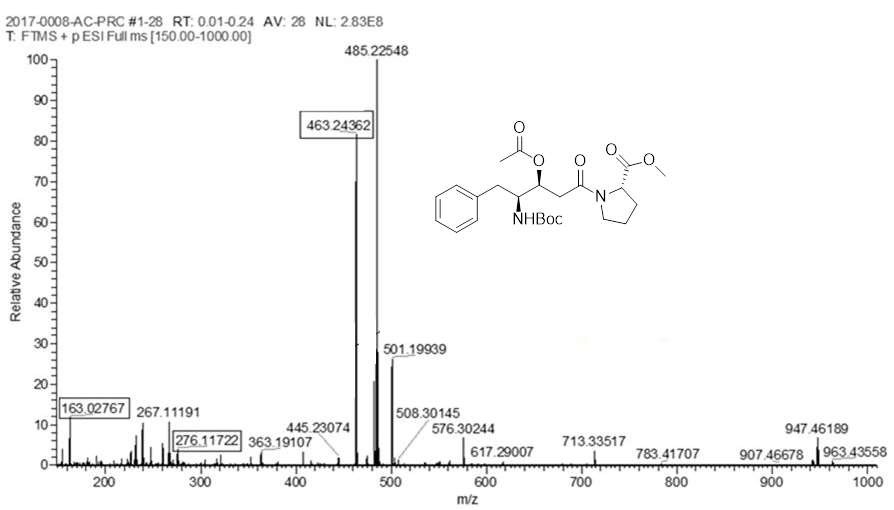


(8

f

)

# **Spectra of compound 8g**.

IR (KBr)


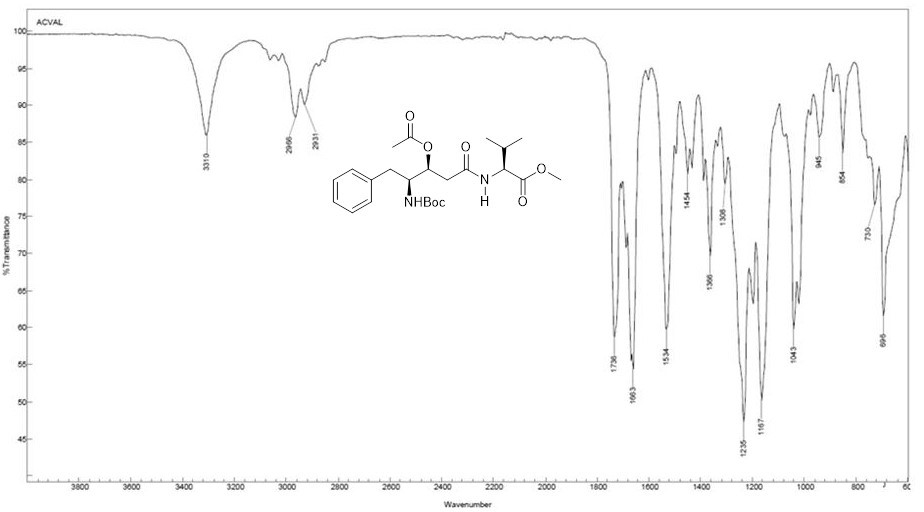


(8

g

)

^1^H NMR (500 MHz, CDCl_3_)


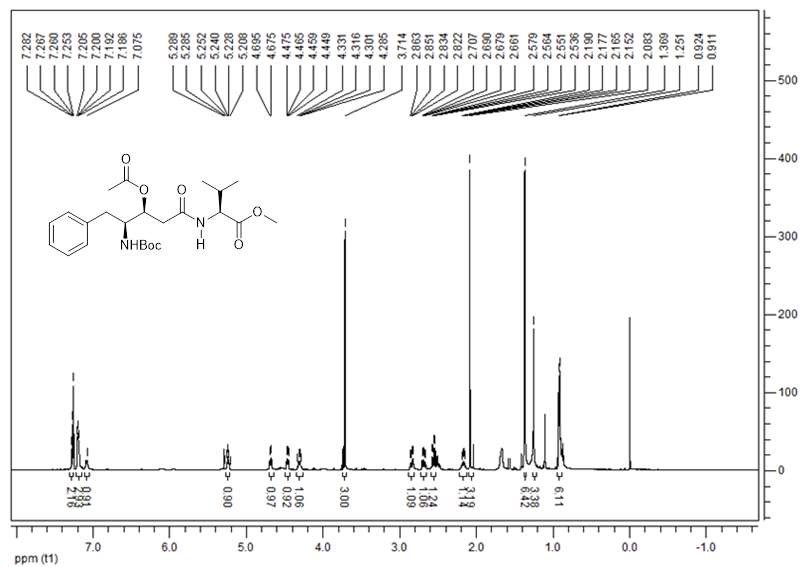


(8

g

)

APT (125.6 MHz, CDCl_3_)


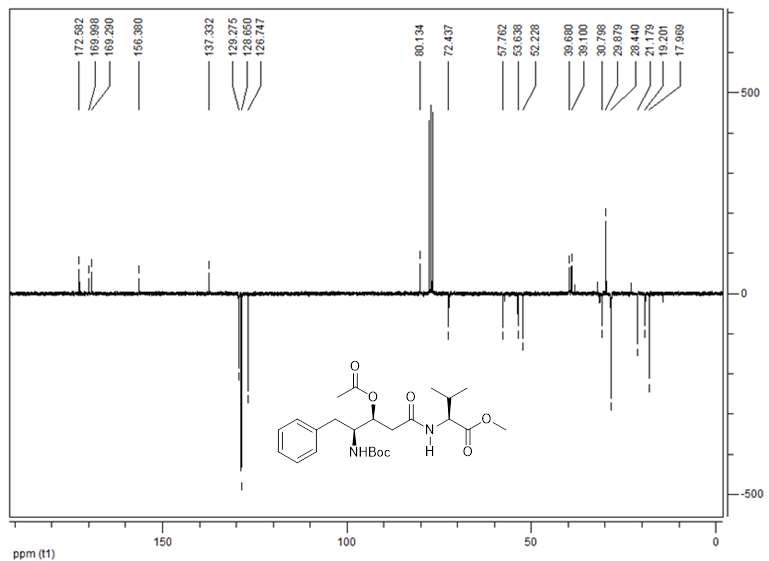


(8

g

)

ESI-HRMS


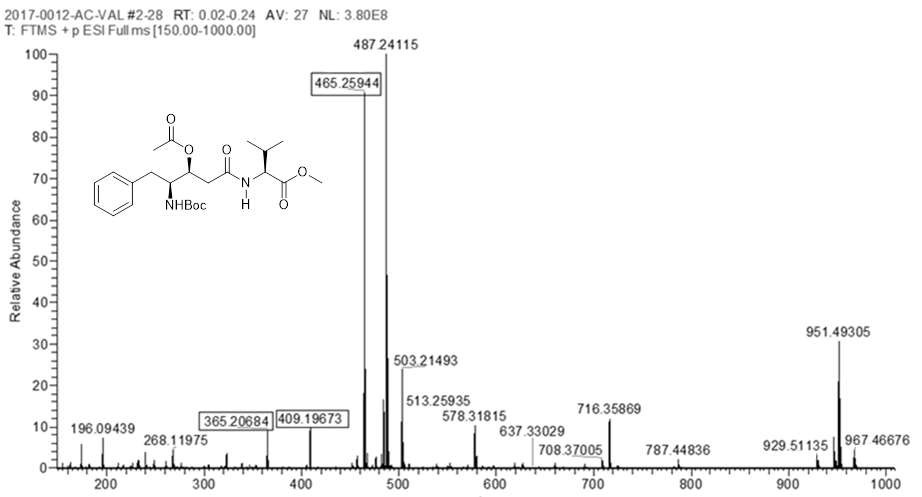


(8

g

)

# **Spectra of compound 9a**.

^1^H NMR (500 MHz, MeOD)


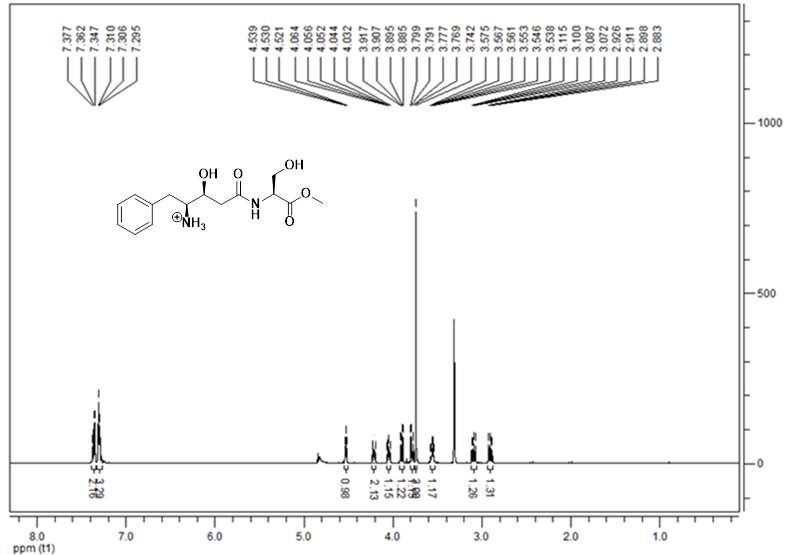


(9

a

)

APT (125.6 MHz, MeOD)


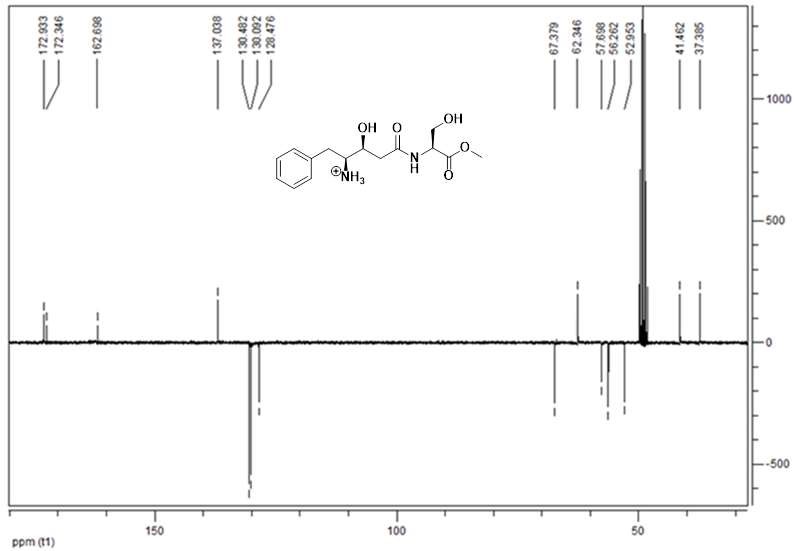


(9

a

)

ESI-HRMS


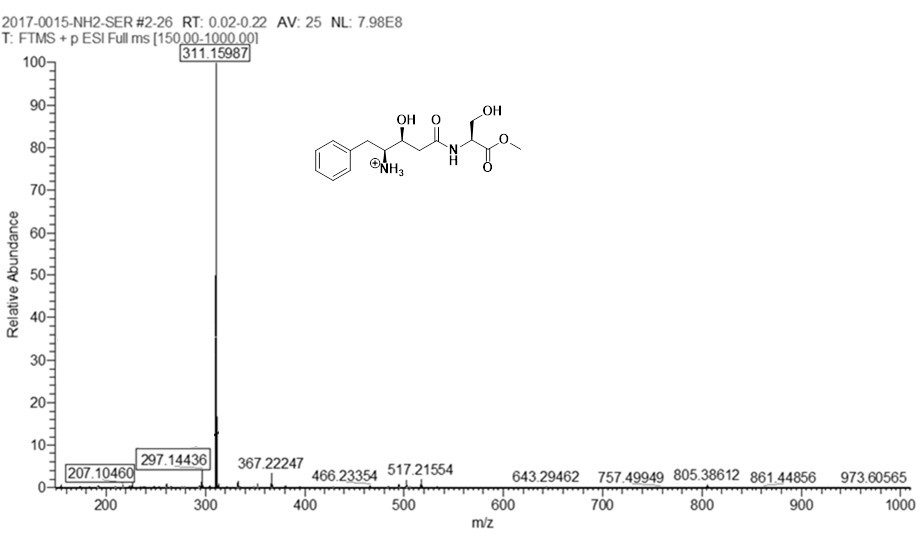


(9

a

)

# **Spectra of compound 9b**.

IR (KBr)


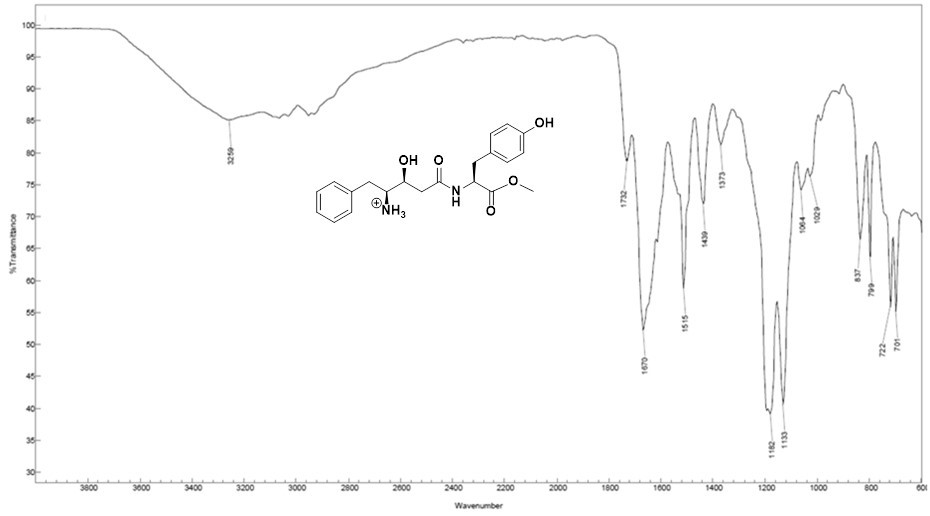


(9

b

)

^1^H NMR (500 MHz, MeOD)


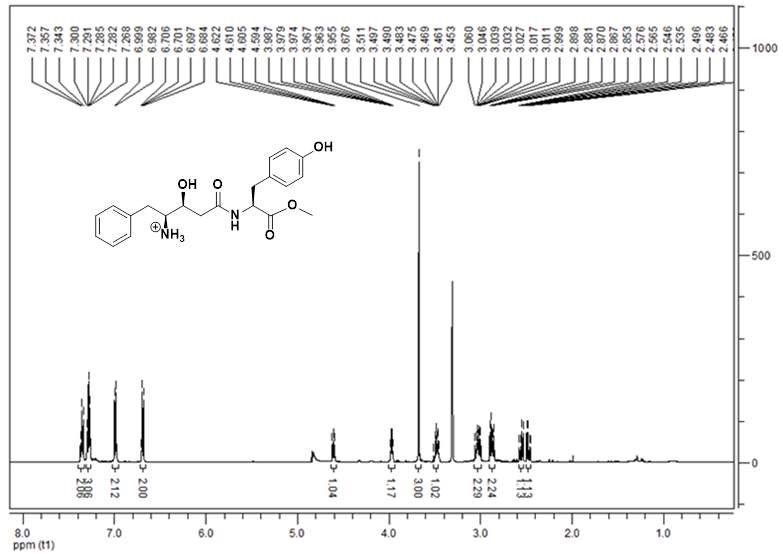


(9

b

)

APT (125.6 MHz, MeOD)


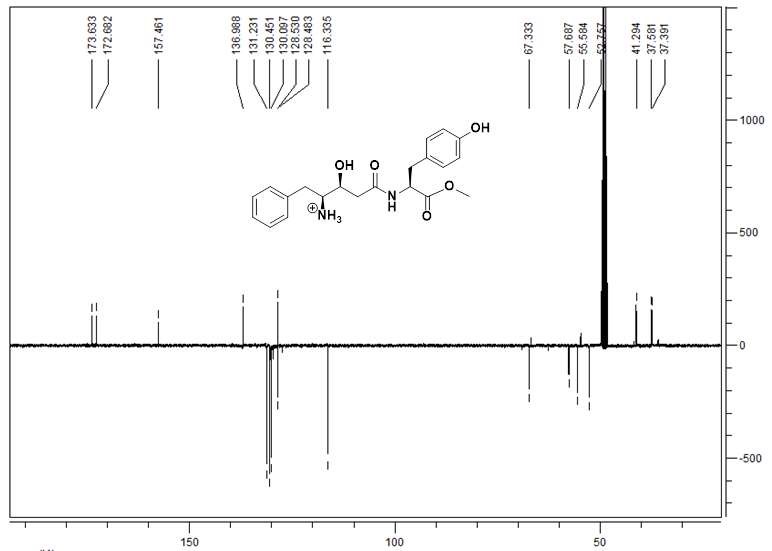


(9

b

)

ESI-HRMS


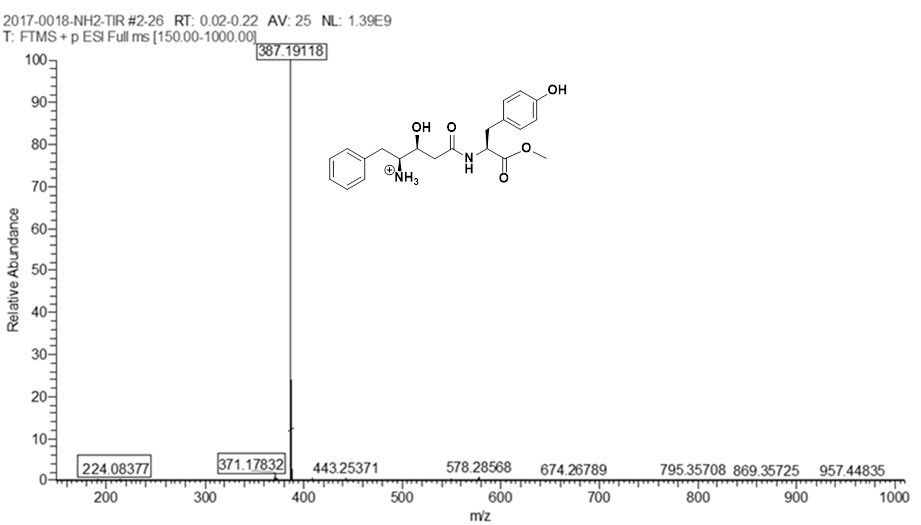


(9

b

)

# **Spectra of compound 9c**.

IR (KBr)


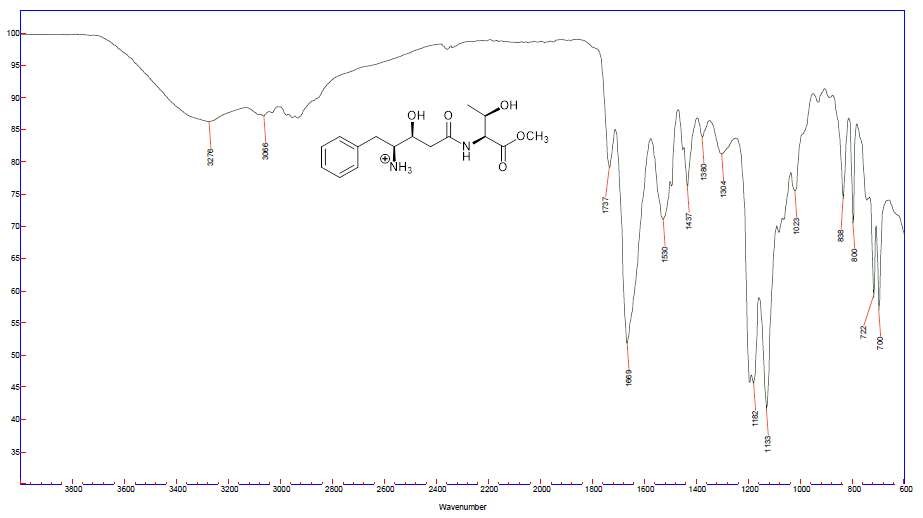


(9

c

)

^1^H NMR (500 MHz, MeOD)


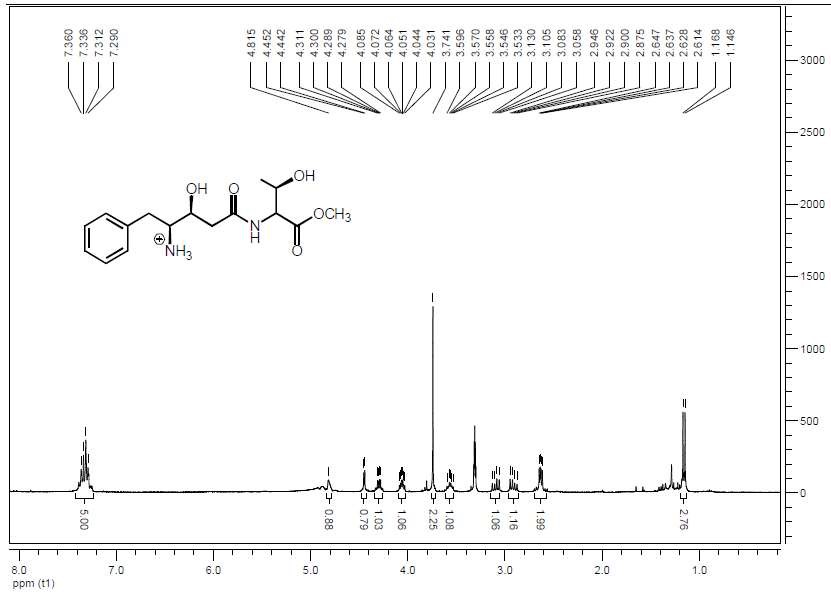


(9

c

)

APT (125.6 MHz, MeOD)


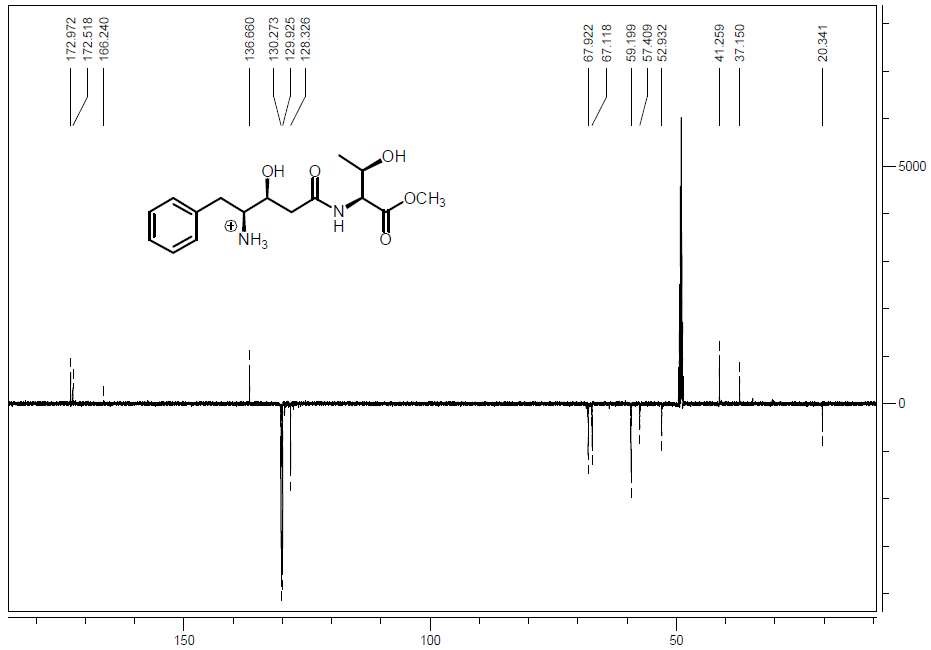


(9

c

)

ESI-HRMS


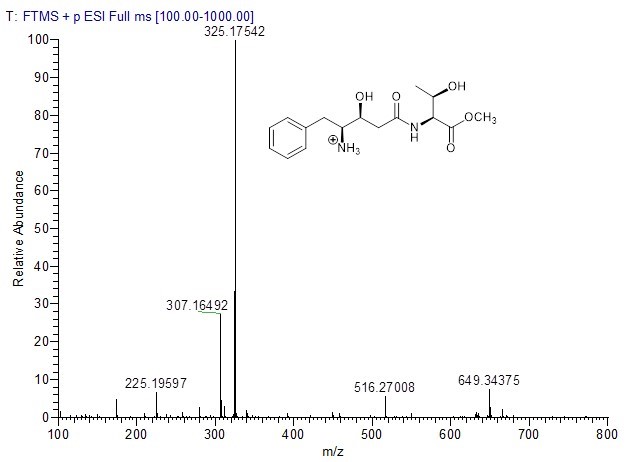


(9

c

)

# **Spectra of compound 9d**.

IR (KBr)


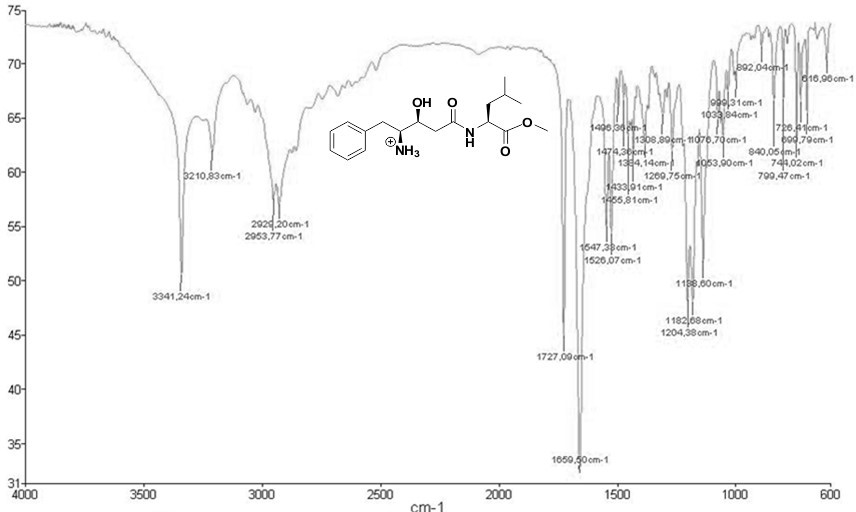


(9

d

)

^1^H NMR (500 MHz, MeOD)


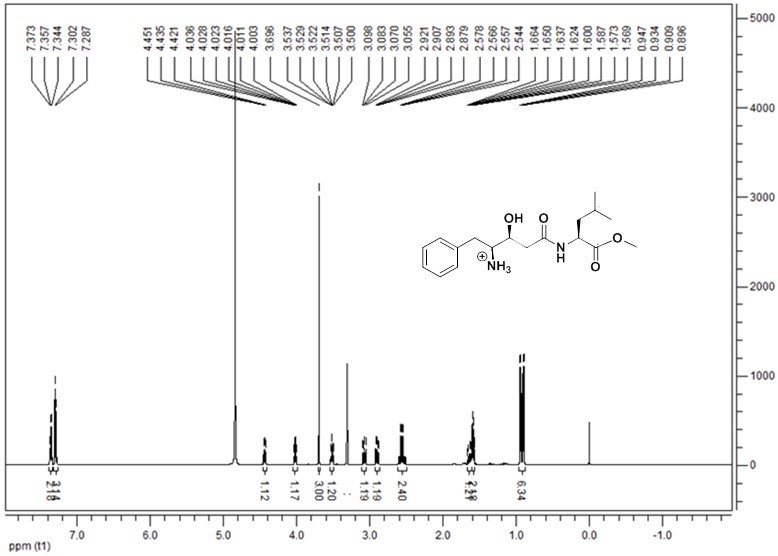


(9

d

)

APT (125.6 MHz, MeOD)


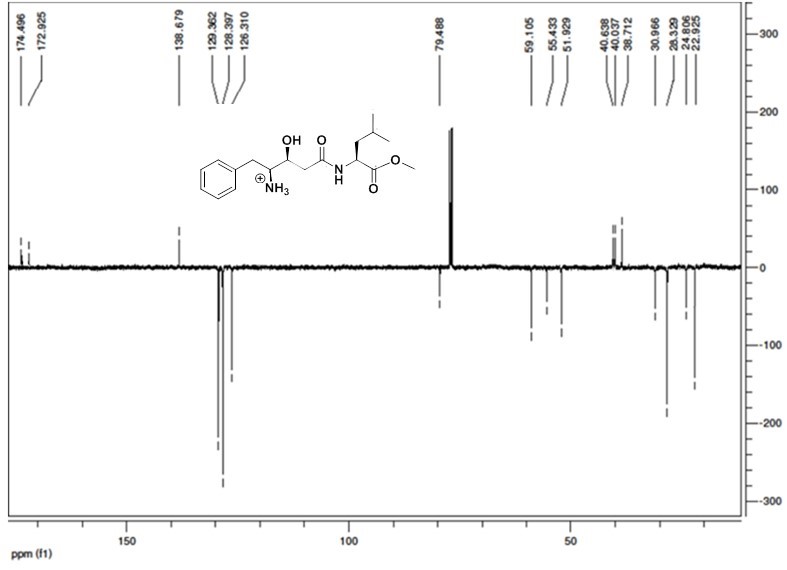


(9

d

)

ESI-HRMS


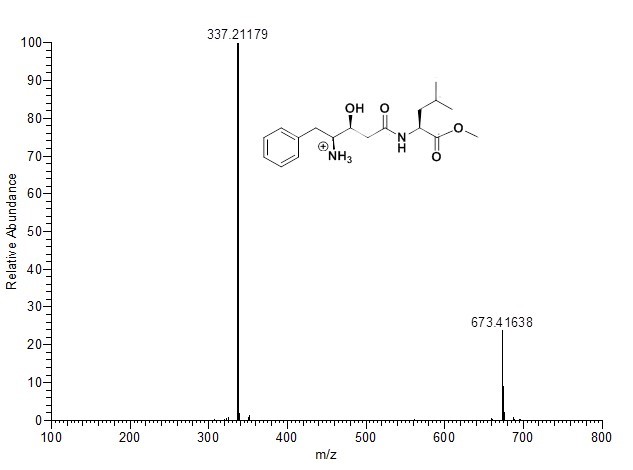


(9

d

)

# **Spectra of compound 9e**.

IR (KBr)


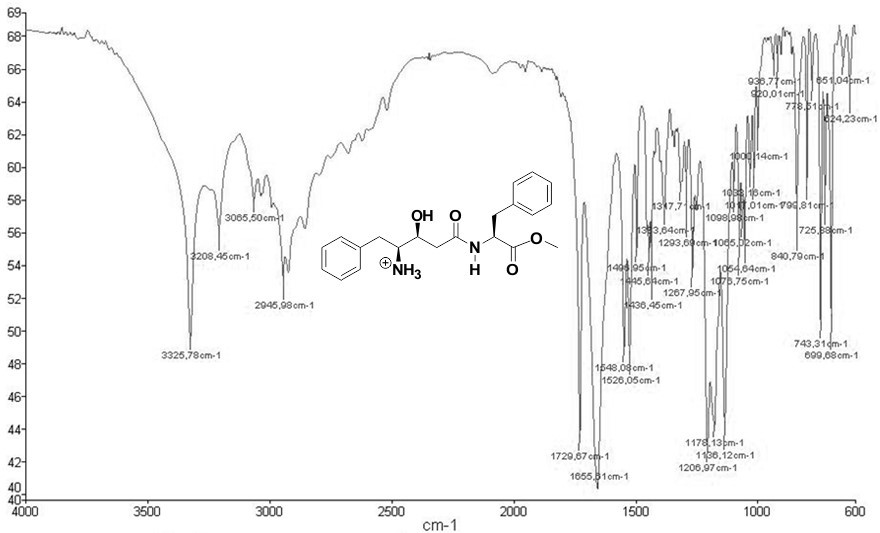


(9

e

)

^1^H NMR (500 MHz, DMSO-d_6_)


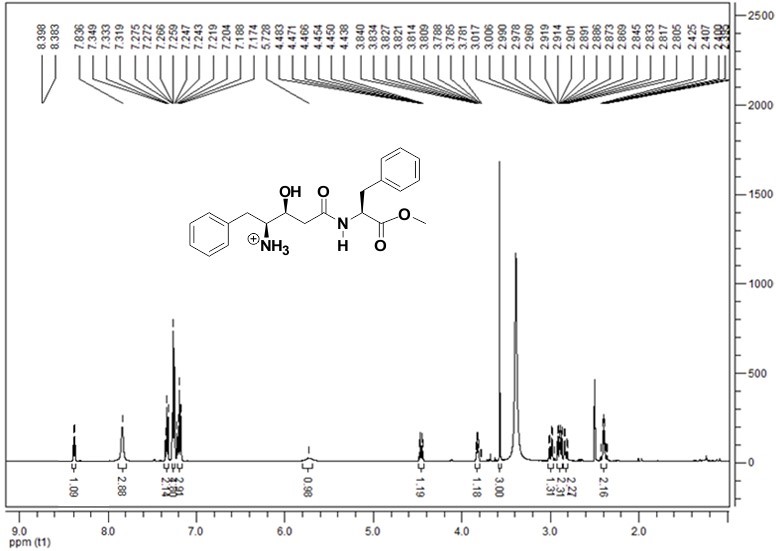


(9

e

)

APT (125.6 MHz, DMSO-d_6_)


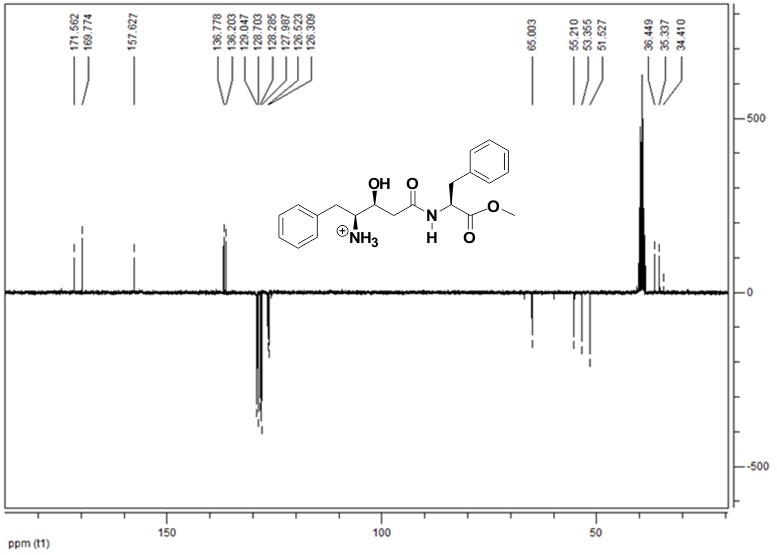


(9

e

)

ESI-HRMS


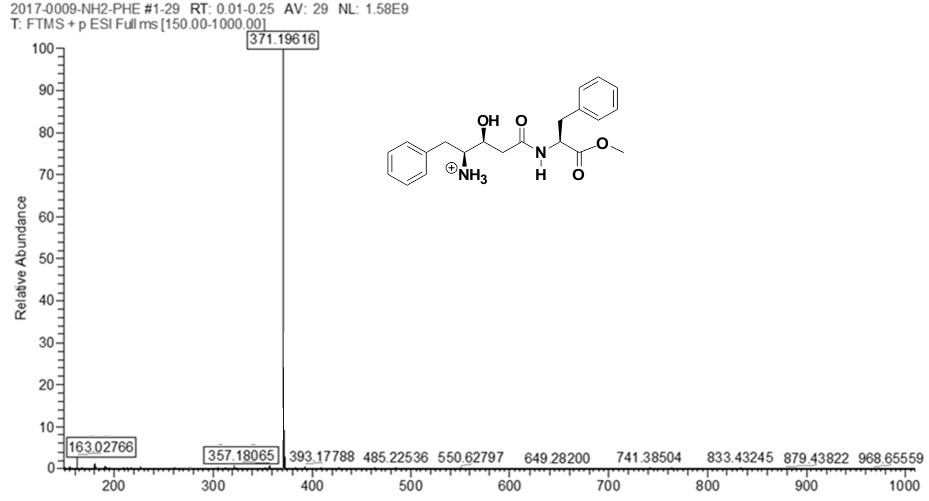


(9

e

)

# **Spectra of compound 9f**.

IR (KBr)


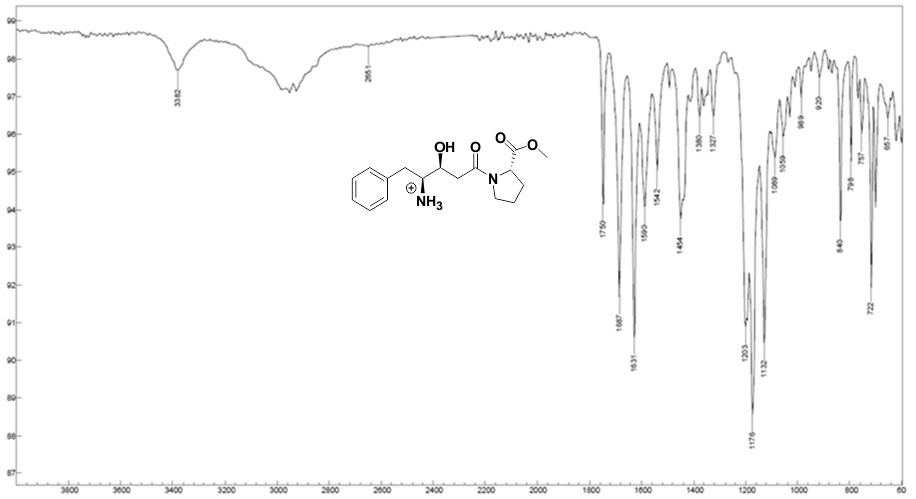


(9

f

)

^1^H NMR (500 MHz, DMSO-d_6_)


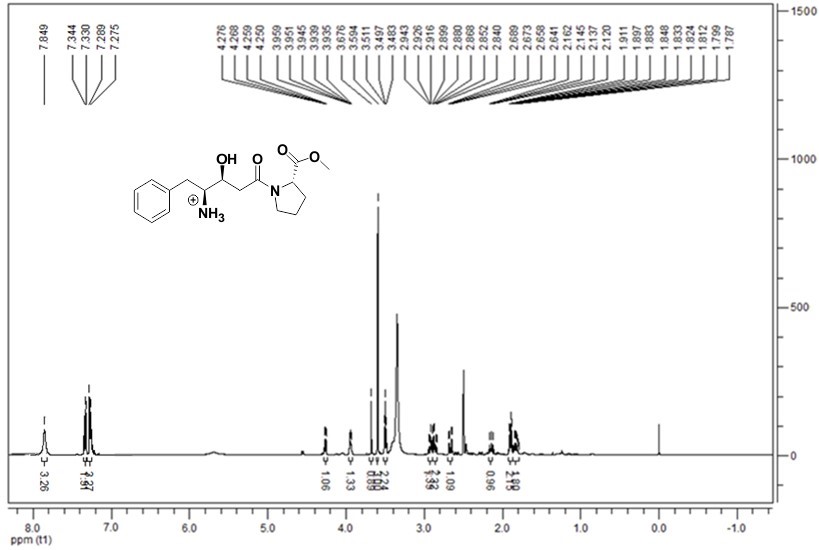


(9

f

)

APT (125.6 MHz, DMSO-d_6_)


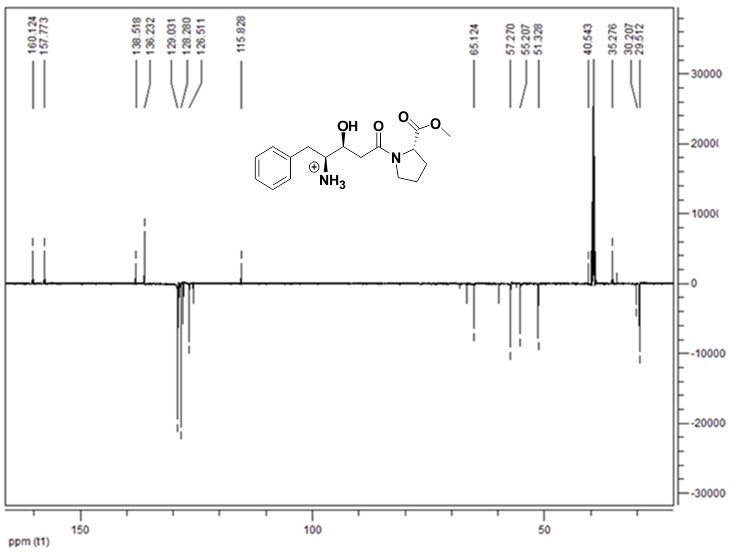


(9

f

)

ESI-HRMS


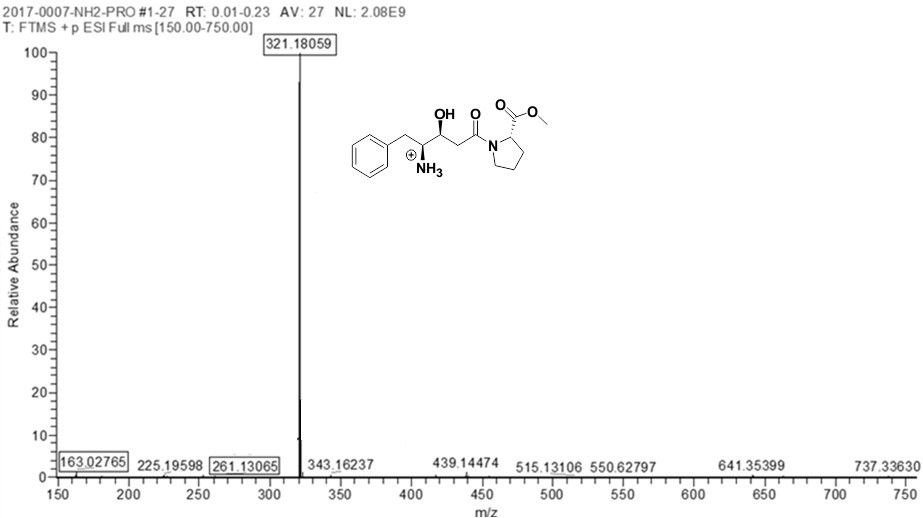


(9

f

)

# **Spectra of compound 9g**.

IR (KBr)


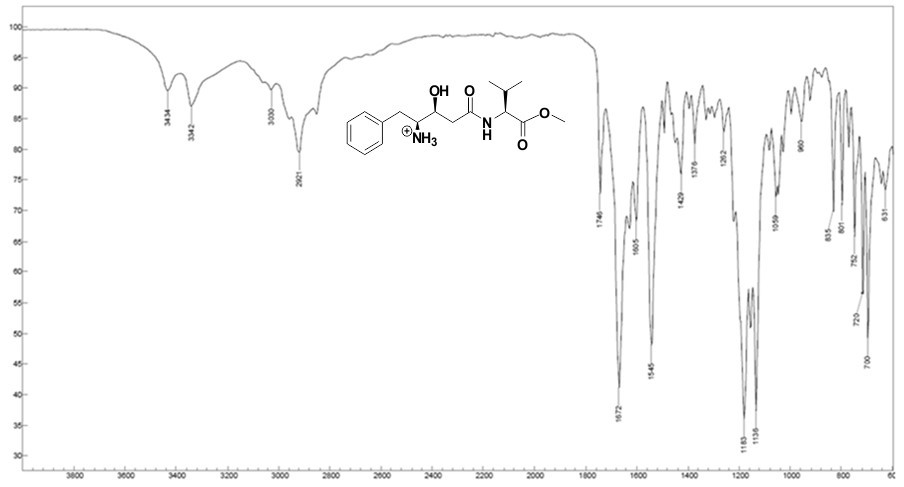


(9

g

)

^1^H NMR (500 MHz, DMSO-d_6_)


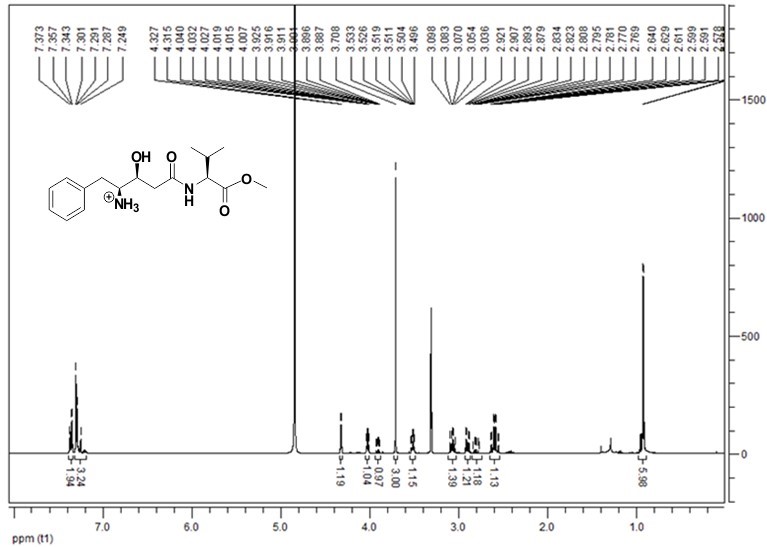


(9

g

)

APT (125.6 MHz, DMSO-d_6_)


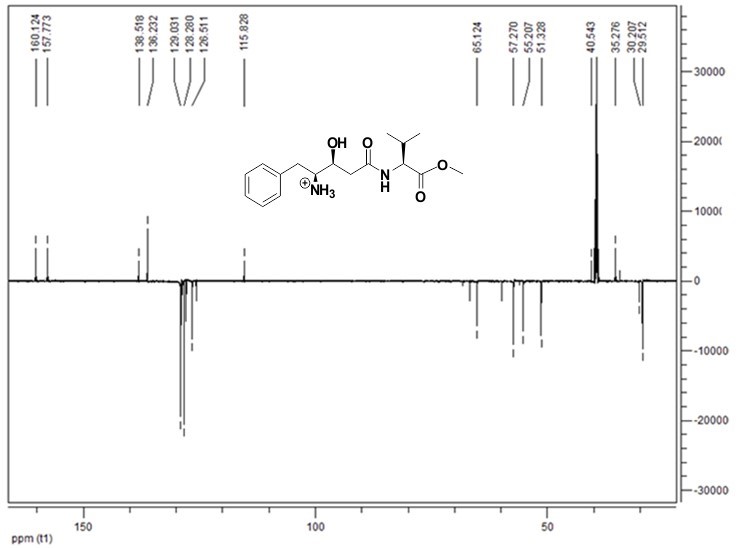


(9

g

)

ESI-HRMS


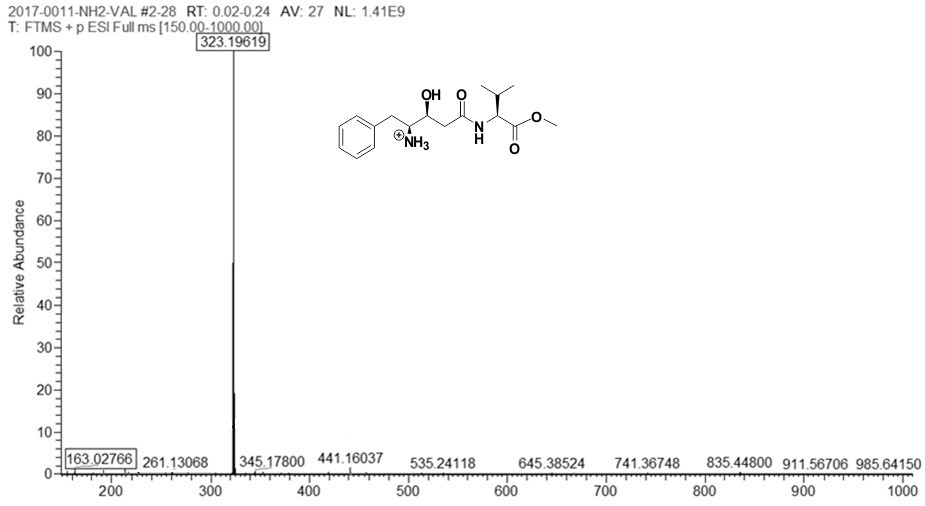


(9

g

)

# **Spectra of compound 10**.

IR (KBr)


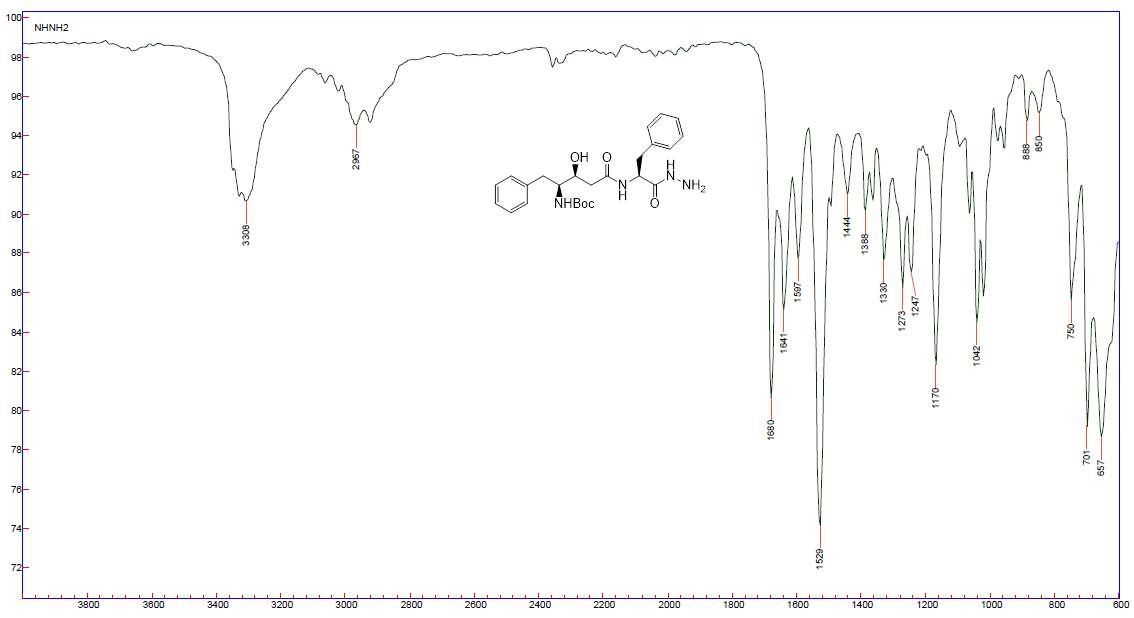


(10)

^1^H NMR (500 MHz, DMSO-d_6_)


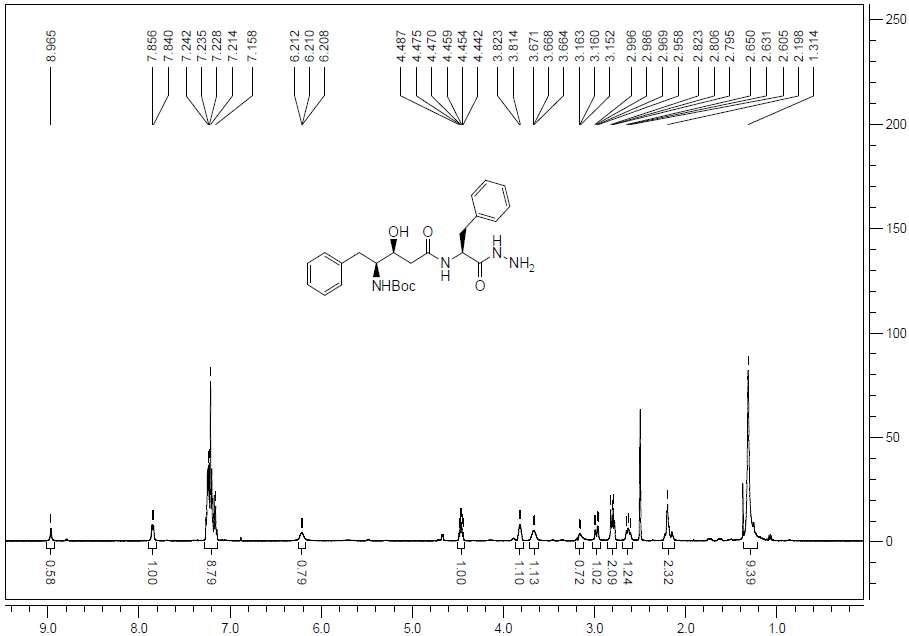


(10)

APT (125.6 MHz, DMSO-d_6_)


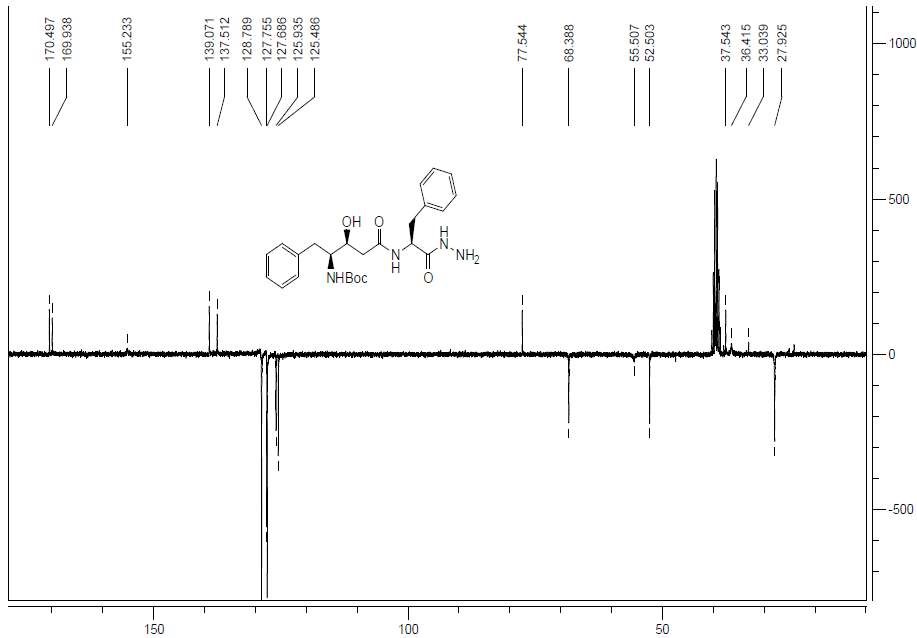


(10)

ESI-HRMS


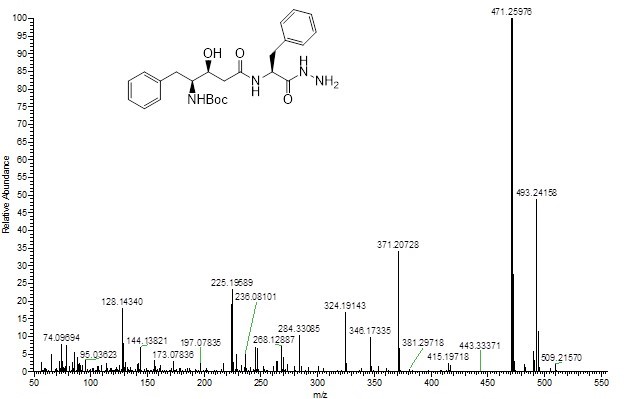


(10)
